# A nematode sterol C4α-methyltransferase catalyzes a new methylation reaction responsible for sterol diversity[S]

**DOI:** 10.1194/jlr.RA119000317

**Published:** 2019-09-23

**Authors:** Wenxu Zhou, Paxtyn M. Fisher, Boden H. Vanderloop, Yun Shen, Huazhong Shi, Adrian J. Maldonado, David J. Leaver, W. David Nes

**Affiliations:** Department of Chemistry and Biochemistry,* Texas Tech University, Lubbock, TX; Department of Biology, Geology, and Physical Sciences,† Sul Ross State University, Alpine, TX

**Keywords:** 8(14)-lophenol, *Caenorhabditis elegans *• biosynthesis, evolution

## Abstract

Primitive sterol evolution plays an important role in fossil record interpretation and offers potential therapeutic avenues for human disease resulting from nematode infections. Recognizing that C4-methyl stenol products [8(14)-lophenol] can be synthesized in bacteria while C4-methyl stanol products (dinosterol) can be synthesized in dinoflagellates and preserved as sterane biomarkers in ancient sedimentary rock is key to eukaryotic sterol evolution. In this regard, nematodes have been proposed to convert dietary cholesterol to 8(14)-lophenol by a secondary metabolism pathway that could involve sterol C4 methylation analogous to the C2 methylation of hopanoids (radicle-type mechanism) or C24 methylation of sterols (carbocation-type mechanism). Here, we characterized dichotomous cholesterol metabolic pathways in *Caenorhabditis elegans* that generate 3-oxo sterol intermediates in separate paths to lophanol (4-methyl stanol) and 8(14)-lophenol (4-methyl stenol). We uncovered alternate C3-sterol oxidation and Δ^7^ desaturation steps that regulate sterol flux from which branching metabolite networks arise, while lophanol/8(14)-lophenol formation is shown to be dependent on a sterol C4α-methyltransferse (4-SMT) that requires 3-oxo sterol substrates and catalyzes a newly discovered 3-keto-enol tautomerism mechanism linked to *S*-adenosyl-l-methionine-dependent methylation. Alignment-specific substrate-binding domains similarly conserved in 4-SMT and 24-SMT enzymes, despite minimal amino acid sequence identity, suggests divergence from a common, primordial ancestor in the evolution of methyl sterols. The combination of these results provides evolutionary leads to sterol diversity and points to cryptic C4-methyl steroidogenic pathways of targeted convergence that mediate lineage-specific adaptations.­­

Biosterols when converted to their geosterane counterparts provide the fossil record with a timeline for the origin and divergence of eukaryotic life. Equally significant, the emergence of C_27_-C_29_-Δ^5^ sterols in protists and in the geological record as C_27_-C_29_ steranes reflect the central importance of specific C4-desalkyl-24-desalkyl/24-alkyl sterols in cell vitality as architectural components of membranes (1–3). The structural and stereochemical diversity of these membrane inserts are largely derived from the action of sterol C24-methyl transferases (SMTs), which catalyze the carbocation-mediated methylation of Δ^24^-sterol substrates to generate an enormous variety of sterol side-chain-modified products (4, 5). 24-SMTs, considered to have arisen in the last eukaryotic common ancestor (6, 7) following the rise of oxygen perhaps as early as 1,600 million years ago (MYA) (8, 9), presumably have undergone diversification through single-product formation of C_28_ and C_29_ sterols or, as is more often the case in the Precambrian era, by a substrate-promiscuous or partitioning-explicit SMT yielding phyla-specific mixtures of C_26_ to C_31_ sterols that as steranes appear in the mid-Proterzoic to Phanerzoic eons, 550 to 1,200 MYA (10–14).

Despite the predominance of C24-alkyl sterols in eukaryotes and their absence in bacteria, we still do not know when and how rarely C4-methyl stanols and stenols can accumulate in a wide range of prokaryotic and eukaryotic organisms (**Fig. 1**) (15–17). As is sometimes possible, fossil steranes with the unprecedented 4-methylation in ring-A have been identified as molecular biomarkers in dinoflagellate evolution (14). C-4 methylated sterols are most frequently found to accumulate in nature as ring-C unsaturated compounds such as Δ^8(14)^-lophenol [4α-methyl cholest-8(14)-enol] synthesized in bacteria and its 24-methyl analogue in dinoflagellate algae, respectively (15, 16), or nuclear-saturated, as in dinosterol (C4α-methyl, 23,24-dimethyl ergost-22-enol), dicytosterol (C4α-methyl C24-ethyl poriferast-22-enol), and C24-methyl lophanol (C4,24-dimethyl cholestanol) synthesized in dinoflagellate algae and amoebae, respectively (17, 18). Biosynthetic reasoning suggests C4α-methyl sterols of eukaryotes are produced in similar fashion from protosterol (lanosterol in animals or cycloartenol in plants) (19). Typically, a single C4α-demethylase in eukaryotes removes the methyl group from a C4α/β-dimethyl- and C4α-methyl-sterol substrate, generating the 3-oxo sterol product, while bacteria utilize a catalytically distinct demethylase in the removal of the C4β-methyl group from lanosterol, yielding the equivalent C4α-methylated product (20).

**Fig. 1.**
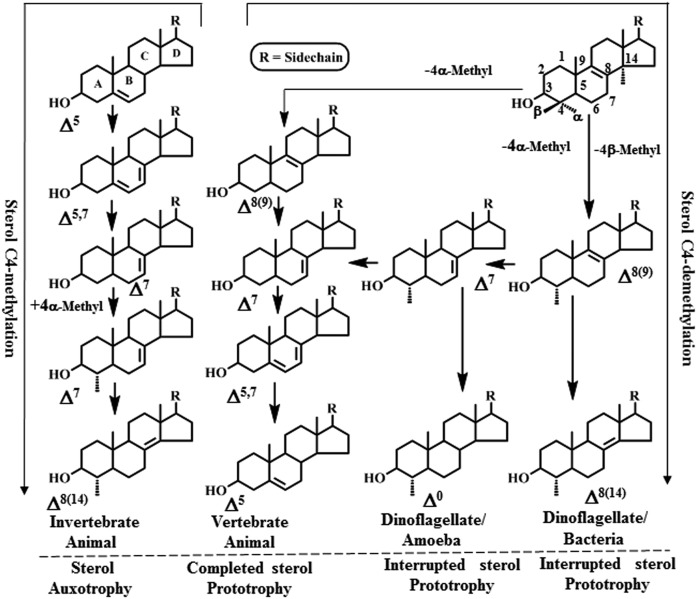
Conventional view of sterol biosynthesis and metabolism across the prokaryotic-eukaryotic domains; *R* = side chain of 8 [e.g., 8(14)-lophenol in bacteria and animals] to 10 (e.g., dinosterol in dinoflagellates and dictyosterol in amoebae) carbon atoms.

From an evolutionary perspective, there have been few biochemical insights into the molecular and enzymic determinants responsible for the uncommon product profiles typified by the C4-methyl sterol Δ^8(14)^-lophenol. The most comprehensive evidence for invertebrate cholesterol metabolism yielding Δ^8(14)^-lophenol is based on the sterol auxotroph* Caenorhabditis elegans* (21) that can synthesize C4-methyl sterols and dafachronic acid hormones (bile acid-like structures) (supplemental Figs. 1–3) (22–24). Accordingly, a downstream split in the pathway develops such that the main trunk continues to C4-methyl Δ^8(14)^-sterol production while the branch route proceeds to C4-desmethyl Δ^4^- or Δ^7^-3-oxo sterols; the latter compounds convert to dafachronic acid steroid hormones. The sterolic enzymes in *C. elegans* capable of Δ^8(14)^-lophenol production appear to be organized in reverse order and to possess substrate and reaction specificities distinct from related enzymes assembled in cholesterol biosynthesis pathways (Fig. 1). In contrast to C4-methyl sterol utilization in sterol prototrophs, in which the C4-methyl group(s) is necessarily removed in conversion to cholesterol for growth support (25, 26), in *C. elegans* the inclusion of a C4-methyl group on the metabolite product is essential for larval growth or to effect dauer formation (27–30). This work nevertheless leaves open questions regarding the number, properties, and evolutionary origin of sterol metabolases in nematodes, most notably the 4-SMT that controls the balance of neutral C_28_ to acidic C_27_ sterols in *C. elegans* (23); its substrate specificity and reaction mechanism remain enigmatic.

In the current study of *C. elegans* sterol synthetic abilities, we address these questions by integrating cell-based biochemistry, deuterium labeling, metabolite identifications, and cloned enzyme characterization. In first examining cholesterol metabolism to establish flux control, we identify a branch-point biosynthetic step Δ^7^ desaturation catalyzed by DAF-36p that regulates whether cholesterol converts to Δ^7^- and Δ^8(14)^-lophenols or to lophanol (Δ^0^-sterol) and show sterol C3-oxidase(s) generate 3-oxo sterol substrates as integral metabolites of the canonical synthetic pathway to 8(14)-lophenol. The full complement of seven distinct cholesterol metabolism enzymes in 8(14)-lophenol synthesis, including putatively two new ones, C3 and C4 reductase, predicted in flux studies of cholesterol metabolites and one from the original model, C5 desaturase, eliminated based on isotopic labeling studies, were annotated by bioinformatic analysis. As a result of the metabolite profiling that revealed the importance of 3-oxo sterols as intermediates directly and in crossover routes to 4-methyl stanol and stenol production, we cloned and characterized the nematode 4-SMT. We discovered for the first time that 4-SMT catalyzes a unique reaction mechanism and accepts 3-oxo sterols that define its product specificity and separation from 24-SMT enzymes. Intriguingly, genome screening of 4-SMT DNA show this class of catalyst can occur sporadically across eukaryotic kingdoms, suggesting sterol C4 methylation is a phylogenetic signature for sterol diversity and indicates biosynthetic convergence in C4-methyl steroidogenesis between bacteria that synthesize 8(14)-lophenol via a C4-methyl removal process as a primary metabolism and those eukaryotes that synthesize 8(14)-lophenol via a C4-methyl addition process as a secondary metabolism (Fig. 1). Moreover, the establishment of this newly described secondary steroidogenesis pathway in nematodes presents an opportunity for therapeutic applications targeting one or more sterolic enzymes in disease-causing nematodes that depend on C4-methyl sterols for growth or reproduction.

## MATERIALS AND METHODS

### *C. elegans* cultivation and growth

*C. elegans* strain N2 (*C. elegans* var. Bristol), *C. elegans daf-36* mutant, and *Escherichia* *coli* strain OP-51 were purchased from the Caenorhabditis Genetics Center. Sterol-depleted nematode growth medium (NGM) was prepared according to the published procedure except the peptone was extracted by HPLC-grade diethyl ether and the agar replaced by molecular biology-grade agarose (determined to be sterol-free) at 1.7% w/v. Synchronized N2-L1 worms (roughly 150) were distributed on 10 cm petri plates containing NGM supplemented with 5 mg/l cholesterol and then seeded with *E. coli*. The worms were cultured at 25°C for 6 days. The worms of mixed maturation from two petri dishes were collected and washed with M9 buffer three times and used as starting cultures to inoculate new petri dishes supplemented with a different nutrient sterol. For each test supplement, 10 petri dishes of worms were cultured at 25°C for 6 days; no statistical methods were used to predetermine sample size. At harvest, worms were washed from the petri dish and pelleted by centrifugation at 1,000 rpm for 5 min. The pooled worms were washed with PBS buffer (pH 7.5) three times and stored at −80°C until further use. Methionine labeling of sterols was performed using [^2^H_3_-*methyl*]methionine fed to worms at 1 mg/ml in the presence and absence of 5 mg/l sterol supplement using the culture protocol above. In the specialized feeding study of 6F-fluorocholesterol against cholesterol, worms were sterol-depleted by the extraction of N2 worms using the bleach/NaOH method and then incubated in M9 medium for 14 h. The resulting hatched L1 worms were added to NGM plates containing 5 mg/l sterol, and then the worms were incubated at 22°C for 48 h to permit predominantly L4 development. Microscopic visualization of worms was achieved using a stereomicroscope (AmScope) and photographed. The length of each worm was measured using ImageJ software (https:/imagej.nih.gov.ij) without calibration. The length of worms was expressed in number of pixels, and the stage recorded agreed with the published lengths for larval development (supplemental Fig. 2). For some incubation to follow the flux of isotopically labeled metabolite, distilled water in normal growth medium was replaced with deuterated water (20% D_2_O; v/v). For the latter experiments, larvae (typically about 150 per plate) were cultured at 25°C for 6 days. The worms were removed from the petri dish (usually 10) and collected by centrifugation at 1,000 rpm for 5 min. The worm pellets of mixed age (larvae and adults) were washed with PBS buffer (pH 7.5) three times and stored at −80°C until sterol analysis was performed. Deuterium enrichment in the affected sterol metabolites was determined by establishing the molecular ion cluster for the molecular ion (M^+^) of each labeled sterol compared with the corresponding unlabeled control sterol. The intensities of the extracted ion chromatogram from M^+^0 to M^+^9 (corresponding to D-atom incorporation from NADPD into sterol) of each sterol were manually integrated and tabulated. The isotope distribution was deconvoluted and calculated as percentage of total. In two independent experiments for each feeding study, minimal variation (less than 5%) was detected in the molecular ion clusters of targeted labeled metabolites.

### Statistical analysis

Nematode growth studies in response to sterol supplementation were analyzed by a Student’s *t*-test comparing the control to treated cultures (*n* = 10 independent trials). The SEM was determined to be statistically significant at *P* > 0.01.

### Chemicals and instrumental analysis

Most sterols fed to *C. elegans* were obtained from our earlier works (supplemental Tables 1 and 2) and were <95% pure by GC. For some feedings, new sterols were prepared or obtained commercially as described in the supplemental data. [^2^H_3_-*methyl*]methionine (98% atom enrichment) and cholesterol-2,2,3,4,4,6-*d*_6_ (97% atom enrichment) were purchased from Sigma-Aldrich. Tetraosylate [^2^H_3_-*methyl*]*S*-adenosyl-l-methionine (SAM) (99% atom enrichment) was purchased from C/D/N Isotopes. Deuterated water (^2^H_2_O; D_2_O) was purchased from Cambridge Isotope Laboratories (99% atom enrichment). The Bradford protein assay kit was purchased from Bio-Rad, and isopropyl-1-thio-β-d-galactoside was from Research Products International Corp. All other reagents and chemicals were from Sigma-Aldrich or Thermo Fisher Scientific unless otherwise noted. Instrumental methods for HPLC, TLC, GC/MS, and ^1^H/^13^C-NMR analysis have been described previously and are reported in detail for relevant chemical identifications in the supplemental data.

### Gene cloning: expression and activity assay of 4-SMT

The phage plasmid containing the yk401g2 cDNA clone from *C. elegans* was provided by Yuji Kohara (The National Institute of Genetics). The 1005 bp coding region of the *SMTR-1* (H14E04.1) gene was amplified from the yk401g2 cDNA clone using *Pfu* polymerase (Stratagene) with a forward primer cgggatcccgatgtccatcaatatgaatgccaac and a reverse primer cggaattccgtcagattttcttcttctcaaacagca (restriction sites underlined) and then inserted into the Gateway entry vector pENTR1A between BamH I and EcoR I sites to generate the pENTR1A-*SMTR-1* vector. The bacterial expression construct with the *SMTR-1* gene was generated through the recombination between the pENTR1A-*SMTR-1* vector and the bacterial expression vector pDEST17 using the Gateway LR Clonase II Enzyme Mix (Invitrogen). The resultant pDEST17-*SMTR-1* construct was then transferred into *E. coli* BL21(DE3) pLysS competent cells (Stratagene), and the 4-SMT was functionally expressed after a 4 h incubation of 400 µM isopropyl-1-thio-β-d-galactoside. Recombinant 4-SMT expressed from *E. coli* was prepared as for 24-SMT (7) except the activity assay contained 1.5 mg total lysate protein (Bradford estimation), 100 µM sterol (solubilized in Tween 80), and 200 µM SAM, and the reaction mixture was incubated overnight at 35°C (7) and then quenched by the addition of 10% methanolic KOH. The sample was extracted with hexane, and the enzyme-generated sterol was analyzed by GC/MS.

### Bioinformatics

The sterol 4- and 24-SMT sequences and related methyltransferases discussed in the supplemental data were retrieved from the NCBI (https://www.ncbi.nlm.nih.gov) using *STRM* (*C. elegans* sterol 4-methyltansferase) as an inquiry. The sequences were input into Geneious version R9.1.8 (Biomatters Ltd.) and aligned using the embedded MUSCLE program with the default setting. The phylogenetic tree was constructed using the Neighbor-Joining method with MEGA software version 7.0.26.

### GC/MS-based sterol profiling

For sterol analysis, the neutral lipids extracted from worm pellets following saponification were analyzed by GC/MS as previously described with cholestane as an internal standard (7). The GC/MS data were processed with Chemstation software (Agilent) and AMDIS (National Institute of Standards and Technology). The sterol peaks were deconvoluted using AMDIS after baseline correction and unequivocally identified by their coincidental retention time (observed in their retention time relative to the retention time of cholesterol) and identical EI-MS spectra at 70 eV like reference standards in our sterol collection or reference mass spectra from a commercial database (NIST08 mass spectral library). The GC peaks representing sterol amount generated from a total ion current chromatogram were integrated using the software default parameters.

### Isotope pattern deconvolution

Isotope pattern deconvolution of deuterium-labeled cholesterols was calculated from established GC/MS-based methods in the literature. Briefly, for sterols metabolized from the 2,2,3,4,4,6-*d*_6_ cholesterol supplement, the intensities of extracted ion chromatograms from molecular ion (M^+^) to maximum possible deuterium-labeled molecular ion [M^+^6]^+^ were integrated after baseline correction. The intensities of each ion were input in an Excel spreadsheet, and the isotope pattern deconvolution for each sterol was calculated after the correction to remove natural ^13^C isotope contribution.

### Synthetic sterols

Synthetic routes and GC/MS, TLC, and ^1^H NMR characterizations of relevant sterols as reference material or substrate for incubations with cloned 4-SMT or cell-based *C. elegans* feeding is shown in supplemental Figs. 4–9.

## RESULTS

### Discovery of coordinately branching pathways in *C. elegans* cholesterol metabolisms

**Fig. 2.**
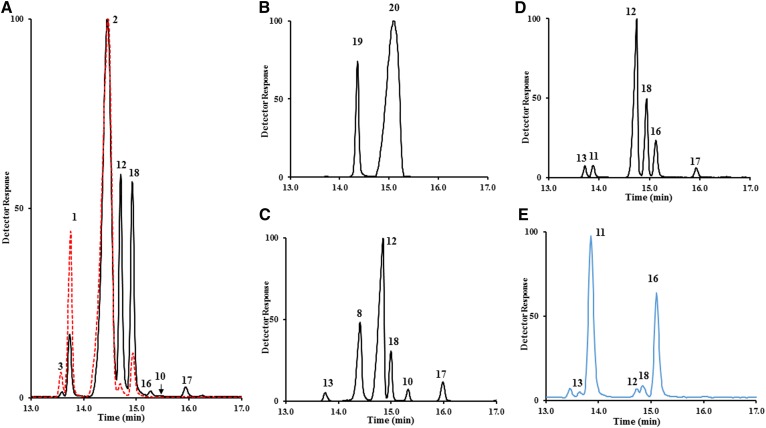
Total ion current chromatograms of representative sterol metabolisms by *C. elegans*. A: Total sterol of wild-type nematodes fed cholesterol (black) or D_6_-cholesterol (red). B: Total sterol of nematodes fed with 6-fluorocholesterol. C: Total sterol of nematodes fed with cholest-4,7-3-one. D: Total sterol of nematodes fed with cholest-4-one. E: Total sterol of *daf-36* mutant nematodes fed with cholesterol. Structures numbered in the GC trace are illustrated in Fig. 6.

**Fig. 3.**
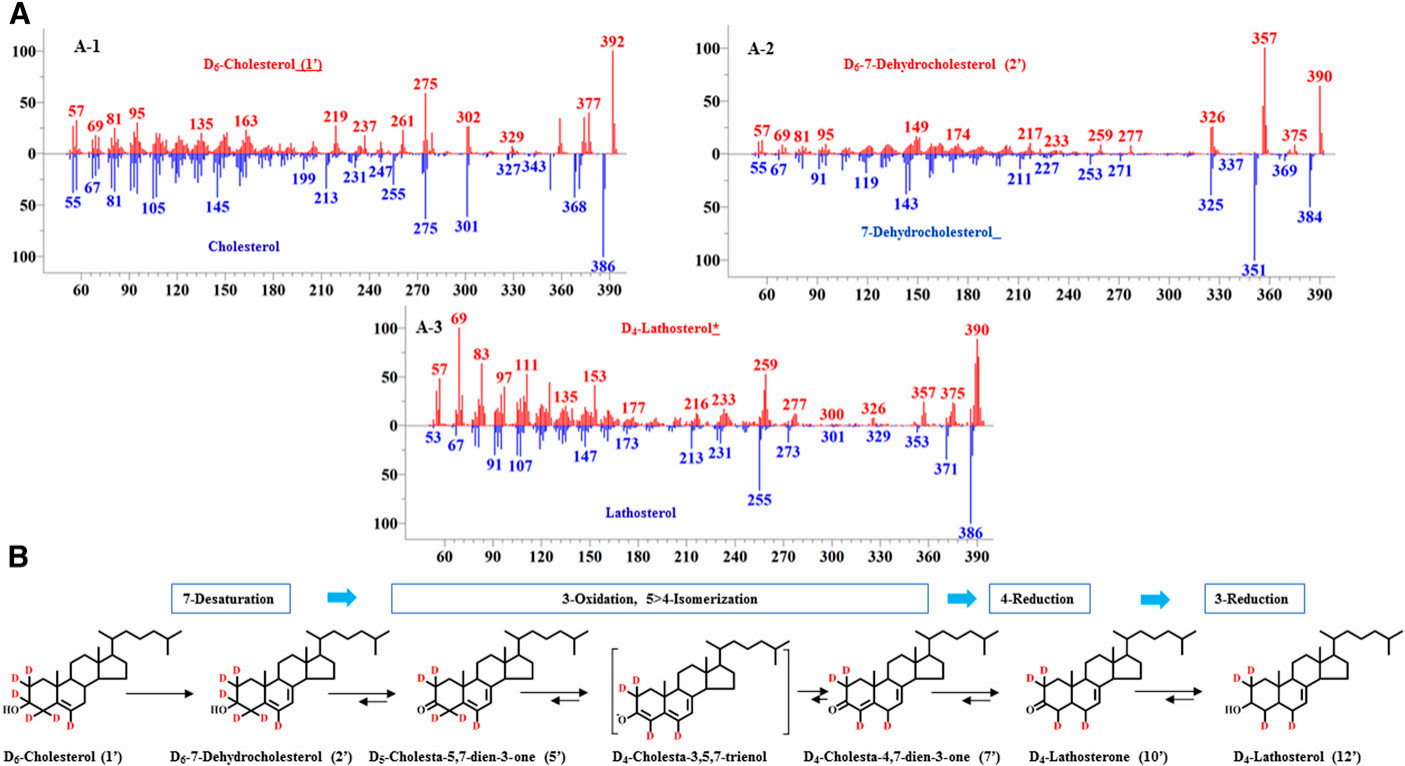
MS analysis of deuterated sterol metabolites correlated to a revised proposal for cholesterol metabolism to C4-methyl sterols in *C. elegans*. A: Mass spectra of control sterol metabolites and their deuterium-labeled counterparts D_6_-7-dehydrocholesterol [A-2; **2**′] and D_4_-lathosterol [A-3; **12**′] generated from D_6_-cholesterol [A-1; **1**′] metabolism in *C. elegans*. Typical sterol fragments corresponding to ions for M^+^, M^+^-CH_3_, M^+^-CH_3_-H_2_0, and M^+^ side chain in the high-mass end of the spectrum were present in both the control and deuterated sterols. Isotope pattern deconvolution confirmed the molecular weight of the deuterium-labeled metabolite. B: Our hypothetical biosynthetic scheme for D_6_-cholesterol conversion to D_4_-lathosterol in *C. elegans*; for an alternate metabolism of D_6_-cholesterol consistent with conventional understanding of cholesterol metabolism in nematodes, see supplemental Fig. 3.

Although there is much research in *C. elegans* sterol metabolism, the rationale for how the reported sterol complement generated in nematodes from cholesterol metabolism specifies the metabolite order, what enzyme(s) controls the flux to Δ^8(14)^-lophenol, and where stanols originate in an otherwise stenol metabolic pathway is poorly defined. In a preliminary study, to confirm previous sterol metabolism results (21), we characterized the sterol content of *C. elegans* fed with cholesterol and D_6_-cholesterol (**Fig. 2A**). Consistent with previous reports (21), cholesterol fed to the nematode generated cholesterol (cholest-5-enol) [**1**], 7-dehydrocholesterol (cholesta-5,7-dienol) [**2**], lathosterol (cholest-7-enol) [**12**], lophenol [**17**], and Δ^8(14)^-lophenol [4α-methyl cholest-8(14)-enol] [**18**]. However, quite surprisingly, the D_6_-cholesterol supplement generated a disrupted sterol composition that was presumably due to a kinetic isotope effect on an intermediate enzyme that led to marked depletion in lathosterol and C4-methyl sterol metabolites (Fig. 2A). An examination of the nature and extent of labeling in the deuterium-labeled precursor-product pair of 7-dehydrocholesterol and lathosterol indicated the former compound possessed 6-deuterium atoms (M^+^ 384 to M^+^ 390 amu), while the latter compound possessed 4-deuretium atoms (M^+^ 386 to M^+^ 390 amu) (**Fig. 3A**). If this metabolism was to proceed by direct reduction of the Δ^5^ bond, then the product should retain the original six deuterium atoms and therefore no kinetic isotope effect. To account for the 4-deuterium atom enrichment in lathosterol, we considered an alternative metabolism in which the previously characterized C3 oxidase (31, 32) is responsible for the 2-deuterium atom elimination from D_6_-cholesterol. For this reaction, the nematode enzyme is anticipated to incorporate two catalytic activities that according to the accepted cholesterol oxidase mechanism (33) acts stepwise, first in C3 oxidation (loss of C3 deuterium) and then in Δ^5^ to Δ^4^ isomerization (loss of C4 deuterium). The product of this catalysis is predicted to be D_4_-cholesta-4,7-dien-3-one; further processing of the D_4_-3-oxo derivative to lathosterol requires a pair of “new” enzymes, such that the Δ^4^-3-oxo derivative is reduced to yield D_4_-labeled lathosterone [= C4(5) reductase], and this product is C3-reduced (= C3-keto reductase) to yield D_4_-labeled lathosterol. The proposed route of deuterium elimination in substrate and subsequent metabolisms of D_6_-cholesterol to D_4_-lathosterol is shown Fig. 3B.

**Fig. 4.**
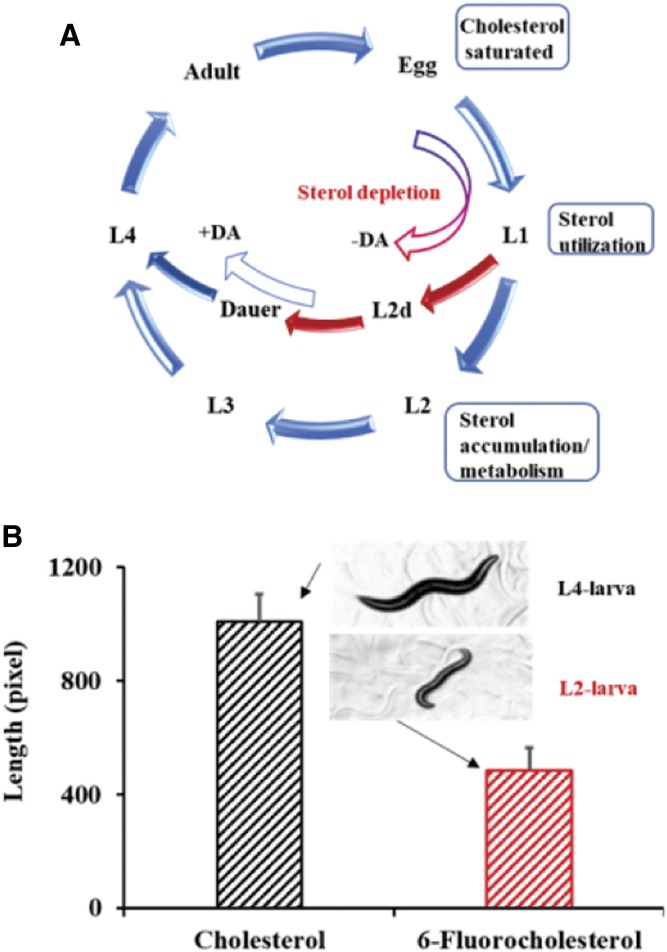
Life cycle of *C. elegans* regulated by sterol supplementation: A: Role of dietary sterol and dafachronic acid (DA) in *C. elegans* growth and maturation. B: Effect of cholesterol and 6-fluorocholesterol on *C. elegans* growth at 4 days; the error bar indicates the standard deviation for 10 independent feeding experiments.

To test the direct importance of C3 oxidation in cholesterol metabolism and nematode growth, we synchronously prepared and fed 6-fluorocholesterol to worms to be sterol-depleted at the time of sterol feeding (**Fig. 4**). We reasoned that fluorine substitution of hydrogen at C6 should have no effect on membrane suitability (34, 35); however, the highly electronegative atom should influence enzyme activity at the isomerization step involved with C3 oxidation, thereby reducing oxidase activity similar to the 24F-flourocycloartenol inhibition of sterol C24 methylation (36). GC/MS analysis of total sterol isolated from nematodes incubated with 6-fluorocholesterol (Fig. 2B) shows a single-metabolite 6-fluoro-7-dehydrocholesterol, suggesting the fluorinated substrate inhibits C3 oxidase activity, thereby preventing further metabolism. Nematode growth following 6-fluorocholesterol addition to L1 larvae was stalled in L2 larvae, which appeared as dauers in arrested development. In contrast, the cholesterol supplement promoted nematode growth to L4-stage growth (Fig. 4B, supplemental Fig. 2), and with additional incubation time the larvae become adults. These observations are supported by structure-activity studies that included 6-difluorocholesterol fed nematodes showing compound specifically induced L2 dauer formation (30).

We also observed a few percent of lophanol [**16**] in the total sterol fraction of cholesterol-fed nematodes (Fig. 2, supplemental Fig. 10), suggesting these worms possess a minimal cholesterol metabolism pathway to stanols. To investigate stanol metabolism further, next we used sterol substitution experiments of specific sterols fed to *C. elegans*. Defining what products result from a sterol substitution is a key first step in establishing rate-limiting steps, metabolite order, and expression in cholesterol metabolism pathways to stanols and stenols. The structures, chromatographic properties, and mass spectra of sterols examined in *C. elegans* are reported in supplemental Figs. 11 and 12 and supplemental Tables 1 and 2. Given 3-oxo sterols were candidates for metabolite intermediacy, we evaluated several 3-oxo sterols (supplemental Fig. 10) that differed in the extent of nucleus unsaturation for sterol metabolism. Nematodes fed with cholest-4-3-one and cholesta-4,7-dien-3-one turned the substrates over rapidly to distinct sets of stenol [lophenol and Δ^8(14)^-lophenol] and stanol (lophanol) C4-methylated products (Fig. 2C, D), although there was some crossover of the stanol intermediates into the stenol pathway but not vice versa. This result together with the kinetic isotope effect of D_6_-cholesterol and growth response from 6-fluorcholesterol indicate the C3 oxidase is rate limiting in cholesterol metabolism, and stanols can form an alternate pathway to the stenol pathway to C4-methyl sterols. In support of a stanol branch, after feeding cholesterol to the *C. elegans daf*-36 mutant deficient in Δ^7^ desaturase activity the sterol mixture changed to mostly cholestanol [**11**] and lophanol (4α-methyl cholestanol) [**16**] (Fig. 2E). Thus, branching of cholesterol metabolism into the stanol or stenol pathways is controlled by the expression of the Δ^7^ desaturase enzyme (Daf-36p). For these reasons, Daf-36p activity is key to whether 8(14)-lophenol or lophanol is the final metabolite of cholesterol metabolism in *C. elegans* and may be functionally significant in the disease cycle of pathogenic nematodes that depend on specific C4-methyl sterol products in growth or reproduction.

**Fig. 5.**
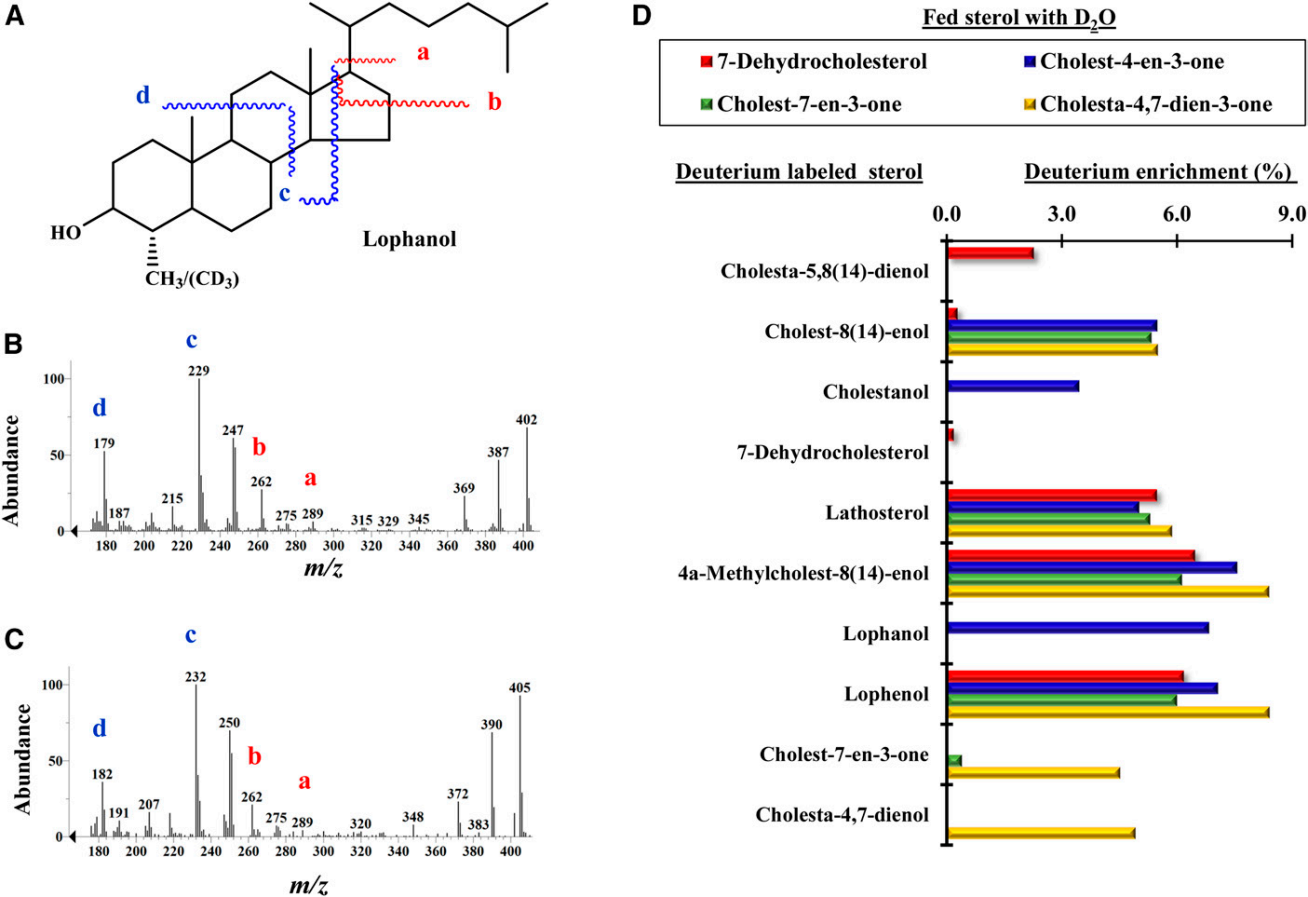
GC/MS analyses of labeled metabolites, including a separate characterization of deuterium-labeled lophanol from *C. elegans* fed [^2^H_3_-*methyl*]methionine paired with cholestanone [**9**]. A: Structure of lophanol [**16**] with relevant fragmentations. B: Control mass spectrum of lophanol showing ions associated with the side chain (red) and nucleus (blue). C: Mass spectrum of labeled lophanol with three deuterium atoms and associated relevant ions in color. D: Deuterium enrichment of metabolites observed in the GC analysis of the total sterol fraction of *C.* *elegans* cultured in D_2_O and fed 7-dehydrocholesterol [**2**], cholest-4-one [**4**], cholest-7-en 3-one [**10**], or cholesta-4,7-dien-3-one [**7**] in one typical experiment; for a key to structures see Figure 6. Note that the deuterium atom in D_2_O can exchange with the hydrogen atom in NADPH, yielding NADPD, which can then enter into steroidogenesis as a cofactor in reduction reactions delivering a deuterium atom to the sterol frame.

**Fig. 6.**
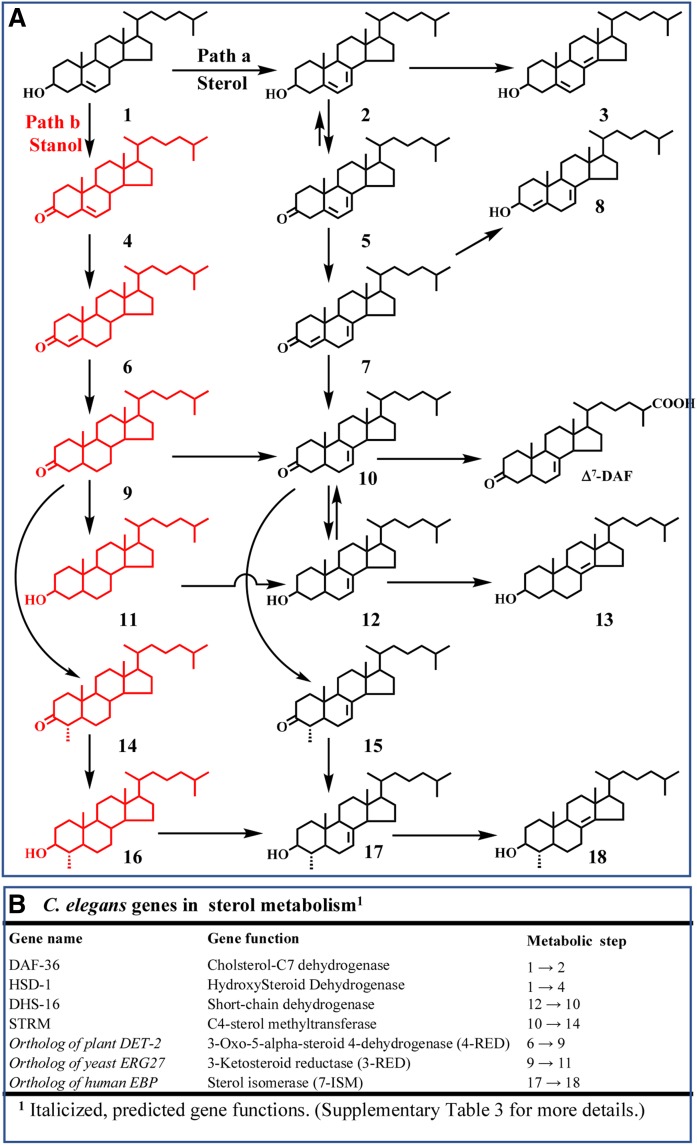
Proposed branching pathway of cholesterol metabolism in *C. elegans*. A: The stanol (path b) and stenol (path a) synthetic pathways to alternate C4-methyl sterol products. B: Hypothetical (in italics) and reported sterol genes in cholesterol metabolism to C4-methyl sterol products (for details on the bioinformatic analysis, see supplemental Tables 3 and 4). The successive six steps in cholesterol [**1**] metabolism to 8(14)-lophenol [4α-methyl cholest-8(14)-enol] [**18**] are proposed to proceed through sterol gene expression as follows: DAF-36, DHS-16, orthologue of *DET*-2, STRM, orthologue of *ERG*27, and orthologue of EPB.

**Figure 5** reports experiments of nematodes refed a set of sterol supplements cultured in D_2_O or with [^2^H_3_-*methyl*]methionine to provide additional information on biosynthesis, flow, and crossover in metabolite processing. These data show *i*) cholesta-4,7-dien-3-one [**7**] converts in the forward direction to cholesta-4,7-dienol [**8**] and lathosterol [**12**], showing 4-reduction and in the back direction to cholesta-5,8(14)-dienol [**3**]; *ii*) cholest-3-one (cholestanone) [**9**] converts to 4α-methyl cholestanol (lophanol) [**16**] as well as to 4α-methyl cholest-7-enol (lophenol) [**17**], showing metabolic crossover from the stanol to stenol pathway; *iii*) 4α-methyl cholestan-3-one (lophanone) [**16**] converts to lophenol for 3-reduction; and *iv*) incubation of cholestanone paired with [^2^H_3_-*methyl*]methionine produces deuterium-labeled lophanol of 3-deuterium atoms (Fig. 5A); the mass spectrum shows incorporation of deuterated methyl at C4 in the sterol A-ring. Transfer of the intact methyl from methionine in 4-SMT (STRM-1; sterol A-ring methylase-1) reveals a conserved role for SAM in the biosynthesis of methylated sterol in *C. elegans*. We surmise that cholesterol metabolism in nematodes involves two main branches utilizing seven cholesterol metabolism enzymes, four of which are known (DAF-36, HSD1, DHS16, and STRM) and three of which are predicted according to biosynthetic reasoning [sterol 8(9)- to 8(14)-isomerase, sterol C3 reductase, and sterol C4 reductase] (**Fig. 6**) and supported by bioinformatics analyses as shown in supplemental Tables 3 and 4.

### Demonstration for an ancient C4-sterol metabolism pathway catalyzed by *C. elegans*

The relationship of 24-methyl lophanol (4α-methyl ergostanol; 4,24-dimethyl cholestanol) to other sterols that appear in the fossil record as 4,24-dimethyl cholestane has been inferred only from dinoflagellate origins (9, 14). The retention of the C4-methyl group in these steroidal biomarkers is believed to represent a chemical signature for an interrupted de novo sterol metabolism that proceeds through the protosterol-lanosterol or cycloartenol or an alternate pathway that could involve 24-alkyl cholesterol that converts to the C4-methyl Δ^5^-sterol that is then processed via a keto-enol isomerization pathway, yielding the corresponding C4-methyl stanol (supplemental Fig. 13B) (37, 38).

Once it became clear to us that nematodes can synthesize a novel route to lophanol involving 3-oxo sterol intermediates we considered the observation by Chitwood that 24-methyl lophanol is likely a trace product of campesterol (24α-methyl cholesterol) fed to *C. elegans* and not necessarily an artifact (21). Consequently, we sought to verify this finding. We fed campesterol to *C. elegans* in the usual way and observed the nutrient was converted to metabolites that represent C24-methyl- and C24-desmethyl-sterol derivatives of the stenol pathway (supplemental Fig. 14). Careful inspection of the GC trace showed, as reported previously (21), a trace metabolite eluting at RRTc 1.27 with M^+^ 414, and its mass spectrum was consistent with the previously identified 24-methyl lophanol. However, within our limits of detection there was no GC/MS evidence for 3-oxo sterols. Clearly, had we used the *daf-*36 mutant where the Δ^7^ desaturase activity was knocked down; the abundance of 24-methyl lophanol and for that matter most any other side-alkylated sterol fed to the nematode would dominate the sterol mixture (supplemental Fig. 15). On the basis of these results and our characterization of metabolites in the various sterol feedings to nematodes, we considered C4-methyl stanols selectively formed in nematodes, dinoflagellates, amoebae, and other organisms could arise through a common C4-SMT that recognizes a C3-oxo sterol substrate.

### Elucidation of a cryptic sterol metabolism backward isomerization of Δ^8(9)^ to ^9(11)^

Zymosterol (cholesta-8,24-dienol), a key intermediate in animal cholesterol biosynthesis (19), was fed *to C. elegans* on the premise the 8(9) bond would move in the forward direction into the 8(14) position, affording a mixture of 3-hydroxyl and 3-oxo sterols; chemically, the tetra-substituted 8(14) bond is thermodynamically more stable than either the 7(8) or 9(11) position. Indeed, acid-induced isomerization of the 8(9) bond typically generates mostly 8(14)-sterol. To our surprise, zymosterol fed to *C. elegans* showed by GC/MS analysis six sterols, none of which were either cholest-8(14)-enol or cholesta-8(14),24-dienol or their 3-oxo sterol derivatives (supplemental Fig. 16). The first sterol in the chromatogram occurring in trace amount is cholesterol likely carried over from the egg. The remaining sterols in abundance are zymosterol (substrate) and four metabolites, generated in approximate 66% yield. The first metabolite in the GC trace possessed chromatographic properties and mass spectra that matched a standard of cholest-8(9)-enol (M^+^ 386), while identification of the four other metabolites is problematic because none of them possessed conventional RRTc, mass, and UV spectra for sterols characterized by us or available in the literature for a range of steroidal monoenes, dienes, and trienes (39–43). However, tentative identifications of a structure of unknowns could be made based on subtle differences in RRTc and in the ion abundances in mass spectra relative to reference material and through their distinct UV spectra in the HPLC-UV system (supplemental Fig. 17C). In HPLC, metabolites clustered into two main peaks that eluted before cholesterol. The UV spectra of the sterol mixture show end absorption and an uncommon, homoannular conjugated system with a λmax of 263 nm and an inflection at 273 nm. The UV λmax of the latter compound is shifted from that of a Δ^5,7^ system with a λmax of 282 nm or other typical conjugated heteroannular systems of Δ^7,9(11)^, Δ^7,14(15)^, or Δ^6,8(14)^ (λmax between 240 and 250 nm) or monenes (e.g., Δ^8^), which possess end absorption at 210 nm (supplemental Fig. 17C). Cholesta-5,7-dienol is a possible product of zymosterol metabolism, assuming the Δ^8(9)^ bond in zymosterol rearranges through a forward isomerization to the 7 position and the resulting product converts to a Δ^5,7^ compound with the assistance of a Δ^5^ desaturase. However, this route is deemed impractical because no sterol Δ^5^ desaturase is believed to be active in *C. elegans*. An alternative metabolism of zymosterol may proceed as follows: saturation of the 24(25) bond to form cholest-8-enol, followed by backward isomerization of the 8(9) bond to the 9(11) position, followed by ring-C7 desaturation to yield the Δ^7,9(11)^-diene intermediate, then another backward migration, this time of the Δ^7^ bond to the 8(14) position, which is followed by C4 methylation to yield the C4-methylated cholesta-8(14),9(11)-dienol product (proposed pathway shown in supplemental Fig. 18). It is worth pointing out that zymosterol-fed nematodes grew poorly, much like the nematodes cultured on 6-fluoro cholesterol, suggesting the 9(11)-sterol metabolic pathway is not physiologically acceptable to *C. elegans*.

### Characterization of a new class of methyltransferases

**Fig. 7.**
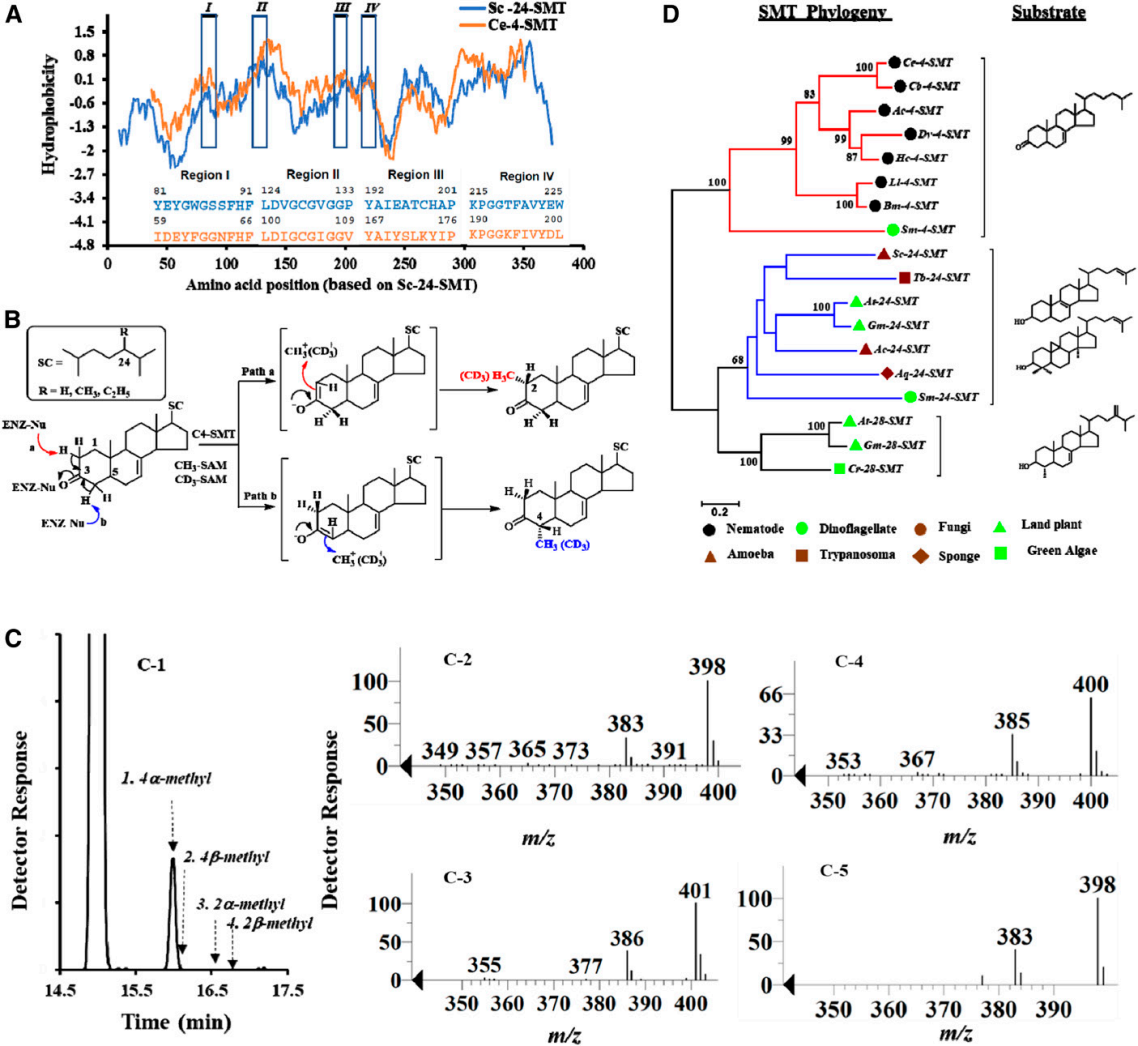
Hydropathy plot analyses, enzymology, and phylogenetics of sterol C4 methylation catalysts. A: Hydropathy plot comparison of *Ce*4SMT and *Sc*24-SMT, highlighting Regions 1–4 for the substrate binding segments. B: Proposed mechanism for the sterol C4 methylation pathway. C: GC trace of total sterol from *Ce*4-SMT incubated with cholest-3-one paired with SAM (C-1); reference synthetic standards of C4α/β-methyl 3-one and C2α/β-methyl 3-one sterols are shown in the GC trace. Mass spectrum of 4-SMT product C4-methyl cholest-7-3-one [**15**] following incubation of lathosterone [**10**] paired with SAM (C-2) or D_3_-SAM (C-3). Mass spectrum of 4-SMT product C4-methyl cholest-3-one [**14**] following incubation of cholest-3-one [**9**] paired with SAM (C-4). Mass spectrum of 4-SMT product C4-methyl cholest-5-3-one following incubation of cholest-5-3-one [**4**] paired with SAM (C-5). D: Phylogenetic relatedness and divergence of 4-SMT and 24-SMT enzymes in eukaryote evolution.

The *C. elegans* 4-SMT identified through mutant studies and bioinformatic screening (23) is proposed to be a relative of 24-SMT involved in the methylation of the sterol side chain because it shares three conserved SAM binding sites as identified by Kagan and Clarke (44). The SMT family of enzymes is a member of the methyltransferase enzyme class. These enzymes can possess very different substrate-specific methylation properties as determined kinetically and structurally through X-ray (45). A Blastp of the *C. elegans* genome sequences deposited in the NCBI database (https://blast.ncbi.nlm.nih.gov) using 24-SMT from *Saccharomyces cerevisiae* (=Erg6p, E.C. 2.1.1.41)-ONH72293 as a query yielded a single protein (NP_497549.2) with an E value at 1e^−29^ and 58% coverage that confirmed the previous annotation. The *C. elegans* 4-SMT has 334 amino acids of a predicted molecular weight of 37.8 kDa and shows 56% identity to other nematode 4-SMTs but 21% identity to Erg6p. The coding gene (H14E04.1) located on Chromosome III and associated exon partitioning resembles the 24-SMT gene architecture. The secondary structure prediction of *Ce*4SMT bears striking resemblance to *Sc*24SMT despite low sequence identity. A hydropathy plot for the two polypeptides (**Fig. 7A**, supplemental Fig. 19) highlighting the four substrate binding segments for sterol and SAM, identified by us through chemical affinity labeling and site-directed mutagenesis of Erg6p (46–49), shows a high degree of conservation in the three-dimensional structure of these enzymes, suggesting equivalence in the catalytic competence of 4-SMT and 24-SMT. The structural homology between 4-SMT and 24-SMT enzymes further suggests a common primordial ancestor early in the evolution of sterol biosynthetic pathways prior to the divergence of invertebrates from vertebrates.

However, because 2-methyl steranes as well as 4-methyl steranes have been detected in the fossil record (50, 51) and the Chitwood and Goodwin pathways (21, 37, 38) for C4-methyl sterol synthesis infer direct methylation (supplemental Fig. 1), the question arises whether the enzyme responsible for these C4 methylations could involve a substrate-based radical SAM reaction as proposed for the 2- and 3-methyl hopanoid synthesis (52, 53). Hopanoids are often considered sterol surrogates in bacteria (54), and their synthase genes have been related to oxidosqualene synthase genes in eukaryotes (55); therefore, methylases across the prokaryote-eukaryote divide could be so related. Our phylogenetic analysis shows that eukaryotic 4-SMT and prokaryotic 2-MHP are not closely related (<12% sequence identity), and in particular the 4-SMT amino acid sequence lacks the signature motif containing a B-12 binding domain involved in MHP synthesis (52); therefore, this radical reaction was ruled out in 4-SMT catalysis.

Another possibility is that the greater family of sterol methylases catalyzes similar electrophilic alkylation reactions in which the active site of 4-SMT provides proper steric-electronic topography for productive binding similar to the active site of 24-SMT. Sterol C24 methylation involves a carbocation mechanism requiring a transition state of cation-π interactions between charged methyl intermediates and aromatic residues in Region I-Erg6p = Y81EYGWG86. We discounted this reaction course because 4-SMT Region I does not contain the signature aromatic residues required for 24-SMT catalysis (supplemental Fig. 19) (5). On the other hand, the observation that nematodes can accumulate and convert C3-oxo sterols to C4-methyl sterol products suggested to us that the 4-SMT active site might have been remodeled during evolution, enabling a new chemistry that requires the substrate to rotate about its ligand axis, centering the bound catalytic group next to SAM and in contact with acidic residues that can promote the oxidation reaction. Accordingly, we hypothesized that a coupled C3-keto-enol isomerization and C4 methylation reaction is responsible for the stereoselective introduction of the methyl group into sterol ring-A (Fig. 7B). The unique aspects of this new sterol methylation property in *C. elegans* is underscored by our showing 3-oxo sterols incubated with a yeast or plant 24-SMT convert to 24(28)-methylene 3-keto sterol products; here, the 3-oxo group serves as a substrate anchor in the active site and does not participate directly in the methylation reaction (56).

To test the predicted methylase activity of the H14E04.1 protein, the nematode 4-SMT was cloned and expressed in *E. coli* (which does not synthesize sterols) (supplemental Fig. 20). When SAM was paired with 3β-OH sterols of cholesterol, lathosterol, cholestanol, or zymosterol, no products were detectable by GC/MS analysis, suggesting these sterols, including zymosterol favored by fungal 24-SMT, are not proper substrates. However, the incubation of 4-SMT with 3-oxo sterols of cholest-3-one, cholest-5-3-one, and cholest-7-3-one all resulted in the accumulation of unique products of identical retention time and mass consistent with the formation of 4α-methyl cholest-3-one (30% product), 4α-methyl cholest-5-one (10% product), and 4α-methyl cholest-7-3-one (30% product), respectively (Fig. 7C). Alternatively, nonproductive binding was observed in incubation with cholest-1-ene-3-one, cholest-4-ene-3-one, and cholest-4-enol. Incubation of recombinant 4-SMT with deuterium-labeled methyl SAM and cholestanone followed by saponification treatment gave a single deuterium-labeled product that was identified as C4-methyl cholestanone by comparing its retention time and mass spectrum to those of authentic standards. The labeled product contained three additional mass units (M^+^ 401) compared with the product of cholestanone paired with SAM that possessed a molecular weight of M^+^ 398 (Fig. 7). This observation strongly indicated that the 4-SMT catalyzes the direct methylation of the acceptor molecule and confirms the prediction that a substrate for this enzyme is a 3-oxo sterol. To determine the regio- and stereospecificity of the reaction, we prepared chromatographic standards of 4α-methyl, 4β-methyl, 2α-methyl, and 2β-methyl cholestanones. The GC trace of a control specimen convincingly shows a single product in the peak corresponding to C4α-methyl sterol (Fig. 7C).

Experimental results have unambiguously elucidated the biosynthetic mechanism of C4-methyl sterol formation as a two-step process of bound 3-oxo sterol undergoing active-site isomerization to the 4-enol intermediate that is methylated by SAM in a stereoselective manner to yield the C4α(equatorial) methyl group, followed by rearrangement of the enol back to the 3-oxo form as illustrated in Fig. 7B. Given the reaction pathway, it is equally possible for sterol methylation to generate the C2-methyl group in the sterol A-ring, which was not observed using our assay conditions. Our ability to determine contrasting catalytic abilities for 4- and 24-SMT enzymes reveal a new methylase functional type in 4-SMT. The enzyme-generated 3-oxo Δ^0^-, Δ^5^-, and Δ^7^-C4-methyl sterol products indicate the corresponding naturally occurring compounds and their C3-OH counterparts identified in sponges, zooxanthellae (dinoflagellates), amoebae, and worms (supplemental Fig. 21) could be synthesized by a 4-SMT, and the resulting cholesterol metabolites occur as secondary products in these organisms. This finding is the first reported observation of the biochemical features of a 4-SMT and of the reaction mechanism involved in the formation of C4-methyl sterols by a specific methyltransferase.

## DISCUSSION

In this work, we have systematically unraveled the branching pathways for cholesterol metabolism in *C. elegans* to C4-methyl stenol and stanol products and showed the rate-determining steps involved a sterol Δ^7^ desaturase and C3 oxidase. Following the conversion of cholesterol to 7-dehydrocholesteol, the Δ^5,7^-diene intermediate is C3-oxidized, affording the first 3-oxo sterol metabolite in cholesta-4,7-3-one [**7**], which then is reduced at C4, generating the second 3-oxo sterol metabolite in lathosterone [**10**]. As a tripartite intermediate, lathosterone can convert to Δ^7^-dafachronic acid or to lathosterol [**12**] or undergo methylation to form a third 3-oxo sterol metabolite in 4α-methyl cholest-7-one [**15**], which then is reduced to form lophenol [**17**]. Direct analysis of substrate and product levels for assessing enzyme activities in *C. elegans* indicates distinct isoforms of sterol C3-oxidases, seemingly complementary to the genes *HSD* and *DHS1*. These oxidation enzymes favor different substrate functional groups in the sterol nucleus of Δ^5,7^ or Δ^0^, enabling alternate cholesterol metabolisms to stenols and stanols. Chemical precedent for such substrate specificity is observed in sterol C3 oxidation reactions in which C3 oxidation of Δ^5^- and Δ^5,7^-3β-OH sterols requires oxidation reagents of different strength and reactivity to generate the desired outcome of Δ^4^- or Δ^5^-3-one or Δ^4,7^-3-one, respectively (57). Cometabolite reductions and *C. elegans* growth inhibition after supplementing 6-fluorocholesterol to the diet further indicate sterol C3 oxidase is a heretofore unrecognized rate-limiting enzyme at the start of cholesterol metabolisms in soil-borne nematodes. Additionally, the resulting 3-oxo sterols are necessary metabolites in 4-methyl sterol production. Interestingly, in marine invertebrates (e.g., Echinoderms and Cnidirians) incapable of de novo sterol biosynthesis (17), cholesterol metabolism is postulated to proceed to lathosterol via a Δ^5,7^-sterol and independently to generate cholestanol through a keto-enol isomerization pathway (17). These results suggest branching and regulation by oxidase and desaturase enzymes in nematode cholesterol metabolism may extend to other invertebrates as well.

Positioned as a metabolic branch point to supply essential end products, sterol C4 methylation yielding eukaryotic 8(14)-lophenol contrasts with sterol C4 demethylation yielding prokaryotic 8(14)-lophenol. These conflicting biochemical traits further contrast with the biosynthetic routes involved in C4-desmethyl-sterol biosynthesis yielding cholesterol and 24-alkyl sterols in plants, fungi, and protozoa. Clearly, the formation of 8(14)-lophenol converges in C4-methyl steroidogenesis across the prokaryotic-eukaryotic divide (58). Biosynthesis of 8(14)-lophenol in *C. elegans* includes six steps that operate in sequence from cholesterol as protosterol (Fig. 6, path a); two of these enzymes identified through biochemical analysis, the C4 reductase and C3 reductase, represent newly discovered cholesterol metabolism enzymes operating in *C. elegans*. These peculiar enzymes in invertebrates act in reverse to the enzyme order in primary metabolism in cholesterol synthesis in vertebrates and possibly originate from orthologues synthesized in sterol prototrophs that emerged as early as 1,600 MYA (supplemental Fig. 22). Intriguingly, the sterane biomarkers detected in evolution clearly record the absence of intermediates in the sterol biosynthesis pathway, including cycloartane/lanostane, showing instead the accumulation of a final Δ^5^-sterol product, much like that observed in extant organisms that are formed by different routes (58). These observations of directed metabolism are consistent with phylogenomic analyses of sterol genes that suggest all of the primordial enzymes necessary for Δ^5^-sterol production, including 24-SMT, were present in the last eukaryotic common ancestor (6).

Our studies in sterol methylation represent a paradigm shift in our understanding of steroidogenesis and suggest the emergence of 4-SMT in algae underlies evolutionary expansion in sterol-specialized metabolism. Such stepwise primary and secondary sterol evolutions seemingly developed from sterol methylation enzymes that obtained distinct substrate preferences and reaction specificities through SMT mutation and functional divergence. This is supported in the fossil record by the identification of C4-methyl steranes (lophanane, dinosterane, etc.) that appear after the identification of 24-methyl/ethyl C_27_-C_29_ steranes in the most primitive plants (supplemental Fig. 23). Discontinuity in the distribution of 8(14)-lophenol and C4-methyl stanols across phylogeny as evidenced in the co-occurrence of a 4-SMT and 24-SMT gene in dinoflagellates (Fig. 7) and the singularity of 4-SMT in nematodes (determined using *C. elegans* C4-SMT BLAST of the genome of all metazoans deposited in the NCBI database) indicates repeated gain and loss of the sterol C4 methylation pathway during the course of algal/protozoan evolution into invertebrate animals. This leads to the continuing diversification in sterol secondary metabolism, yielding lineage-distinct 24-methyl and 4-methyl sterol patterns in eukaryotes and paves the way for the development of new mechanism-based inhibitors (58) that target sterolic enzymes in related parasitic nematodes (Strongyloides) (59) capable of cholesterol metabolism as shown in Fig. 6.

## Supplementary Material

Supplemental Data
