# Supplementary material for: A nematode sterol C4α-methyltransferase catalyzes a new methylation reaction responsible for sterol diversity
Source: J Lipid Res. 2019 Sep 23;61(2):192–204. doi: 10.1194/jlr.RA119000317 (PMC6997595; doi:10.1194/jlr.RA119000317)
Supplement: Supplemental Data [file supp_61_2_192__index.html]

A nematode sterol C4α-methyltransferase catalyzes a new methylation reaction responsible for sterol diversity. — Sterol 4-methylation biosynthesis pathways in C. elegans — A nematode sterol C4α-methyltransferase catalyzes a new methylation reaction responsible for sterol diversity — Supplemental Data 

# A nematode sterol C4α-methyltransferase catalyzes a new methylation reaction responsible for sterol diversity

## Supplemental Data

- Identification of nematode 4-methyl SMT that catalyzes a new methylation reaction responsible for sterol diversity - FIle contains 24 figures and 6 tables.
- Supp Data - Supp Data
