# Supplementary material for: A nematode sterol C4α-methyltransferase catalyzes a new methylation reaction responsible for sterol diversity
Source: J Lipid Res. 2019 Sep 23;61(2):192–204. doi: 10.1194/jlr.RA119000317 (PMC6997595; doi:10.1194/jlr.RA119000317)
Supplement: Supplemental Data [file supp_RA119000317_155008_1_extended_data_396667_pxzg8c.pdf]

Supplementary Figures (23) and Tables (6)

Identification of a nematode 4-SMT that catalyzes a new methylation reaction responsible for sterol diversity

Wenxu Zhou, Paxtyn M. Fisher, Boden H. Vanderloop, Yun Shen, Huazhong Shi, Adrian J. Maldonado, David J. Leaver and W. David Nes

Tables (4 in succession and 2 associated with figures):

Supplementary Table 1. GC-MS analytics of sterols involved with this study.

**Sterols**	**Structures**[1]	**Source**[2]	$\boldsymbol{RRT_C}^{3}$	$\mathbf{M^{+4}}$
Cholesterol	1	TL	1.00	386
7-Dehydrocholesterol	2	TL	1.05	384
Cholesta-5,8(14)-dienol	3	TL	0.99	384
Cholest-5-en-3-one	4	TL	1.21	384
Cholesta-5,7-dien-3-one	5	TL	1.19	382
Cholest-4-en-3-one	6	TL	1.20	384
Cholesta-4,7-dien-3-one	7	TL	1.26	382
Cholesta-4,7-dienol	8	TL	1.02	384
Cholest-3-one	9	TL	1.07	386
Cholest-7-3-one (Lathosterone)	10	TL	1.12	384
Cholestanol	11	TL	1.01	388
Cholest-7-enol (Lathosterol)	12	TL	1.06	386
Cholest-8(14)-enol	13	TL	1.00	386
4α-Methylcholst-3-one	14	TL	1.10	400
4α-Methylcholst-7-en-3-one	15	TL	1.19	398
4α-Methylcholstanol (Lophanol)	16	TL	1.10	402
4α-Methylcholst-7-enol (Lophenol)	17	TL	1.16	400
4α-Methylcholst-8(14)-enol (8(14)-Lophenol)	18	TL	1.09	400
6-Fluorocholesterol	19	TL	1.04	404
6-Fluoro-7-dehydrocholesterol	20	TL	1.10	402
2α-Methylcholest-3-one	21	TL	1.17	400
2β-Methylcholest-3-one	22	TL	1.24	400
4β-Methylcholest-3-one	23	TL	1.29	400
Cholesta-8,24-dienol (Zymosterol)	24	TL	1.10	384
Cholest-4-enol	25	CM	0.97	386
Cholest-1-en-3-one	26	CM	1.09	384
Cholesta-4,6-dien-3-one	27	CM	1.46	382
2α-Methylcholestanol	28[4]	TL	1.12	402
4α-Methylcholest-5-en-3-one	32	TL	1.22	398

[1]See supplementary Figure 6 for key to structures

[2]TL, this laboratory, CM, commercial source.

[3,4]Retention time of sterol relative to the retention of cholesterol in GC; M^+ is the molecular ion determined in mass spectroscopy equivalent to the molecular weight of compound.

Supplementary Table 2. Chromatographic and spectral characteristics of standards from 1986.[1,2]

**Sterol**	**Structures**[3]	***RRT*c**	α_c	R_f	M^+	**UV (λmax, nm)**
Cholesterol	1	1.00	1.00	0.33	386	E.A[2].
Cholest-4-enol	25	0.97	0.94	0.38	386	E.A.
Cholest-5-en-3α-ol	29	0.95	0.87	0.56	386	E.A.
Cholest-4-en-3α-ol	30	0.95	0.80	0.56	386	E.A.
Cholest-5-en-3-one	4	1.30	1.02	0.83	384	E.A.
Cholest-4-en-3-one	6	1.32	0.87	0.83	384	241
Cholesta-1,4-dien-3-one	31	1.45	0.55	0.45	382	243
Cholest-3,5-dien	33	0.60	1.96	0.99	368	242
Ergosta-5,7,22-trien-3β-ol	34	1.22	0.76	0.30	396	282
Ergosta-4,6,22-trien-3β-ol	35	1.59	0.77	0.38	396	240
Ergosta-4,6,22-trien-3-one	36	1.57	0.79	0.65	394	281
Ergosta-4, 7,22-trien-3-one	37	1.57	0.68	0.64	394	238
Cholesta-3,5-dien-3-ol acetate	38	1.52	2.18	0.91	426	232
Cholesta-5,24(28)-dien-3β-ol	39	1.26	0.83	0.33	398	EA.
Cholest-8-en-3β-ol	40	1.06	0.95	0.33	386	E.A.
Lanosta-8,24(25)-dien-3β-ol	41	1.65	1.30	0.55	426	E.A.
Lanost-8-en-3β-ol	42	1.52	1.26	0.55	428	E.A.

[1]Data from Le, P. H., and W. D. Nes. 1986. Tritium Labeling and Selective Oxidations. *Chem. Phys. Lipids* **40**: 57-69 GC RRTc's may differ here from those in Supplementary Table 1 due to slightly different GC columns and operating conditions used in the studies. Refer to the paper for details on methods and instrumentations.

[2]RRTc determined by GC, R_f determined by TLC, M+ determined by mass spectroscopy and absorption λmax determined by UV using a HPLC-UV system equipped to a diode array detector; E.A. is end absorption around 210 nm.

[3]Structures are shown in Supplementary Figure 11.

Supplementary Table 3. Distribution of genes for sterol side chain and nucleus metabolizing enzymes in nematodes[1]

	Gene name	*Caenorhabditis elegans*	*Caenorhabditis briggsae*	*Ancylostoma ceylanicum*	*Haemonchus contortus*	*Dictyocaulus viviparus*	Loa loa	*Brugia malayi*
1	**DAF-36**	C12D8.5	XP_002637057.1	EYB92819.1	CDJ98259.1	KJH52421.1	XP_020305107.1	CDQ08010.1
2	**HSD-1**	Y6B3B.11	XP_002640554.1	EYC08685.1	CDJ81313.1	KJH47292.1	XP_003144602.1	CDP95353.1
3	**DHS-16**	C10F3.2	XP_002636827.1	EYB91040.1	CDJ87155.1	KJH46704.1	XP_003144399.1	CRZ22731.1
4	**STRM-1**	H14E04.1	CAP33846.2	EYC26550.1	CDJ90475.1	KJH45819.1	XP_020303948.1	XP_001900512.1
5	**ERG 27[3]**	Not detected						
6	**Det2[4]**	F42F12.3	XP_002645262.1	EYC20214.1	CDJ87906.1	KJH48701.1	XP_020302395.1	CDQ05357.1
7	**ERG2/Sigma1 receptor like protein[5]**	W08F4.3	XP_002632220.1	EYC01719.1	CDJ87547.1	KJH44980.1	XP_020306186.1	XP_001900183.1
8	**DIMINUTO-LIKE PROTEIN[6]**	F52H2.6	XP_002643854.1	EYC22973.1	CDJ96338.1	KJH41761.1	No Hit[2]	No Hit
9	**DAF-9**	T13C5.1	XP_002644834.1	EYB87637.1	CDJ82330.1	KJH50399.1	XP_003138701.1	XP_001899881.1

[1]The genes were retrieved by blasting the genomes in deposited in NCBI using *C. elegans* genes or predicited sterolic genes identified through other sources as queries. Genes and corresponding enzyme 1 to 4 are characterized and discussed in the text (cf. Fgure 6).

[2]The E value was greater than the threshold at e^{-10}.

[3] Note, a C3-reductase gene (e.g. ERG27) biochemically evident in *C. elegans* and present in sterol biosynthesis pathways across kingdoms is variably detected in nature using phylogenomics., *cf.* Desmond, E., and S. Gribaldo, 2009. Phylogenomics of sterol synthesis: Insights into the origin, Evolution, and diversity of a key eukaryotic feature. *Genome Biol. Evol*. **1**: 364-381.

[4]Reference: Noguchi T, S. Fujioka S, Takatsuto , A. Sakurai , S.Yoshida, J., Li, and J, Chory. 1999.. Arabidopsis det2 is defective in the conversion of (24R)-24-methylcholest-4-en-3-one to (24R)-24-methyl-5α-cholestan-3-one in brassinosteroid biosynthesis. *Plant Physiol*., **120**: 833-840.

[5]Reference: Vinci G, X. Xia, and R. A. Veitia. 2008. Preservation of Genes Involved in Sterol Metabolism in Cholesterol Auxotrophs: Facts and Hypotheses. *PLoS One* **3**: e2883.

[6] Reference: Ciufo LF, P. A. Murray, A. Thompson, D. J. Rigden, and H. H. Rees. 2011. Characterisation of a Desmosterol Reductase Involved in Phytosterol Dealkylation in the Silkworm, *Bombyx mori*. *PLoS One* **6**: e21316. Here, the gene/enzyme is predicted based on our *C. elegans* feeding study of zymosterol (cholesta-8,24-dienol) yielding cholest-8-enol that thereby shows reduction of the Δ^{24}-bond.

For DAF-9, the enzyme oxidizes sterol C26 of the 3-oxo sterol substrate involved in dafachronic acid synthesis- see for example- Jia, K., P.S. Albert, and D.L. Riddle. 2002. DAF-9, a cytochrome P450 regulating *C. elegans* larval development and adult longevity. *Development* **129**: 221-231.

Supplementary Table 4. Comparison of enzymes involved in Δ^5 sterol biosynthesis across kingdoms.

	Function	Gene Name			Enzyme name	EC#
		H. sapiens	*A. thaliana*	*S. cerevisiae*		
**1**	Oxidosqualene cyclization	LSS	CAS	ERG7	Lanosterol/Cycloartenol synthase	5.4.99.7/8
**2**	C32-Elimination	CYP51A1	CYP51G1	ERG11	Sterol 14α-methyl demethylase	1.14.13.70
**3**	C14-Reduction	DHCR14 (TM7SF2)	FK	ERG24	Sterol Δ^{14}-reductase	1.3.1.70
**4**	C4-Methyl oxidation	SC4MOL	SMO1	ERG25	Sterol C4-methyloxidase	1.14.13.72
**5**	C4-Methyl acid elimination	NSDHL	AT3βHSD/D1	ERG26	3β-Hydroxy- Δ^5-steroid dehydrogenase	1.1.1.170
**6**	C3-Ketoreduction	HSD17B7	Predicted[1]	ERG27	3β-Keto-reductase	1.1.1.270
**7**	Δ^8-Δ^7 Isomerization	EBP	HYD1	ERG2	Sterol C8 isomerase	5.3.3.5
**8**	Δ^5-Desaturation	SC5DL	DWF7	ERG3	Sterol C5-desatruase	1.14.19.20
**9**	Δ^7-Reduction	DHCR7	DWF5	NA	Sterol Δ^7 reductase	1.3.1.21
**10**	Δ^{24}-Reduction	DHCR24	DWF1	ERG4	Sterol Δ^{24}reductase	1.3.1.72
**11**	9β, 19β-cyclopropane ring opening	NA[2]	CPI1	NA	Cycloeucalenol cycloisomerase	5.5.1.9
**12**	C22-Desatruation	NA	CYP710A	ERG5	Sterol C22 desaturation	1.14.19.41
**13**	C24/28-Methylation	NA	SMT1/2/3	ERG6	Sterol C24/28-Methylase.	2.1.1.41/143

[1] Gene is not identified for this enzyme, although it is proven by biochemical/genetic approaches to be in sterol metabolism.

[2] N.A. = Not available; in humans the gene is not considered to be synthesized in the genome.

Legend to Figures

Supplementary Figure 1. Proposed pathways for metabolism of Δ^5-4-desemthyl sterol to 4-methyl sterol. Note- compound numbers in Supplementary Figure. 1 are not met to correlate with any other set of compound numbers in this paper.

Panel A is nematode pathway for phytosterol- sitosterol conversion to cholesterol, which is converted to 4α-methyl cholest-8(14)-enol (Adapted from Chitwood, D. J. 1983. Recent developments in nematode steroid biochemistry. *J. Nematolog*. **18**: 9-17 and Chitwood, D. J. 1983. Novel nuclear methylation of sterols by the nematode *Caenorhabditis elegans*. *Steroids* **42**, 311-319.

Panel B is the dinoflagellate pathway for Δ^5-24-alkyl sterol conversion to C4-methyl Δ^5-sterol intermediate that converts to 4-methyl 24-alkyl stanol (Adapted from (i) Withers, N. W., R. C. Tuttle, G. G. Holz, D. H. Beach, L .J. Goad, and T. W. Goodwin. 1978. Dehydrodinosterol, dinosterone and related sterols of a non-photosynthetic dinoflagellate, *Crypthecodinium-cohnii*. *Phytochemistry*. **17**: 1987-1989 and (ii) Withers, N. W., W. C. M. Kokke, W. Fenical, and C. Djerassi. 1982. Sterol patterns of cultured zooxanthellae isolated from marine-invertebrates - synthesis of gorgosterol and 23-desmethylgorgosterol by aposymbiotic algae. *Proc. Natl. Acad. Sci. USA* **79**: 3764-3768.

Supplementary Figure 2. Life cycle of *C. elegans* from egg to adult controlled by sterol and steroid hormones (dafachronic acid = DA) (Adapted from (i) Martin, R. E. V. Entchev, T. Kurzchalia, and H-J. and Knölker. 2010. Steroid hormones controlling the life cycle of the nematode *Caenorhabditis elegans*: stereoselective synthesis and biology. *Org. Biomol. Chem*. **8**: 739-750 and (ii) Aquilaniu, H., Fabrizio, P. and Witting. 2016. The role of dafachronic acid signaling in development and longevity in *Caenorhabditis elegans*: Digging deeper using cutting edge analytical chemistry. *Front. Endocrinol*. **7**, 1-15.

Supplementary Figure 3. Conventional cholesterol metabolism pathways in *C. elegans* that branch to form dafachronic acid and lophenol-identified sterols genes are so indicated (Adapted from Aquilaniu, H., P. Fabrizio, and M. Witting. 2016. The role of dafachronic acid signaling in development and longevity in *Caenorhabditis elegans*: Digging deeper using cutting edge analytical chemistry. *Front. Endocrinol*. **7**: 1-15 and references cited). **Panel A**: Proposed cholesterol metabolism pathway based on recent cloning of HSD, DAF36 and STRM genes. **Panel B**: Proposed deuterium labeling pathway for D_6-cholesterol to lathosterol in *C. elegans* based on the convetional sterol metabolism pathways illustrated in Supplementary Figure 1.

Supplementary Figure 4. Synthetic route to C4-methyl stanol production.

Supplementary Figure 5. Chromatographic analysis of total C4-methyl 3-oxo stanol mixture generated by synthesis: Panel A, C4-methyl 3-oxo stanols identified by GC-MS, Panel B, mass spectra of sterols detected in GC. Panel C TLC (benzene-ether, 85/15 2 developments) analysis of synthetic C4-methyl 3-oxo-stanol mixture.

Supplementary Figure 6. ^{1}H-NMR spectra of C4-methyl 3-oxo stanols recovered sequentially in four fractions from HPLC A to D (Luna reversed phase C_{18}-column eluted with 100 %

methanol at ambient temperature): Panels A and C represent pure compound while Panels B and D are mixtures with the α/β-isomer identities evident in the relevant chemical shift for C2 and C4-methyl doublets. The eluted sterols were re-run on GC-MS to confirm identifies against literature values (c.f. Goad, L. J. and T. Akihisa, T. Analysis of Sterols.1997. Blackie Press, London. 437 pp and references cited therein).

Supplementary Figure 7. GC-MS analysis of C4/C2-methyl stanol mixture generated by chemical reduction of the corresponding C3-oxo compounds (Panel A) and ^{1}H NMR of the HPLC pure lophanol (Panel B).

Supplementary Figure 8. Synthetic scheme and MS and NMR characterization of 6-fluorocholesterol.

Supplementary Figure 9. Synthetic scheme for Δ^5- and Δ^4- versus $\Delta^{5,7}$ and $\Delta^{4,7}$-3-oxo stenols and products purified by TLC/HPLC as reported in Le, P. H., and W. D. Nes, 1986. Sterols: tritium labeling and selective oxidations. *Chem. Phys. Lipids* **40**: 57-69 (1986).

<u>Supplementary- sterol synthesis details</u>.

All reactions (non-aqueous) were performed under an inert atmosphere of N_2 unless stated otherwise. Aluminum isopropoxide was purified via distillation as was toluene which was first dried with calcium sulfate and then distilled over molecular sieves. Cholesterol and 6-ketocholestanol were purchased from Sigma Aldrich and were not purified any further. Each reaction was monitored by the use of thin layer chromatography (TLC) which was performed on Whatman silica gel aluminum backed plates (F-254). TLC was visualized with a dilute (2%) sulfuric acid stain and heat. Melting points were measured with a Thomas Hoover capillary melting point apparatus. All reagents were reagent grade unless otherwise specified.

6-Fluorocholesterol

To the established starting compound- 6-ketocholestanol (220 mg, 0.546 mmol) was acetylated by adding 7 mL of pyridine and 2.5 mL of acetic anhydride at 0 ^{o}C. The reaction mixture was stirred at room temperature for 17 hours and the resulting reaction mixture was poured over an ice/water mixture. The desired organics were extracted with ether, then combined the organic layers were dried over magnesium sulfate, filtered and concentration in vacuo to yield crude 3-acetoxycholest-6-one. 3-Acetoxycholest-6-one was then dissolved in dimethoxyethane (glyme) (7 mL) in a HDPE Teflon coated container and two drops of fuming sulfuric acid (70%) was added. Diethylaminosulfurtriflouride (DAST) (0.5 mL) was added to the reaction mixture and the reaction was heated at 50°C for 21 days under an atmosphere of nitrogen. The reaction mixture was carefully poured into a 5% sodium bicarbonate solution with ice. The desired organics were extracted with ether, the combined organic layers were dried over magnesium sulfate, concentrated in vacuum to yield crude 3-acetoxy-6-fluorocholesterol. The crude material was purified by column chromatography on silica gel eluting with 6-25% ether: petroleum ether to yield pure 3-acetoxy-6-fluorocholesterol (mp 100°C; lit. M.P. ~ 95°-100°C).[1] 3-Acetoxy-6-fluorocholesterol was immediately deacetylated by mixing with 10% potassium hydroxide in methanol (4mL). The reaction mixture was then extracted with ether (x3) and the organic layers

were dried with magnesium sulfate and concentrated in vacuo to yield 6-fluorocholesterol as a colorless solid (52.4 mg, 24% yield over three steps). EI-MS: m/z M^+ for $C_{27}H_{45}FO$ calculated 404.3; observed 404.3. ^{1}H NMR values for 6-FCH matched the literature.[2] ^{13}C (125 MHz, $CDCl_3$): δ 152.0 (J_{CF} = 252 Hz), 116.2 (J_{CF} = 8.0 Hz), 70.6, 56.4, 56.4, 56.0, 49.8, 42.3, 39.5, 37.6, 36.1, 36.1, 35.7, 32.2, 32.0, 31.8, 28.1, 28.0, 24.2, 23.8, 22.8, 22.6, 21.1, 19.3, 19.3, 18.7, 11.8

Fresh cholest-4-ene-3-one

To cholesterol (2.0 g, 5.17 mmol) was added toluene (60 mL), a solution of purified aluminum isopropoxide and dry toluene (0.33 g/mL) (37 mL) and cyclohexanone (25 mL). This reaction mixture was heated at 130°C for 1 hour and 40 minutes. The reaction mixture was then cooled to room temperature and diluted with toluene. The mixture was then washed with sodium potassium tartate, dried over $MgSO_4$ and the organics were extracted with ether. The combined organic extracts were then concentrated in vacuo and the crude material was purified by column chromatography using silica gel as the stationary phase and an eluting gradient of 0-18% ether:petroleum ether to yield cholest-4-3-one (1.91 g 96%).

Cholest-4,7-diene-3-one

To 7-dehydrocholesterol (1.2 g, 3.12 mmol) was added toluene (25 mL), a solution of purified aluminum isopropoxide and dry toluene (0.33 g/mL) (22 mL) and cyclohexanone (60 mL). This reaction mixture was heated at 130°C for 50 minutes. The reaction mixture was then cooled to room temperature and diluted with toluene. The mixture was then washed with sodium potassium tartate and was extracted with ether. The combined organic extracts were then concentrated in vacuo and purified by column chromatography using silic gel as the stationary phase and and an eluting gradient of 0-20% ether:petroleum ether to yield cholest-4,7-diene-3-one (0.46 g, with trace cholesta-5,7-dien-3-one).

Fresh cholestanol

Cholesterol (0.580 g, 1.50 mmol) was dissolved in ethyl acetate (EtOAc)and a pinch of 10% Pd/C was added. The reaction mixture was evacuated under vacuum and replenished with an atmosphere of hydrogen gas (X3). The reaction mixture was allowed to stir overnight at room temperature. The reaction mixture was then diluted with EtOAc and filtered through a bed of celite. The filtrate was then evacuated in vacuo to yield crude material that was used for the next step.

Fresh cholestanone

Crude cholestanol (assume 100% from previous step), PCC (0.810 g, 3.76 mmol) and celite (0.4 g) were suspended in dichloromethane (DCM) and the reaction mixture was allowed to stir at room temperature for 3 h under an atmosphere of nitrogen. The resulting mixture was then filtered through a short bed of celite and the crude was dry loaded onto silica and purified by

column chromatography using silica gel as the stationary phase and an eluting gradient of 9-20% ether:petroleum ether to yield cholestanone as a colorless solid (432 mg, 75% for two steps).

2α-Methyl-5α-cholestan-3-one, 2β-methyl-5α-cholestan-3-one and 4α-methyl-5α-cholestan-3-one and 4β-methyl-5α-cholestan-3-one

Cholestanone (432 mg, 1.12 mmol) was dissolved in dry THF (8 mL) and the reaction mixture was cooled to -78°C. LDA (1.0 M in THF) was added dropwise to the reaction mixture and the resulting reaction mixture was allowed to stir at -78°C for 50 min under an atmosphere of nitrogen. HMPA (0.6 mL) was then added dropwise to the reaction mixture. A solution of methyl iodide (0.80 g, 5.6 mmol) in 3 mL dry THF was then added dropwise to the previously mentioned reaction mixture at -78°C and the resulting reaction mixture was allowed to stir at -78°C for 2.5 h under an atmosphere of nitrogen. To the reaction mixture was carefully added 10% citric acid and the reaction mixture was allowed to stir 20 minutes, while warming up to room temperature. The organics were then extract with ether, washed with 0.5% $NaHCO_3$, water, brine and then dried over $MgSO_4$. The crude was then dry loaded onto silica and purified by column chromatography using silica gel as the stationary phase and an eluting gradient of 3-5 % ether:petroleum ether to yield a mixture of the four 2- and 4-methyl sterol isomers as a colorless solid (240 mg, 54%), purified by reversed phase HPLC eluted with methanol.

References.

1. Boswell, G.A. 1978. Preparation of Vinylene Fluorides. U.S. Patent 922,048.
2. Harte, R.A., S. J. Yeaman, J. McElhinney, C. J. Suckling, B. Jackson, B, and K. E. Suckling, 1996. The synthesis of A- and B-ring fluorinated analogues of cholesterol. *Chem. Phys. Lipids* **83**: 45-59.

Supplementary Figure 10. Sterol profiling the metabolite composition of *C. elegans* fed with 16 sterols. The results are for one set of sterol supplementation to nematodes as described in Materials and Methods. Minimal variation (no greater than 5% variation for any sterol peak in GC-MS analysis) was detected in the experimentally determined sterol profiles when the experiment was repeated.

Supplementary Figure 11. Partial set of sterol structures examined in this study (See Supplementary Tables 1 and 2 for a key to chromatographic and mass spectral properties of these compounds). Additional structures of sterol metabolites generated in *C. elegans* are shown in Supplementary Figures 13 16.

Supplementary Figure 12. Mass spectra of sterols examined in this study. Figures 12A to 12-I show mass spectra of non-deuterated sterol supplements and metabolites while Figure 12 J shows mass spectra of deuterted sterol metabolites from a cholestanone plus [$^{2}H_3$-*methyl*]methionine feed.

Supplementary Figure 13. Metabolite profile of *C. elegans* fed campesterol (structure 45). Panel A reports the GC-MS analysis of total sterol; numbers in GC correspond to compounds reported in Panel C and illustrated in Panel B.

Supplementary Figure 14 . Mass spectra of new sterols (14A to 14C) detected in *C. elegans* fed with campesterol (See Supplementary Figure 13 Panel A).

Supplementary Figure 15. Proposed biosynthetic scheme of C4, C24-dimethyl sterols synthesized in *C. elegans* fed campesterol (24α-methyl cholesterol). Note, for this metabolic pathway additional enzymes are used from the ones in cholesterol metabolism pathway in Figure 6 (main text)- that is a C24-dealkylase yielding the $\Delta^{24(25)-}$bond in cholesta-5,7,24-trienol [**43**] and a $\Delta^{24(25)}$-reductase yielding the saturated side chain in cholesta-5,7-dienol [**2**]..

Supplementary Figure 16. Metabolite profile of *C. elegans* fed with cholesta-8,24-dienol (zymosterol) [**25**].

Supplementary Figure 17. Mass spectra derived from GC-MS analysis (Panels 17A and 17B) and UV spectra derived from HPLC-UV analysis (Panel 17C) of zymosterol substrate [**25**] showing end absorbtion for the monene structure [**40**] and λmax of 268 nm for the $\Delta^{9(11), 8(14)}$-dienol structure of metabolite [**52**] compared to the UV spectra of reference cholesta-5,7-dienol [**2**].

Supplementary Figure 18. Proposed biosynthetic scheme for metabolism of zymosterol to a C4-methyl 8(14),9(11)-dienol product. Compounds identified are reported in GC trace shown in Supplementary Figure 16 (Panel A).

Supplementary Figure 19. Conserved and divergent amino acids in 4-SMT and 24-SMT proteins. Substrate binding segments- for sterol Regions 1, 3, and 4 and for SAM Region 2- are based on 24-SMT. Stars indicate relevant conserved amino acid residues in Regions I to 4 of 4-SMT and 24-SMT that can distinguish catalytic competence. Accession number*: Ce-4-SMT: Caenorhabditis elegans* NP_497549.2; *Cb-4-SMT: Caenorhabditis brenneri,* CAP33846.2; *Ac-4-SMT: Ancylostoma ceylanicum,* EYC26550; *Hc-4-SMT: Haemonchus contortus,* CDJ90475; *Dv-4-SMT: Dictyocaulus viviparus,* KJH45819; *Ll-4-SMT: Loa loa,* XP_020303948; *Bm-4-SMT: Brugia malayi,* XP_001900512; *Sm-4-SMT: Symbiodinium microadriaticum,* OLQ09145; *Sc-24-SMT: Saccharomyces cerevisiae,* ONH72293; *At-24-SMT: Arabidopsis thaliana,* AAM53553; *Gm-24-SMT: Glycine max,* NP_001238391; *Ac-24-SMT: Acanthamoeba castellanii,* XP_004336540; *Ac-28-SMT: A. castellanii ,* XP_004335307; *Tb-24-SMT: Trypanosoma brucei,* XP_822930; *Aq-24-SMT: Amphimedon queenslandica,* XP_003387525; *Sm-24-SMT: S. microadriaticum,* OLP85101; *At-28-SMT1: A. thaliana,* AAM91592; *Gm-28-SMT: G. max,* ACS93764; *At-28-SMT2: A. thaliana,* NP_177736; *Cr-28-SMT: Chlamydomonas reinhardtii,* XP_001690775

Supplementary Figure 20. SDS-Gel of cloned *C. elegans* 4-SMT expressed in *E. coli* at different time points. Marker proteins are shown to the left.

Supplementary Figure 21. Structures of 2-methyl and 4-methyl biomarkers and naturally occurring C4-methyl sterols with nucleus of Δ^{0}, Δ^{5}, Δ^{7} and $\Delta^{8(14)}$.

For a key to the literature see: references: (i) For [**1A**] and [**2A**]- (i) Summons, R. E., and R. J. Capon. 1991. Identification and significance of 3β-ethyl steranes in sediments and petroleum. *Geochim. Cosmochim. Acta.* **55**: 2391-2395 and (b)- Aboglila, S., K. Grice, K., Trinajstic, C.

Snape, and K. H. Williford. 2011. The significance of 24-norcholestanes, 4-methylsteranes and dinosteranes in oils and source-rock from East Sirte Basin (Libya). *Appl. Geochem*. 26, 1694-1705 and For [**3A**] to [**7A**]- (iii) Elenkov, I., B., S. Dragova, S. Andreev, and A. Popov. 1997. 4α-Methyl sterols from the sponges *Haliclona cinerea* and *Halicolona flavescens*. *Comp. Biochem. Physiol. B* **118**: 155-157. (iv) Sepe, V., R., M. V. Ummarino, M.G. D'auria, G. Chini, G. Bifulco, C. Renga, C. D'Amore, S. Debitus, S. Fiorucci, and A. Zampella. 2011. Conicasterol E, a small heterodimer partner sparing farnesoid X receptor modulator endowed with a pregnane X receptor agnostic activity, from the marine sponge *Theonella swinhoei*. *J. Med. Chem*. **55**: 84-93, (v) Withers, N.W., W.C.M.C. Kokke, W. Fenical, and C. Djerassi. 1982. Sterol patterns of cultured zooanthellae isolated from marine invertebrates: Synthesis of gorgosterol and 23-desmethylgorgosterol by aposymbiotic algae. *Proc. Natl. Acad. Sci. U. S. A.* **79**: 3764-3768 and (vi) Goad, L. J. 1978. The sterols of marine invertebrates: Composition, biosynthesis and metabolites. In, Marine Natural Products. P. J. Scheuer, editor. Academic Press, New York. 75-172.

Supplementary Figure 22. Hypothetical evolution of hopanoids in red (diplopterol [**11A]** converts to 2-methyl diploterol [**13A**] by 2-methyl hopanoid transferase, 2-mHP) and 4/23/24/27- methylated sterols in blue ([**15A**] to [**28A**]) based on their corresponding hopane and sterane biomarkers detected in the fossil record and natural occurrence in biology.

As shown in this figure there is a growing understanding of the initial appearance of 2-methyl hopanes in anaerobic prokaryotes that is followed by the appearance of 24-desalkyl/24-alkyl steranes, 4-methyl-steranes and 24/26-methyl steranes in eukaryotes following the advent of atmospheric oxygen. As a working hypothesis based on our sterol evolution and sterol methylation research (see also Supplementary Figure 23) cited in this paper and appreciation of the hopane/sterane biomarker literature and Tree of Life studies, there existed a common ancestor that decented initially into multiple branches with one line to Rhizaria (Nettersheim, B. J.; J.J. Brocks, A. Schwelm, J.M. Hope, F. Not, M. Lomas, C. Schmidt, R. Schiebel, E.C. Nowack, P. De Deckker, J. Pawlowski, S. S. Bowser, I. Bobrovskiy, K. Zonneveld, M. Kucera, M. Stuhr, and C. Hallman. 2019. Putative sponge biomarkers in unicellular *Rhizaria* question an early rise of animals. *Nat. Ecol. Evol.* **3**: 577-581) and one line to the Excavates (Cavalier-Smith, T. 2006. *Phil. Trans. R. Soc.* B 361: 969-1006), together these orgamisms likely provided the crown C_{27}-C_{29}-steranes, which correlate to the C_{27}-C_{29}-sterols presumably synthesized first in the LECA.

Biomarker literature: (i) Brassell, S. C. Isopentenoids and Geochemistry. 1994. *In* Isopentoids and Other Natural Products: Evolution and Function. W. D. Nes, editor. *ACS symp. ser.* **562**: 2-30 (1994). (ii) Brocks, J.J., G.D. Love, R.E. Summons, A. H. Knoll, G. A. Logan, S. A. and Bowden, 2005. Biomarker evidence for green and purple sulphur bacteria in a stratified paleoproterozoic sea. *Nature* **437**: 866-870. (iii) Brocks, J., and R. E. Summons. 2003. Sedimentary hydrocarbons, biomarkers for early life. *Treatise on Geochemistry* **8**: 682-695. (iv) Brocks, J. J., A. J. Jarrett, E. Sirantoine, F. Kenig, M. Moczydlowska, S. Porter, and J. Hope. 2016. Early sponges and toxic protists: possible sources of cryostane, an age of diagnostic biomarker antedating sturtian snowball earth. *Geobiology* 14, 129-149. (v) Zumberge, J. A., G. D.

Love, P. Cardenas, E. A. Sperling, S. Gunasekera, M. Rohrssen, E. Grosjean, J. P. Grotzinger, and R. E. Summons, 2018. Demosponge steroid biomarker 26-methylstigmastane provides evidence for neoproterozoic animals. *Nat. Ecol. Evol.* https://doi.org/10.1038/s41559-018-0676-2 .(vi) Bobrovskiy, I., J. M. Hope, A. Ovantsov, J. N. Benjamin, C. Hallmann, and J. J. Brocks, J. J. 2018. Ancient steroids establish the Ediacaran fossil Dicksonia as one of the earliest animals. *Science* **361**, 1246-1249.

Supplementary Figure 23. Hypothetical step-wise eukaryote evolution of sterol side chain and nucleus methylated compounds through the action of minimally five families of sterol methyltransferase enzymes (SMT) involved in the shortening, lengthening or broadening of sterol side chain or nucleus structures at the C24- (=24-SMT; SMT1), C28- (28-SMT; SMT2), C27- (27-SMT; SMT3, which is not to be confused with previous nomenclature for SMT3 as an isoform of SMT2)), C23-(23-SMT; SMT4) or C4-(4-SMT; SMT5)- position in accordance with the natural distribution of sterols and substrate properties of microsomal or cloned SMT enzymes identified thus far as SMT1 and SMT2; LAS= lanosterol synthase/ CAS = cycloartenol synthase. 2-mHP = 2-Methyl hopanoid (based on the pentacylic triterpenoid diplopterol skeleton).

Explanation: The original definition of SMT1 and SMT2 is based on the seminal paper by Benveniste (Bouvier-Navé, P., T. Husselstein, and P. Benveniste. 1998. Two families of sterol methyltransferase are involved in the first and second methylation steps of plant sterol biosynthesis. *Eur. J. Biochem.* **256**: 86-96) in which two distinct SMT enzyme types were described based on differences in their amino acid sequences that correlated to the substrate properties of the cloned enzymes shown to catalyze the first (SMT1- cycloartenol substrate) and second (SMT2-24(28)-methylene lophenol substrate) methylations of the Δ^{24}-bond, respectively. It is important for evolutionary considerations that thus far, the two SMT families of SMT1 and SMT2 identified in Land plants first provide the foundation for all PHYLOGENOMIC/PHYLOGENETIC papers dealing with sterol methyltransferases generally.

In our model, the crown SMT is a 24-SMT (=SMT1) which accepts, and perhaps templates for recogntion, the protosterol structure of cyclortenol (cyclortenol can precede lanosterol based on thermodynamic reasoning that cyclization to cycloproyl sterol is a more higher energy route than to Δ^{8}-formation, yielding the more primitive substrate) with a $\Delta^{24(25)}$ -double bond. Intriguingly, this enzyme could be bi-functional in substrate acceptability as in the *Chlamydomonas reinhardtii* SMT (phylogenomics indicate SMT2) capable of productive binding either cycloartenol (favored substrate) or 24(28)-methylene lophenol, but not other potential substrates that contain, for instance the $\Delta^{25(27)}$ –bond. (Haubrich, B. A., E. K. Collins, A. L. Howard, Q. Wang, W. J. Snell, M. B. Miller, C. D. Thomas, S. K. Pleasant, and W. D. Nes. 2015. Characterization, mutagenesis and mechanistic analysis of an ancient algal sterol C24-methyltransferase: Implications for understanding sterol evolution in the green lineage. *Phytochemistry* **113**: 64-72. Consequently, *Cr*SMT cannot generate complex sterol side chains typical of Porifera.

As noted in the figure, distinct SMT isoforms evolved to catalyze the formation of compounds [**17A**] and [**18A**] via 24-SMT (= SMT1), compound [**29A**] via a C27-SMT (=SMT-3 redunant isoform), compound [**30A**] via a C27-SMT promiscuous acting with 28-SMT character (=SMT-3

redundant isoform), compounds [**19A**] and [**20A**] from C28-SMT (=SMT-2), compound [**27A**] via a C23, or compounds [**26A**], [**27A**] (= SMT-4) and [**28A**] via a C4-SMT (= SMT-5) or compound [**21A**] via a dysfunctional SMT1 or SMT2 previously identified as 24-SMT above but this enzyme recognizes SAM (to add methyl) and/or SAH (to remove methyl) depending on whether methyl is removed from a C_{27} or C_{28} methylene sterol as suggested in Nes, W. R., and M. L. McKean. 1977. Biochemistry of Steroids and Other Isopentenoids. University Park Press, Baltimore 690 pp. and supported in Giner, J-L. 1993. Biosynthesis of marine sterol side chains. *Chem. Rev*. **93**: 1735-1752. On the other hand, contrasting speculation on sterol dealkylation not consistent with generation of shortened sterol side chains is Kerr, R. G., S. I. Kerr, S. Malik, and C. Djerassi. 1992. Biosynthetic studies of marine sterols: 38. Mechanism and scope of sterol side chain dealkylation in sponges: Evidence for concurrent alkylation and dealkylation. *J. Amer. Chem. Soc*. **114**: 299-303.

Two mechanisms for the successive methylation of the sterol side chain can occur: One is based on the view the land plant SMT2 and its surrogates can be substrate promiscuous as in less-advanced organisms giving rise to compounds [**17A**] and [**18A**]. The other is based on our work of product specificity determined by partitioning-explicit outcomes to $\Delta^{24(28)}$ - $\Delta^{24(25)}$ -or $\Delta^{25(27)}$ –olefin. Although, we have characterized a "SMT1" from protozoa that generates the $\Delta^{25(27)}$ -sterol chain capable of C27-methyl extension, further C27-sterol side chain modification was not possible due to regio-specificity in the sterol methylation reaction. Thus, the complex side chains of dinoflagellates and marine invertebrate Porifera necessitate a distinct SMT with an evolved active site of appropriate volume to accept SAM additions that can elongate the side chain at C27.

For a key to sterol methylation mechanisms see- (i) Lee, A.K., A. B. Banta, J-H Wei, D. J. Kiemle, J. Feng, L J Giner and P. V. Welander. 2018. C4- Sterol demethylation enzymes distinguish bacterial and eukaryotic sterol synthesis. *Proc. Natl. Acad. Sci.* **115**: 5884-5889. (ii) Stoilov, I. L, J. E. Thompson, J-H. Cho, and C. Djerassi. 1986. Biosynthetic studies of marine lipids. 9 Stereochemical aspects and hydrogen migrations in the biosynthesis of the triply alkylated side chain of the sponge sterol strongylosterol. *J. Amer. Chem. Soc*. **108**: 8235-8241. (iii) Nes, W. D. 2003. Enzyme mechanisms for sterol *C*-Methylations. *Phytochemistry* **64**: 75-95. and (iv) Nes, W. R. and M. L. McKean. 1977. Biochemistry of Steroids and Other Isopentenoids. University Park Press, Baltimore 690 pp.

Relevant W. D. Nes work is as follows:

<u>Substrate and reaction regio-specificity for Land Plant SMT1</u>: Nes, W. D., G. G. Janssen, and A. Bergenstrahle. 1991. Structural requirements for transformation of substrates by the (S)-adenosyl -L-methionine: Δ24(25)-sterol methyl transferase. *J. Biol. Chem.* **266**, 15202-15212.

<u>Multiple SMT products distinct from classical Land Plant SMT products</u>: Zhou, W., G. I. Lepesheva, M. R. Waterman, and W. D. Nes 2006. Mechanistic analysis of a multiple product sterol methyltransferase from *Trypanosoma brucei* implicated in ergosterol biosynthesis. *J. Biol. Chem.* **281**: 6290-6296.

<u>Bi-functional (distinct from promiscuous) algae 28-SMT (SMT2) as the evolutionary precursor of more substrate and reaction specific SMTs in Land Plants</u>: Haubrich, B. A., E. K. Collins, A.

L. Howard, Q. Wang, W. J. Snell, M. B. Miller, C. D. Thomas, S. K. Pleasant, and W. D. Nes. 2015. Characterization, mutagenesis and mechanistic analysis of an ancient algal sterol C24-methyltransferase: Implications for understanding sterol evolution in the green lineage. *Phytochemistry* **113**: 64-72.

Land Plant SMT1 and SMT2 isoforms have substrate preference: (i) Nes, W. D., Z. Song, A. L. Dennis, W. Zhou, J. Nam, and M. B. Miller. 2003. Biosynthesis of phytosterols: Kinetic mechanism for the enzymatic C-methylation of sterols. *J. Biol. Chem.* **278**: 34505-34516 (2003). and (ii) Neelakandan, A. K., Z. Song, J. Wang, M. H. Richards, X. Wu, B. Valliyodan, H. T. Nguyen, and W. D. Nes. 2009. Cloning, functional expression, and phylogenetic analysis of plant 24C-methyltransferases involved in sitosterol biosynthesis. *Phytochemistry,* **70**: 1982-1998.

Sterol methylation reaction channeling as a determinant of phytosterol evolution: Miller, M. B., B. A. Haubrich, Q. Wang, W. J. Snell, and W. D. Nes. 2012. Evolutionarily Conserved $\Delta^{25(27)}$-Ergosterol Biosynthesis Pathway in the Alga *Chlamydomonas reinhardtii* is Distinct from the $\Delta^{24(28)}$-Pathway to Fungal Ergosterol. *J. Lipid Res.* **53**: 1636-1645.

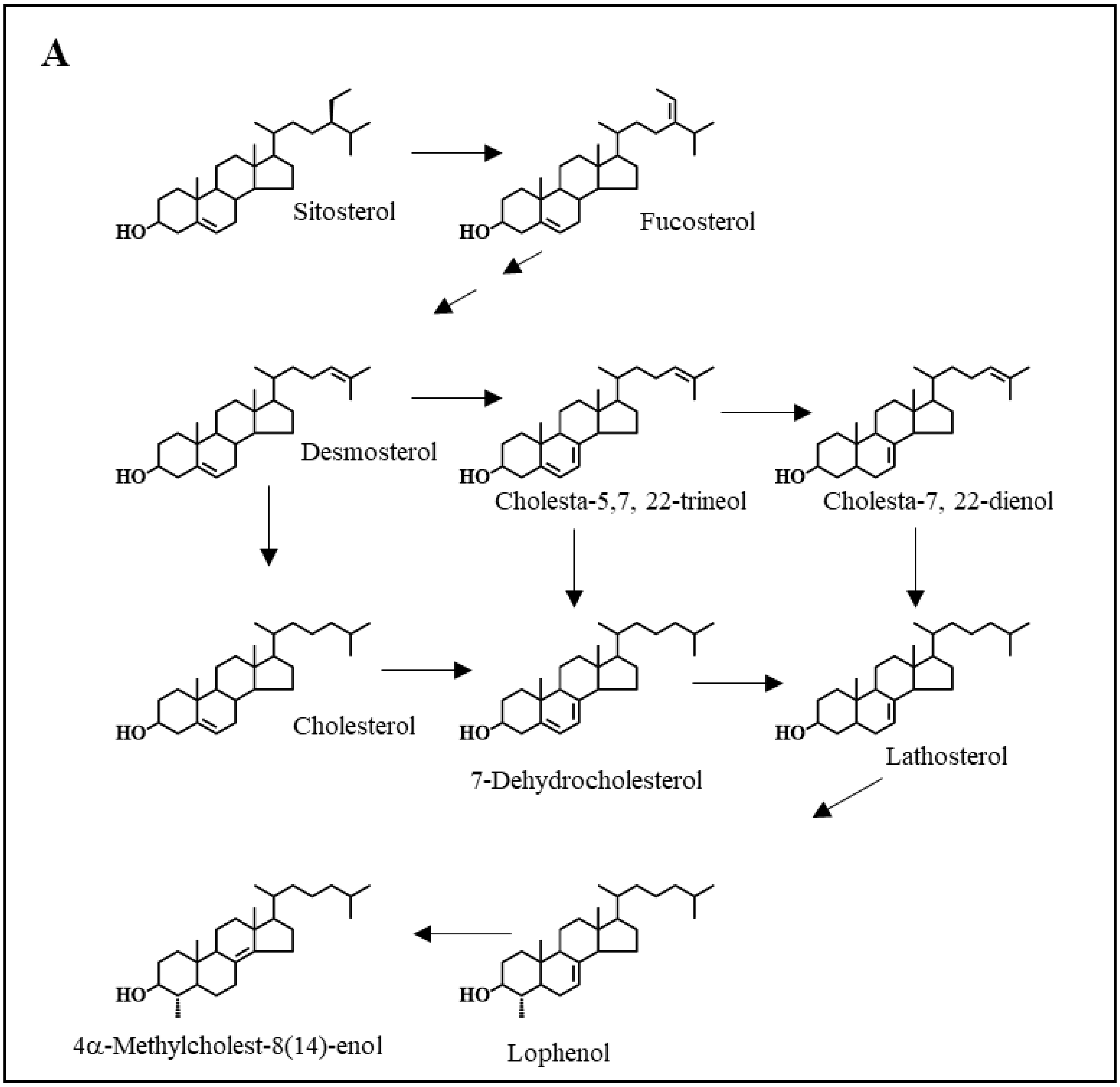

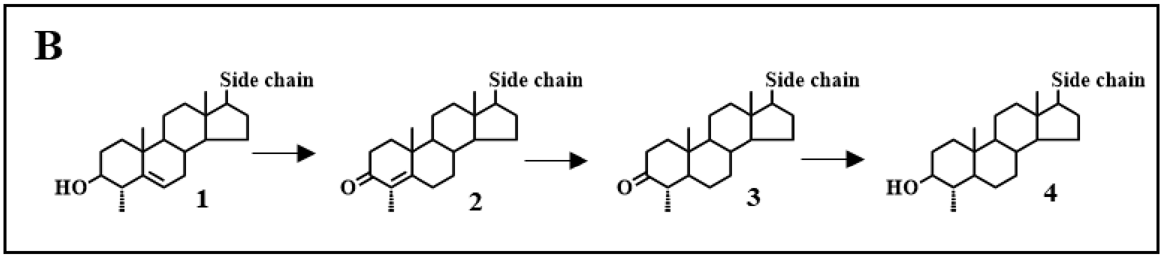

Supplementary Figure 1

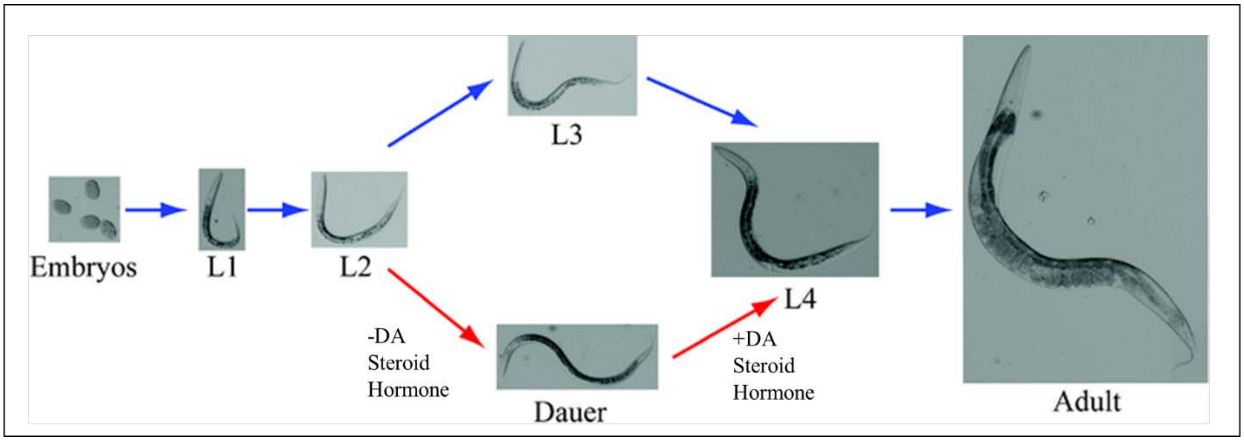

Supplementary Figure 2

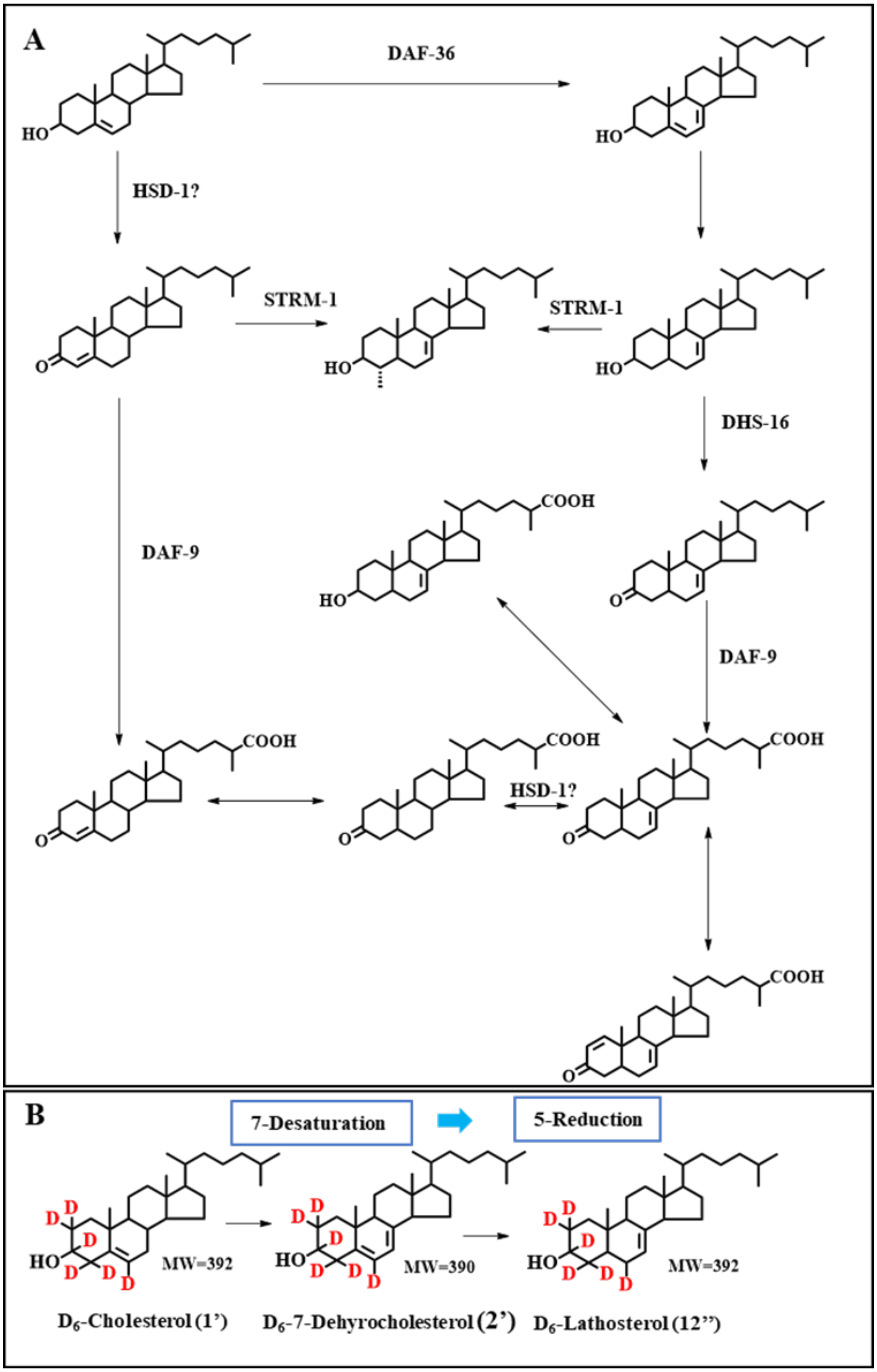

Supplementary Figure 3

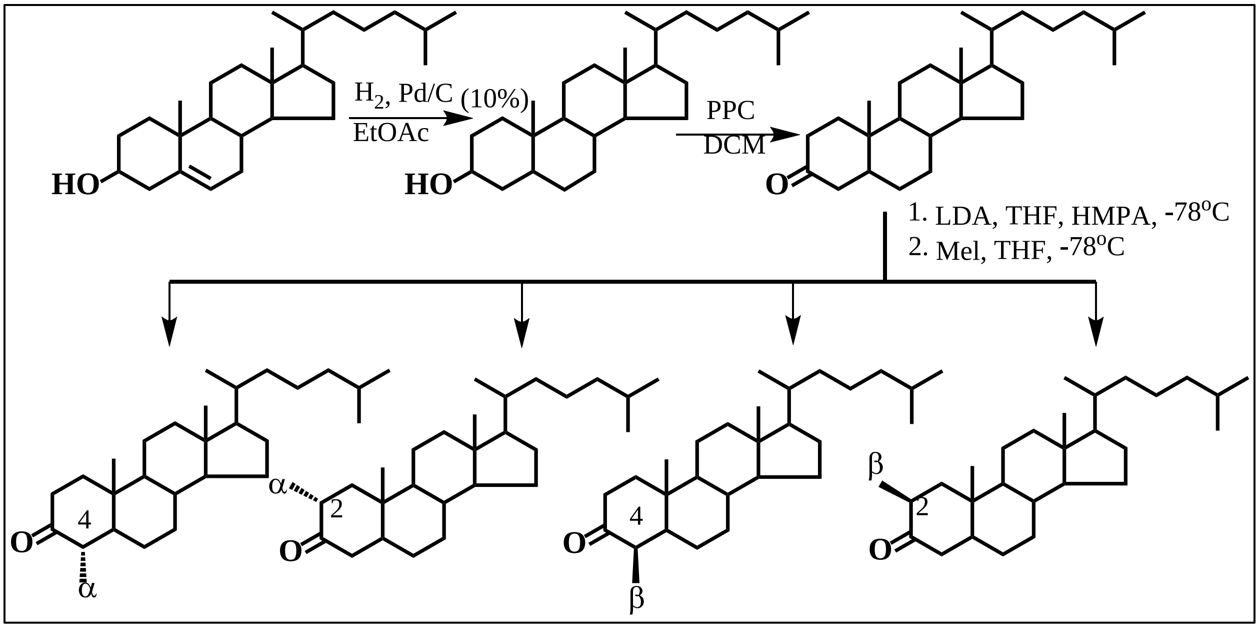

Supplementary Figure 4

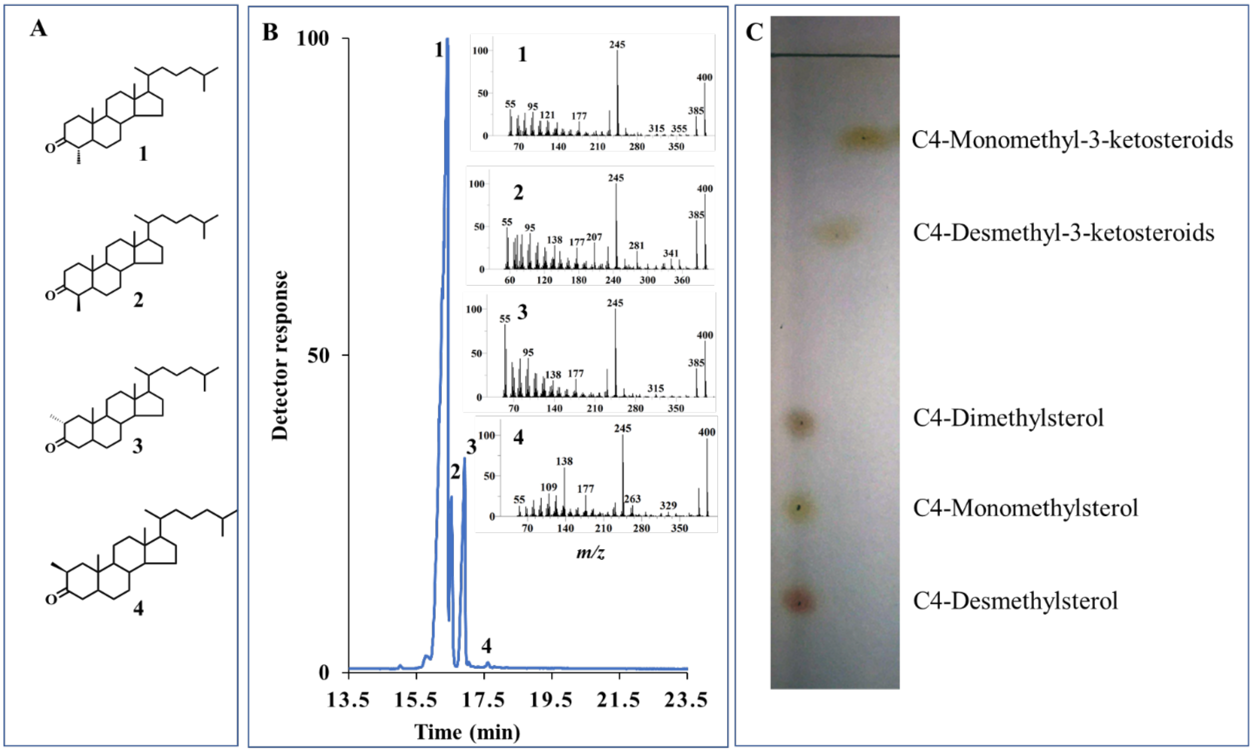

Supplementary Figure 5

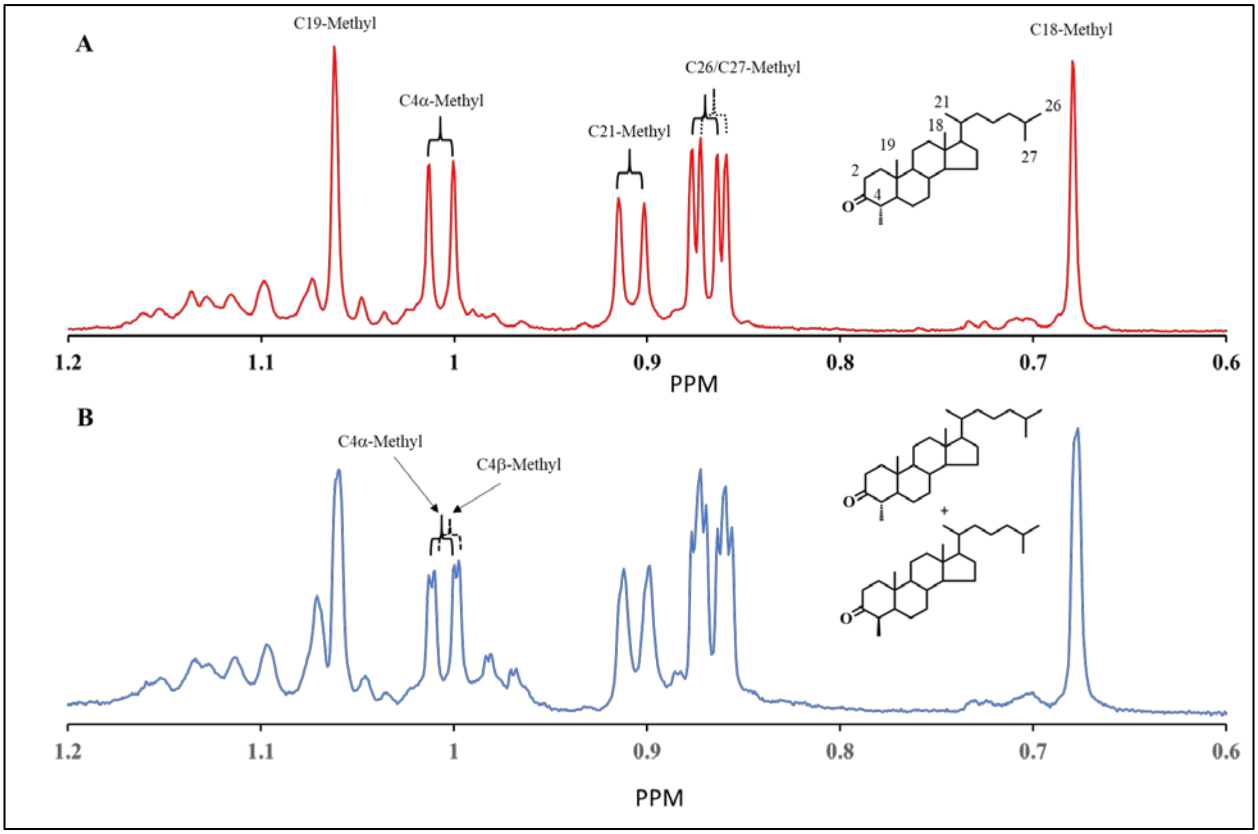

Supplementary Figures 6 (A)

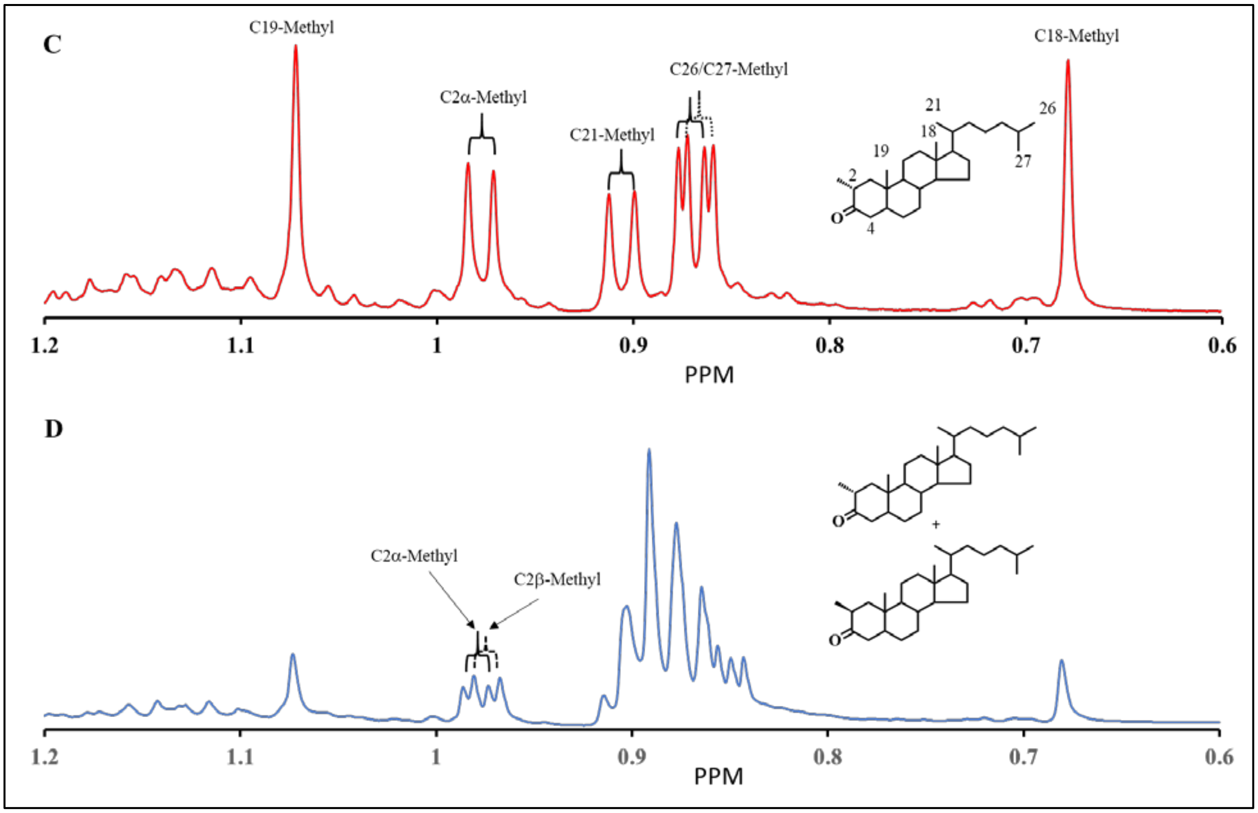

Supplementary Figure 6 (B)

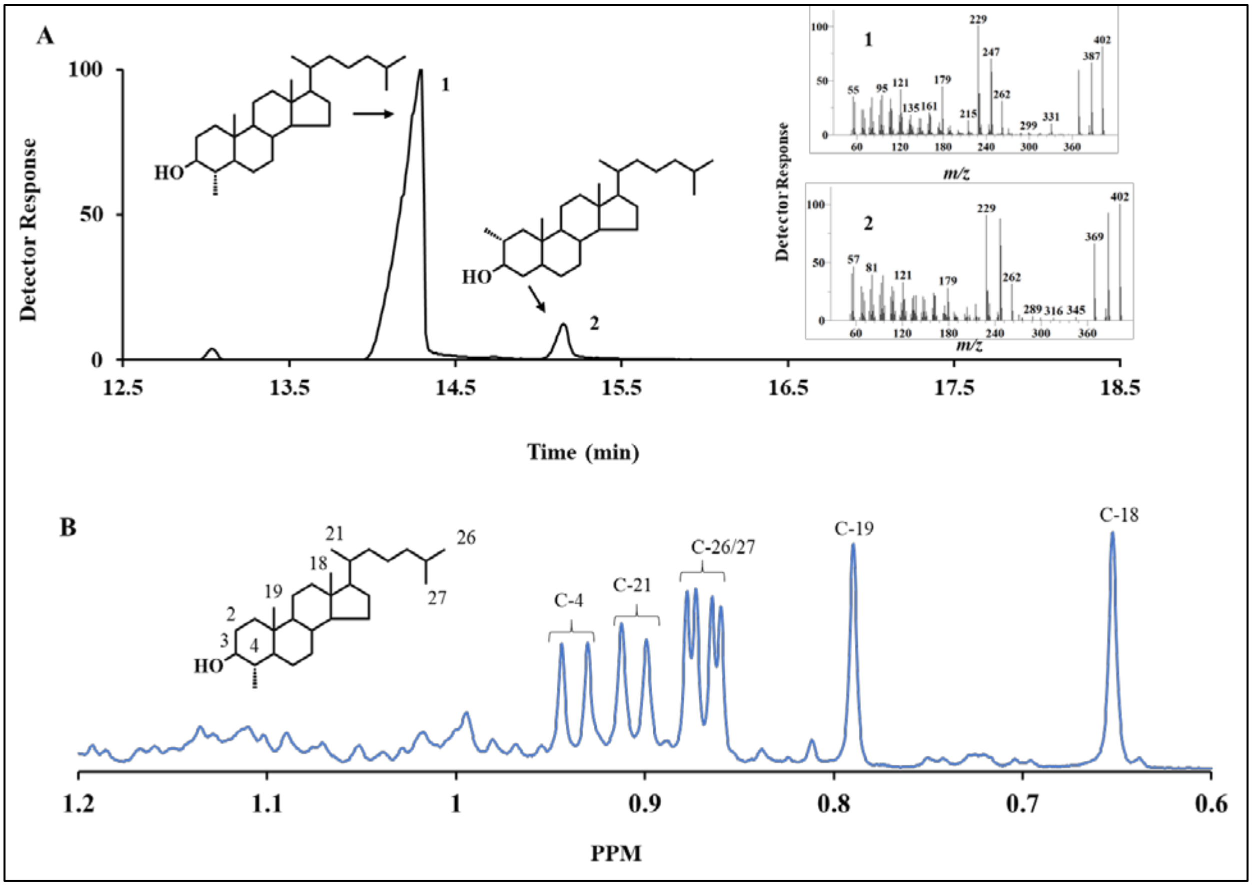

Supplementary Figure 7

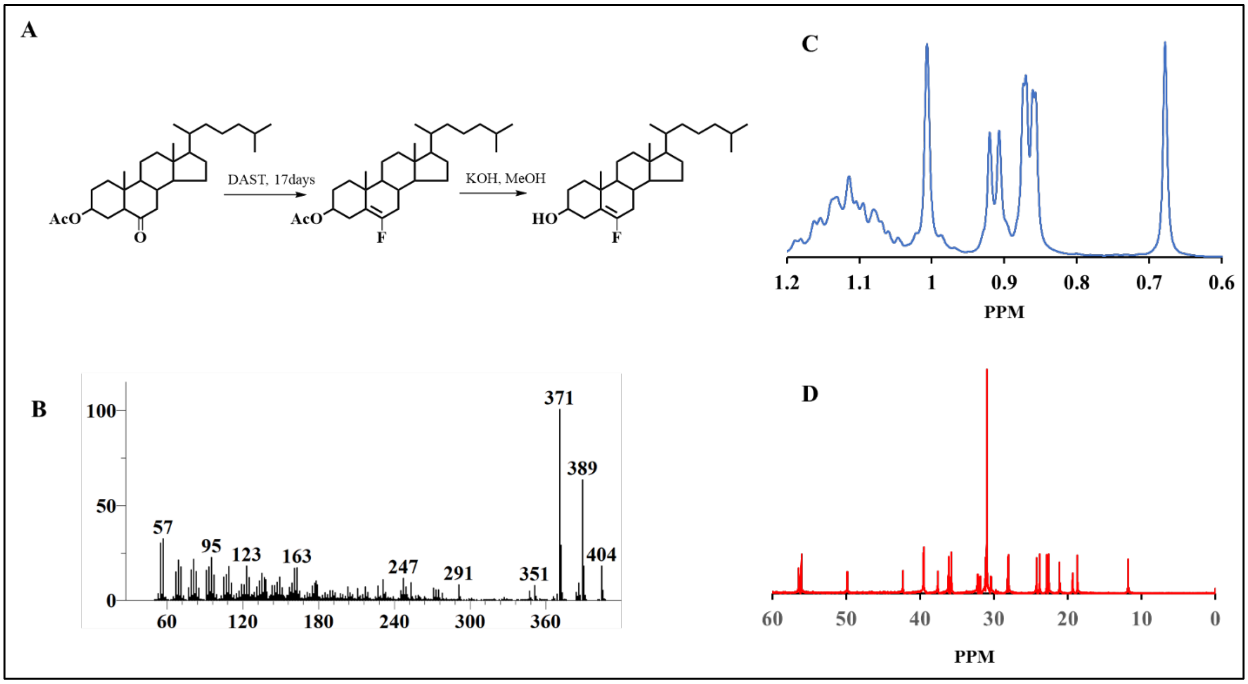

Supplementary Figure 8

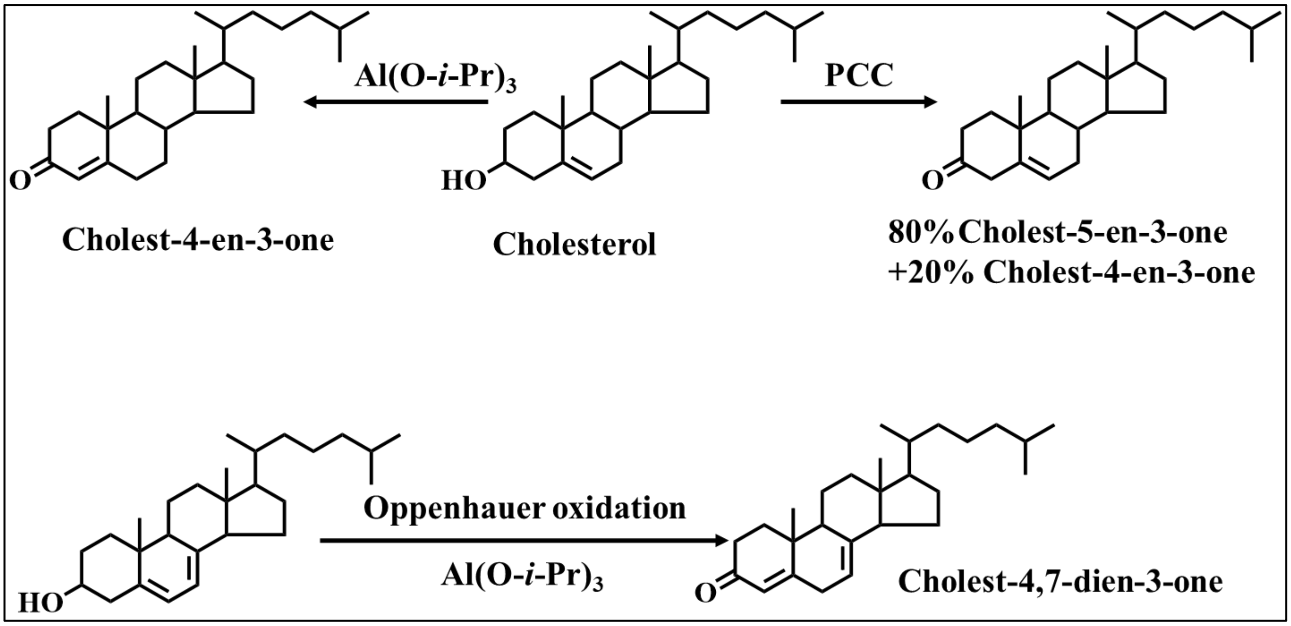

Supplementary Figure 9

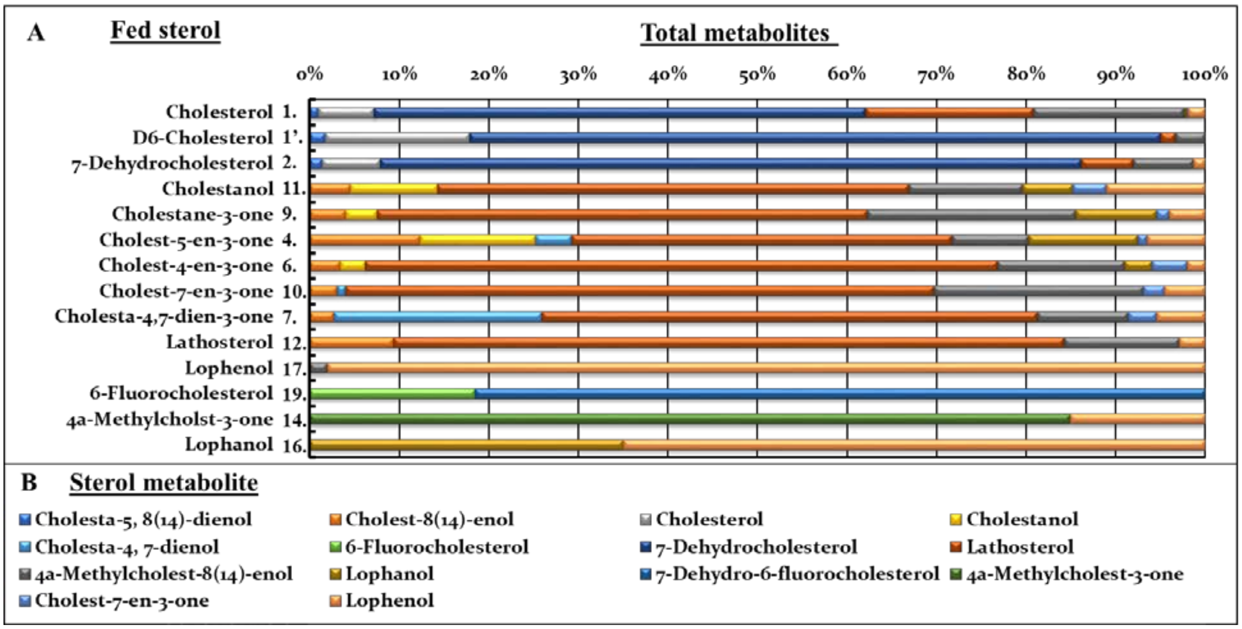

Supplementary Figure 10.

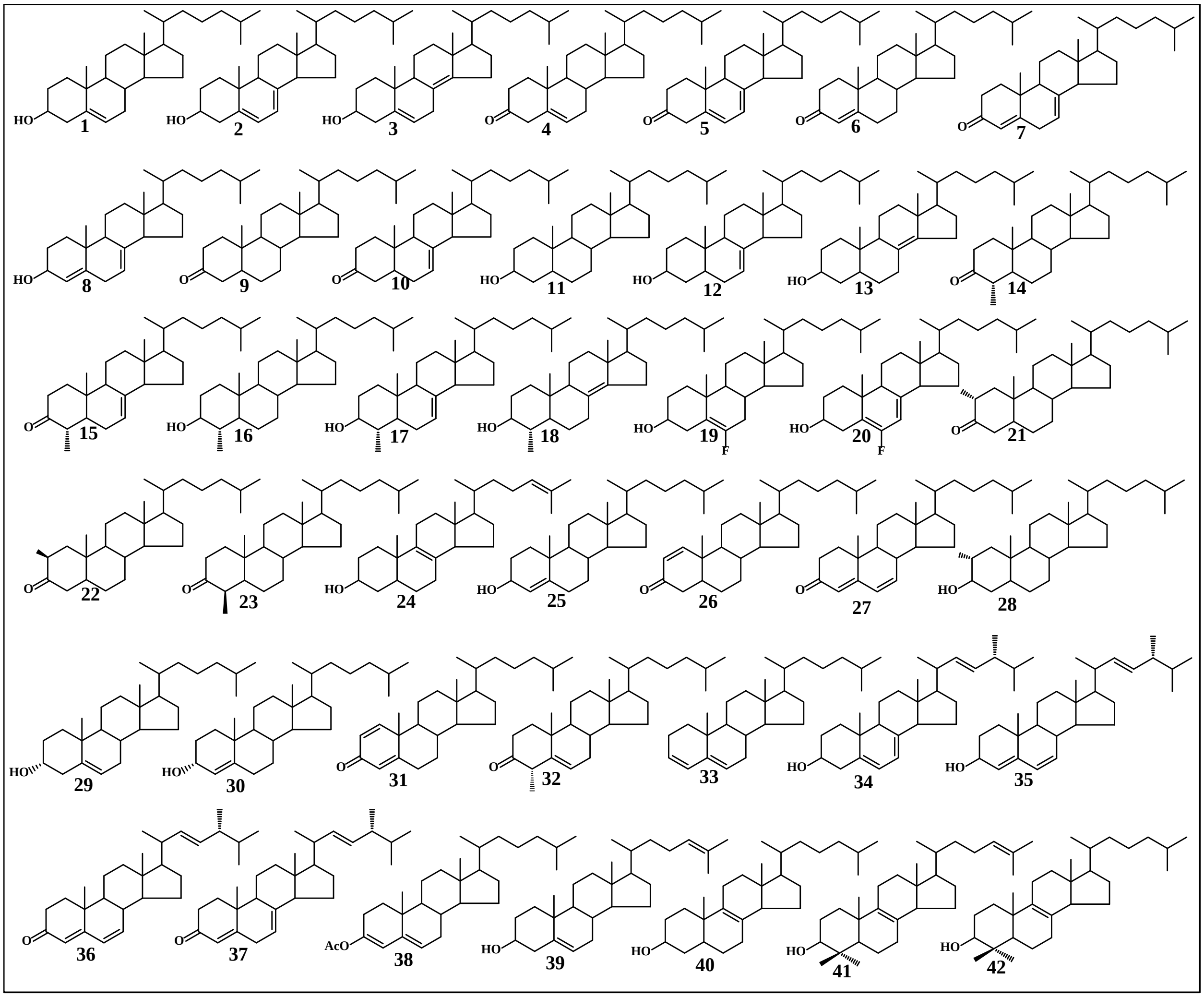

Supplementary Figure 11.

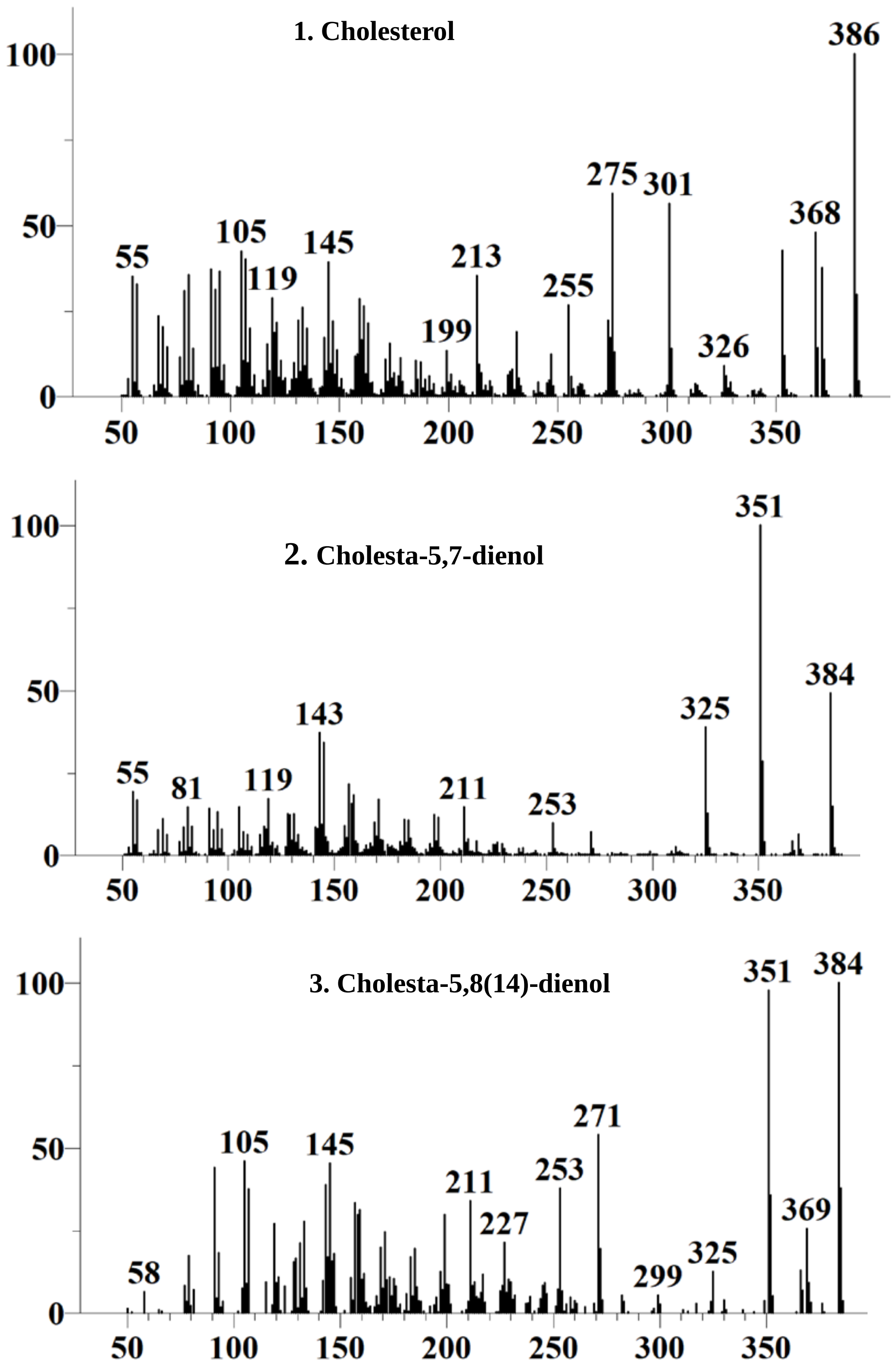

Supplementary Figure 12(A)

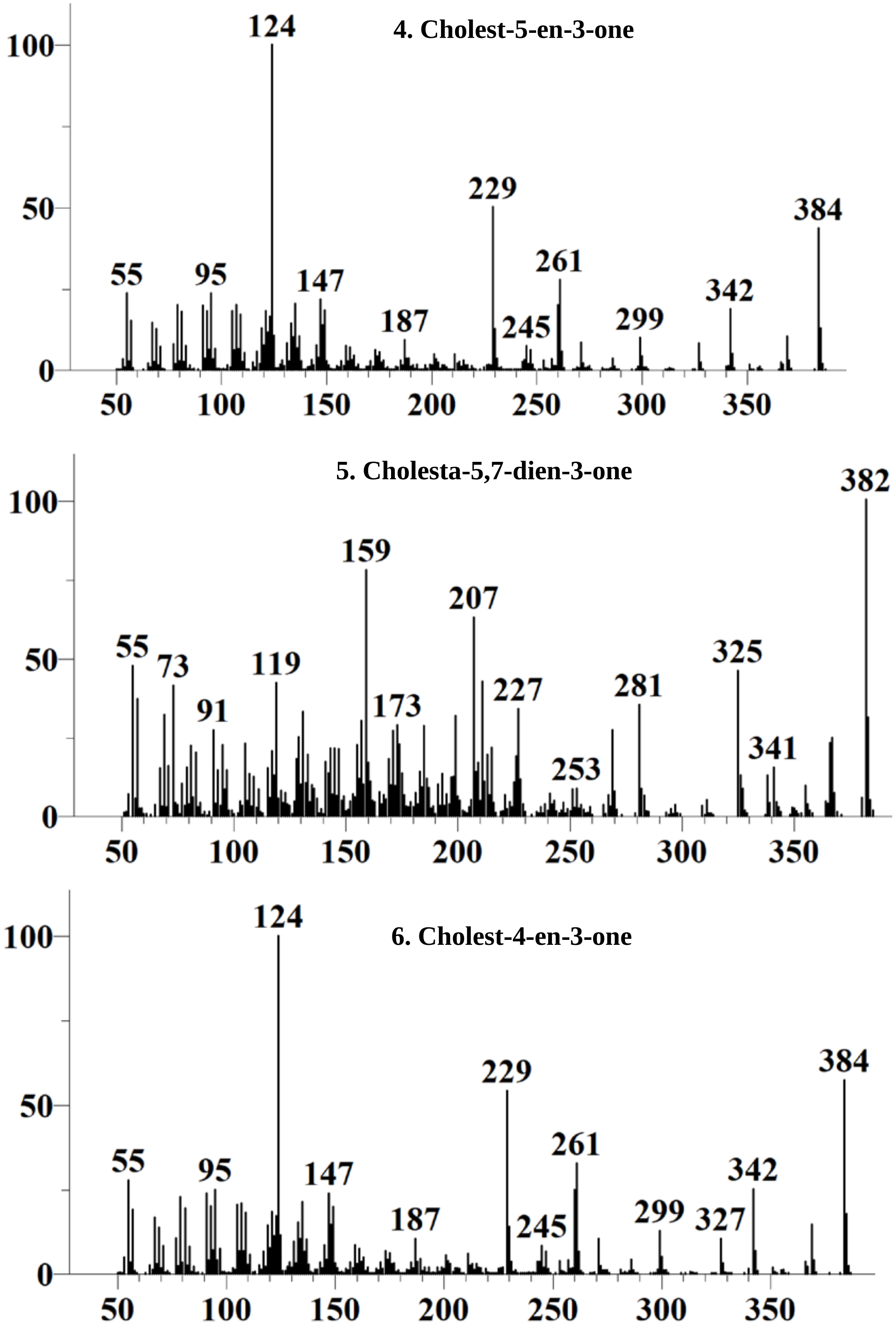

Supplementary Figure 12(B)

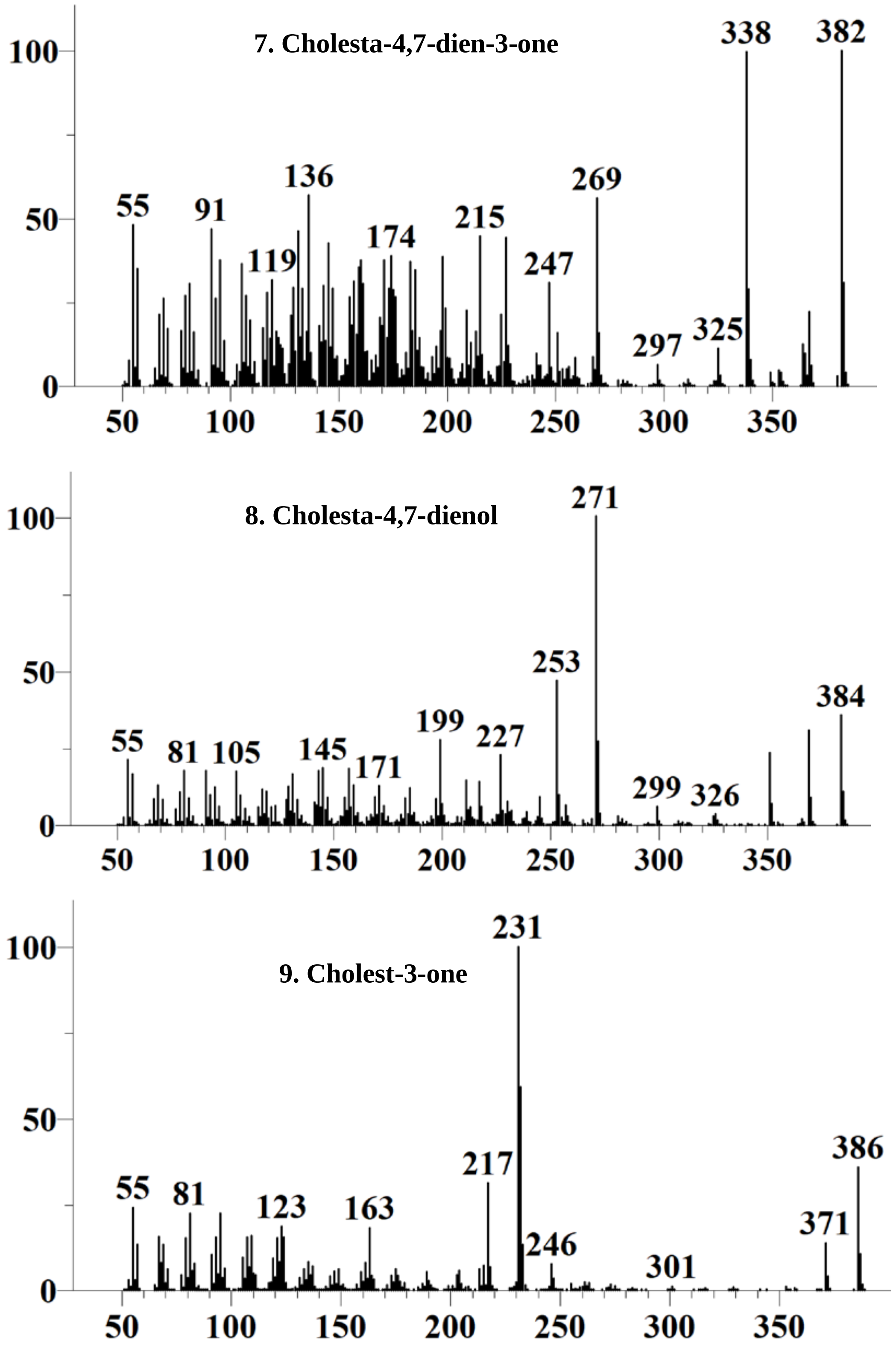

Supplementary Figure 12(C)

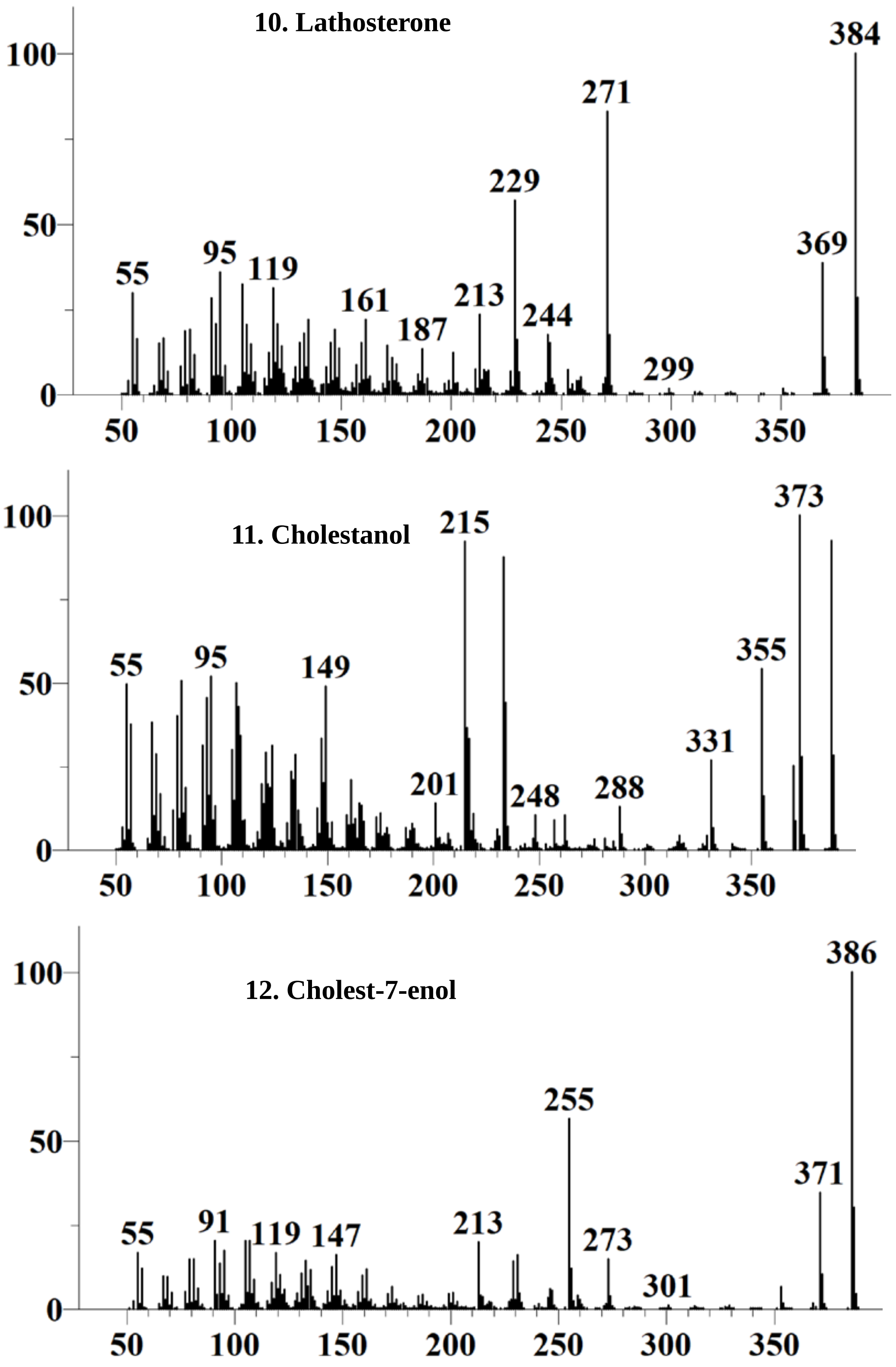

Supplementary Figure 12 (D)

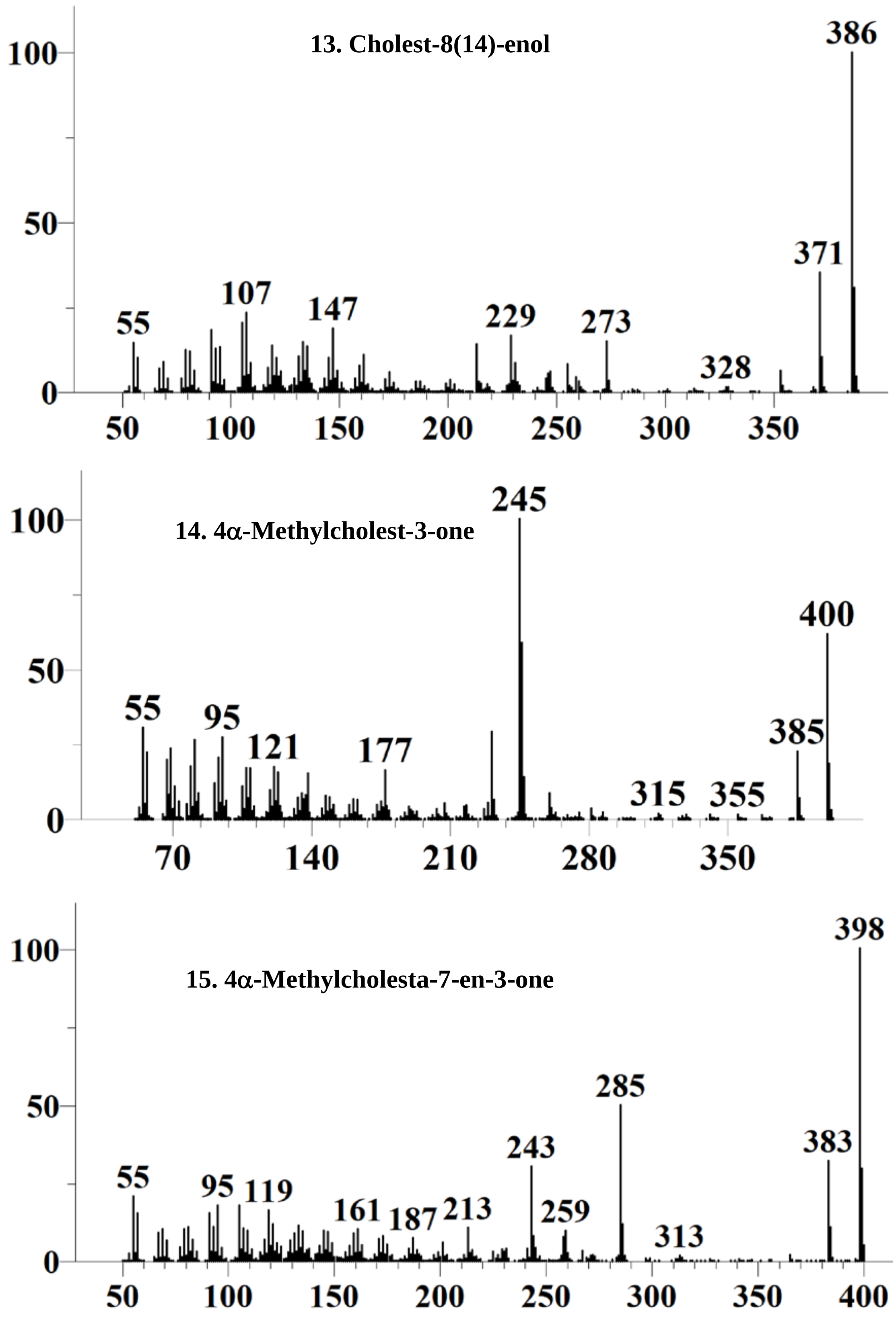

Supplementary Figure 12 (E)

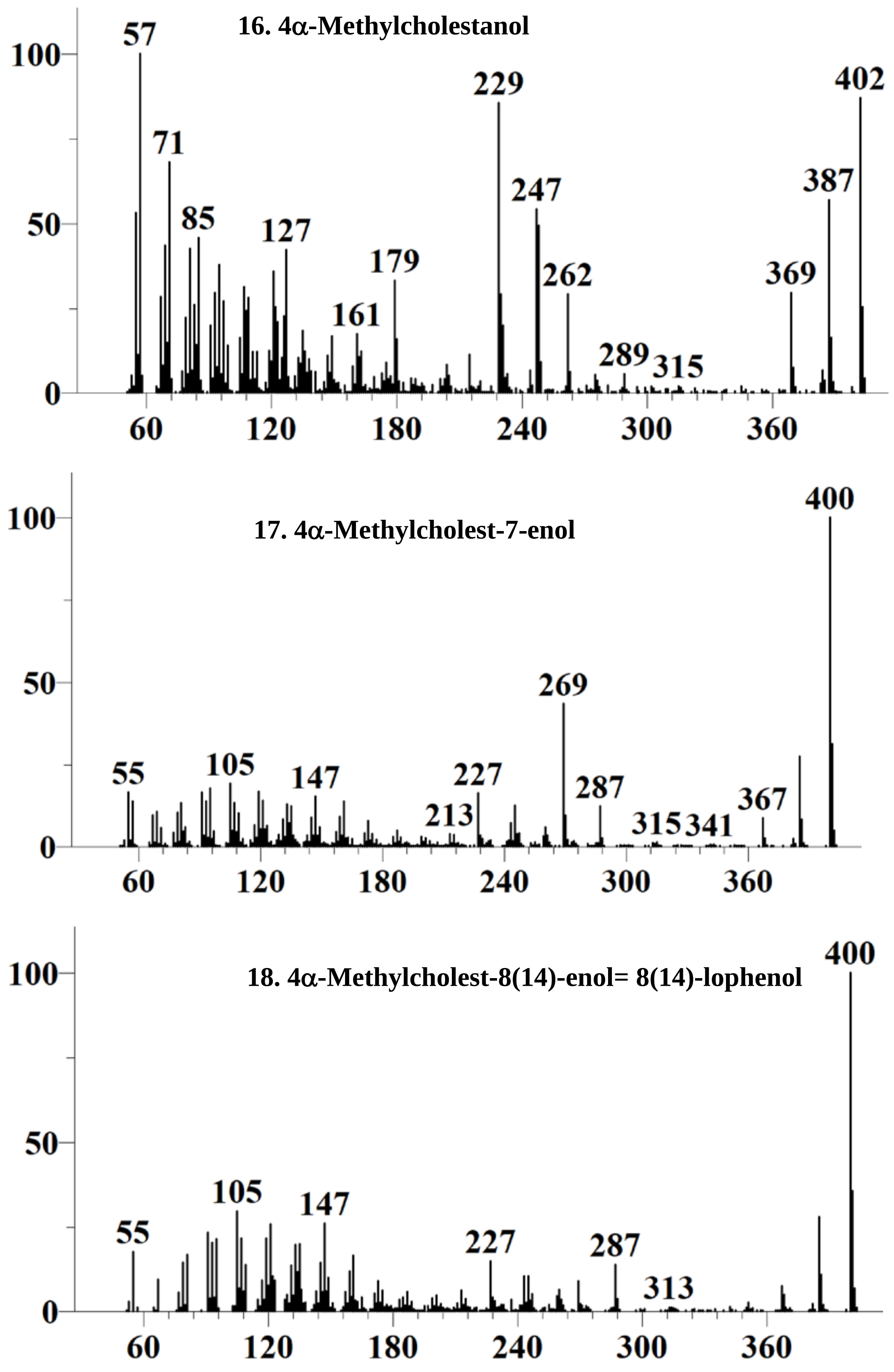

Supplementary Figure 12 (F)

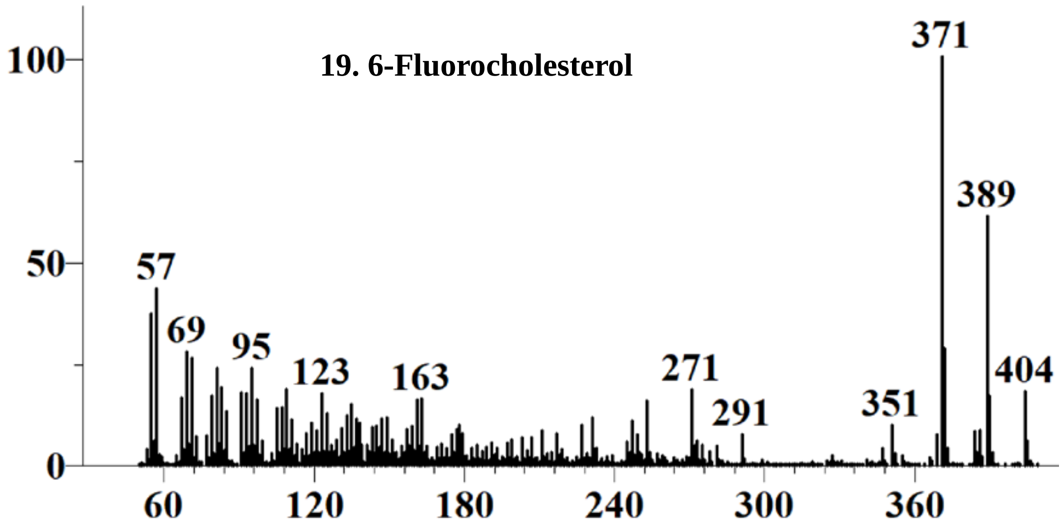

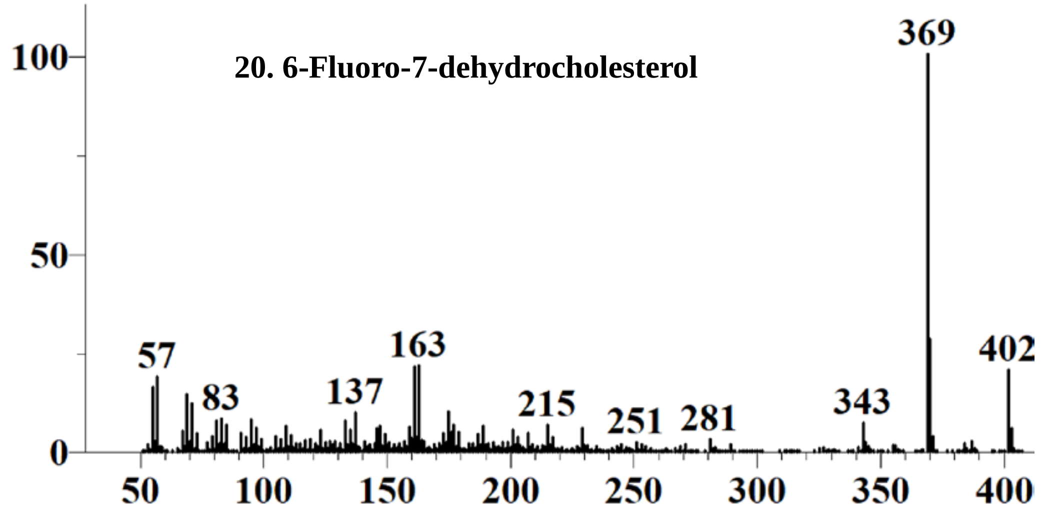

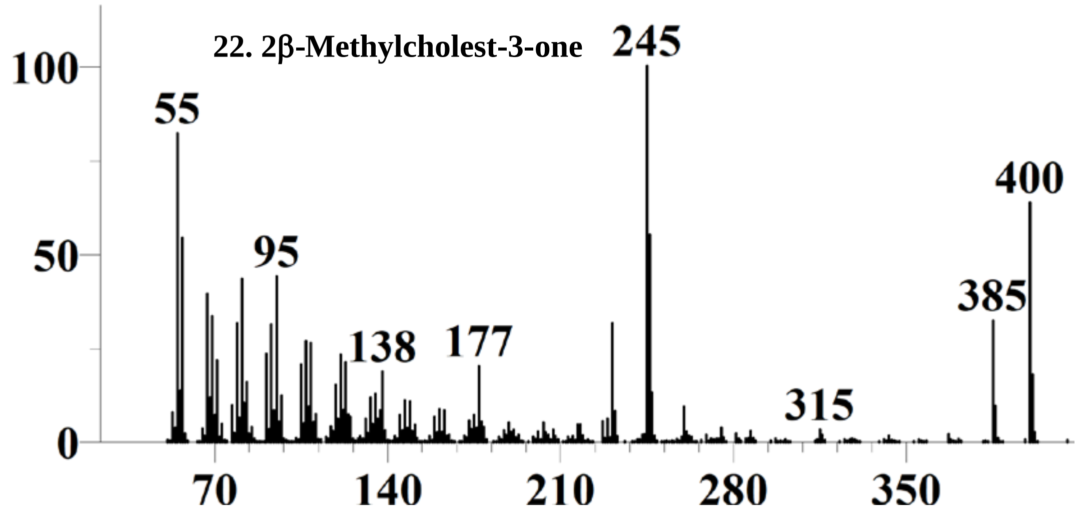

Supplementary Figure 12 (G)

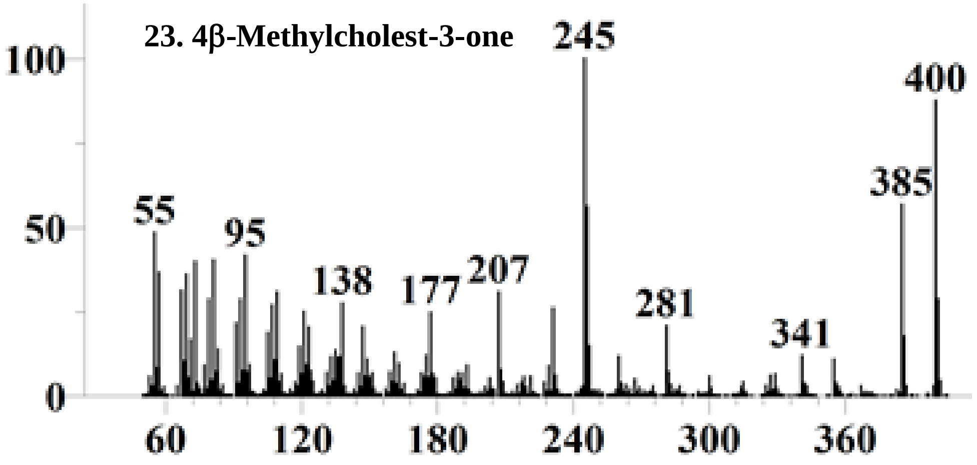

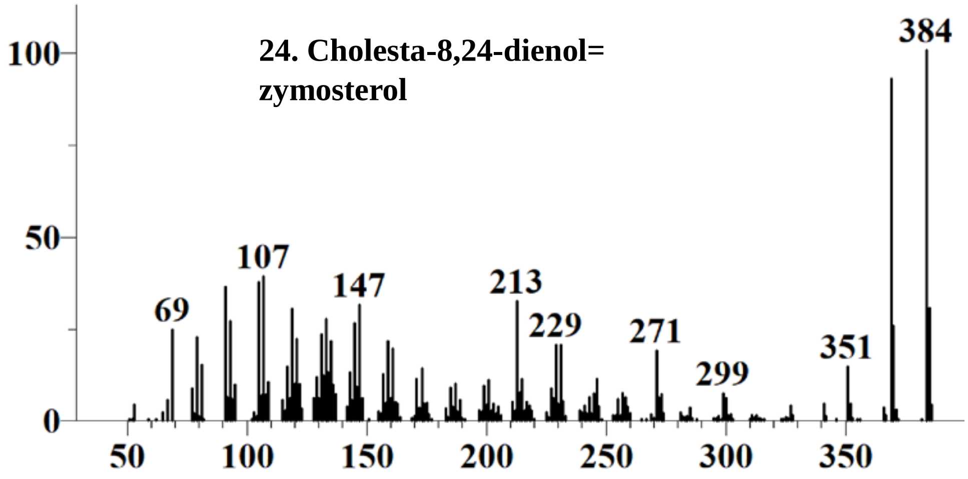

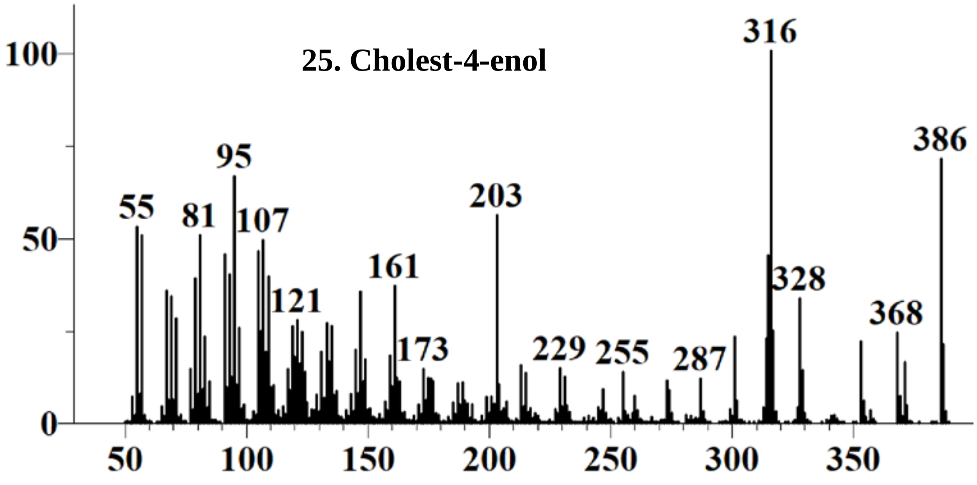

Supplementary Figure 12 (H)

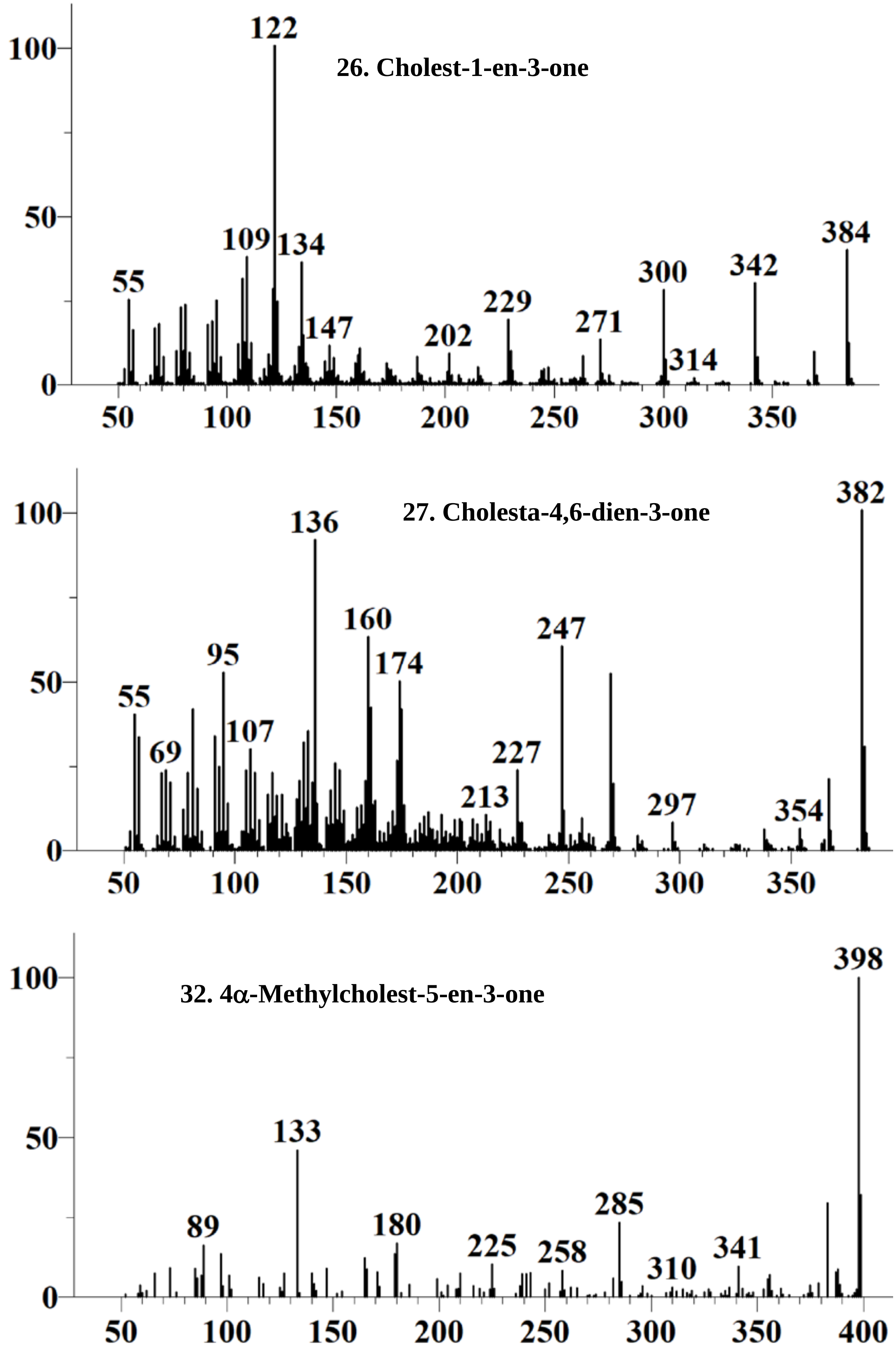

Supplementary Figure 12 (I)

Cholestanone + [$^{2}H_3$-*methyl*]methionine incubation:

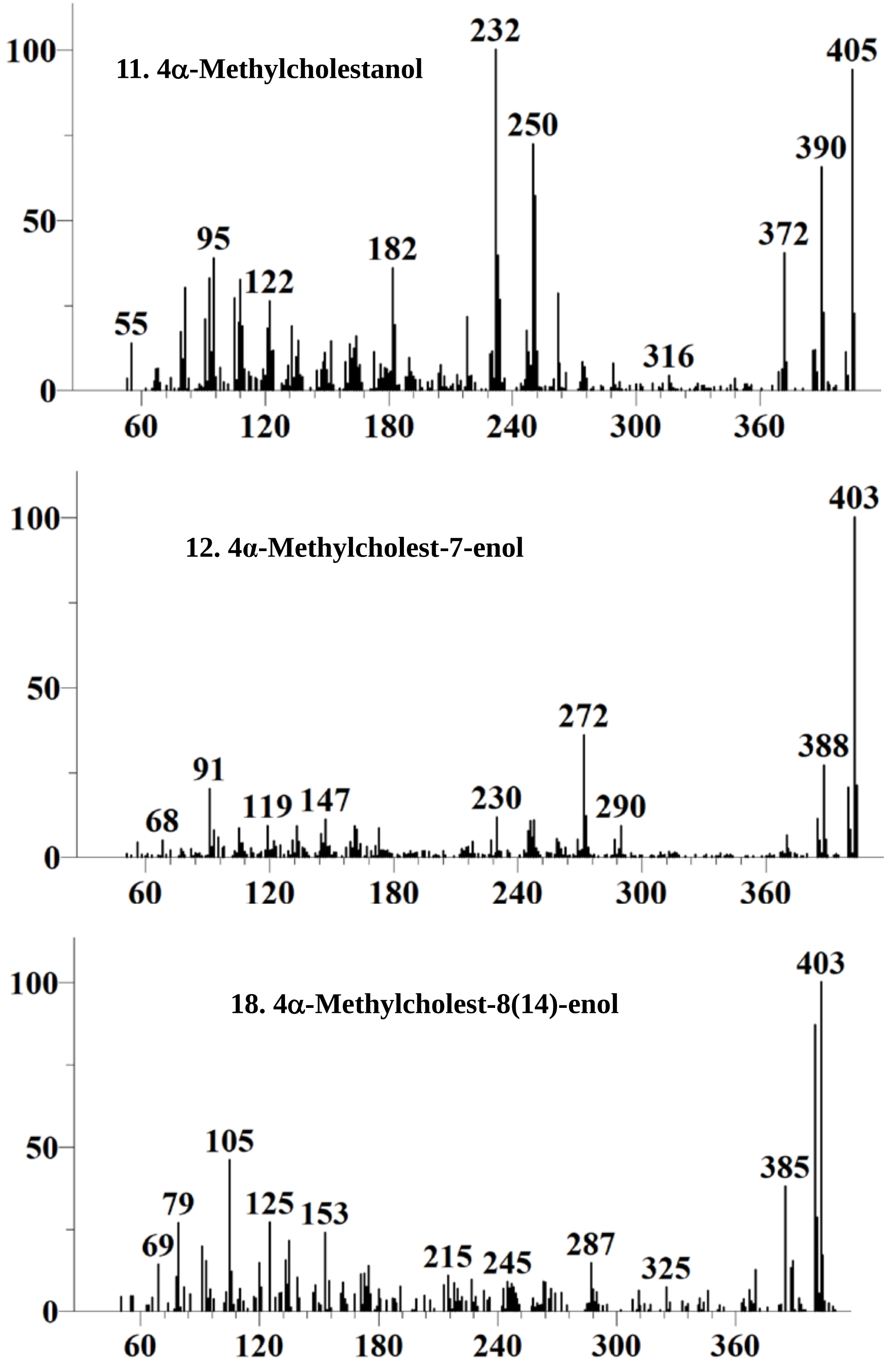

Supplementary Figure 12 (J)

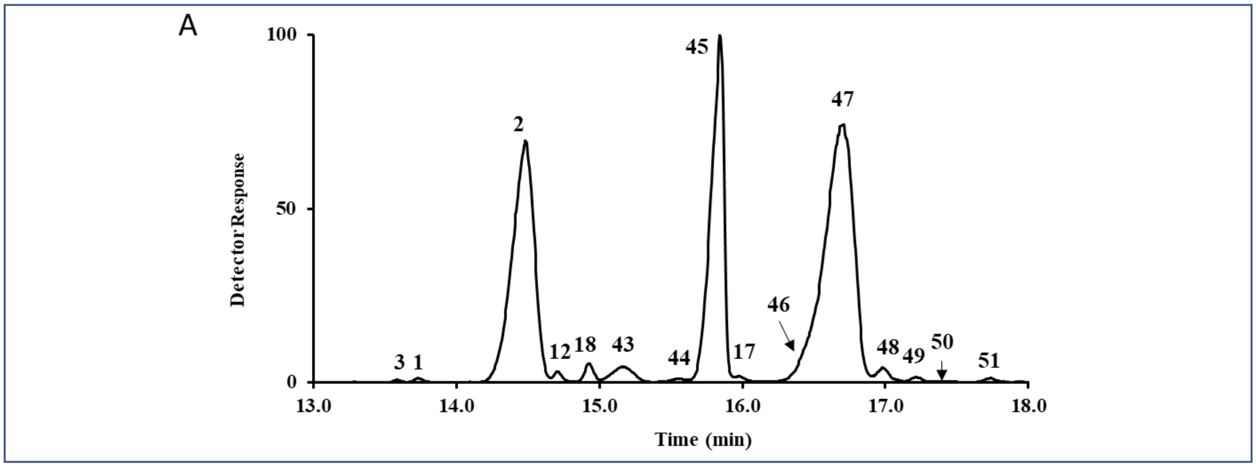

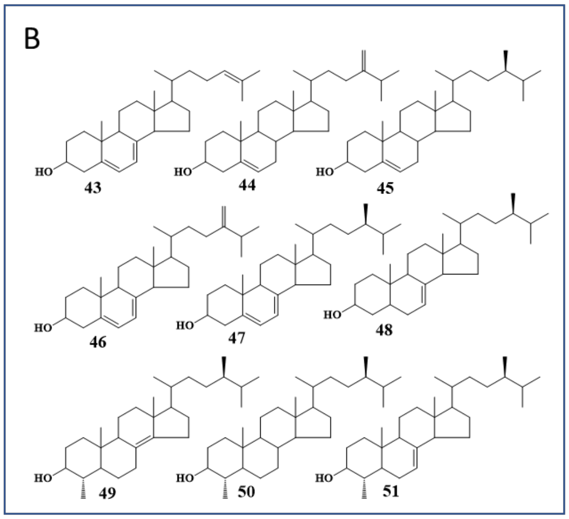

C. STable5. The sterol composition of *C. elegans* fed with campesterol

Sterols	Structure*	RRTC	M+	% of total
Cholesta-5,8(14)-dienol	3	0.99	384	0.3
Cholesterol	1	1.00	386	0.7
7-Dehydrocholesterol	2	1.05	384	27.8
Lathosterol	12	1.07	386	1.1
4α-Methylcholest-8(14)-enol	18	1.09	400	1.4
Cholesta-5,7,24-trienol	43	1.10	382	3.0
24-Methylenecholesterol	44	1.13	398	1.0
Campesterol	45	1.15	400	22.6
Ergosta-5,7,24(28)-trienol	46	1.16	396	0.6
4α-Methylcholest-7-enol	17	1.20	400	3.0
24α-Methylcholest-5,7-dienol	47	1.21	398	35.4
24α-Methylcholest-7-enol	48	1.24	400	1.7
4α,24α-Dimethylcholest-8(14)-enol	49	1.25	414	0.7
4α,24α-Dimethycholestanol	50	1.27	416	0.1
4α,24α-Dimethylcholest-7-enol	51	1.29	414	0.6

*See panel B for structure.

Supplementary Figure 13

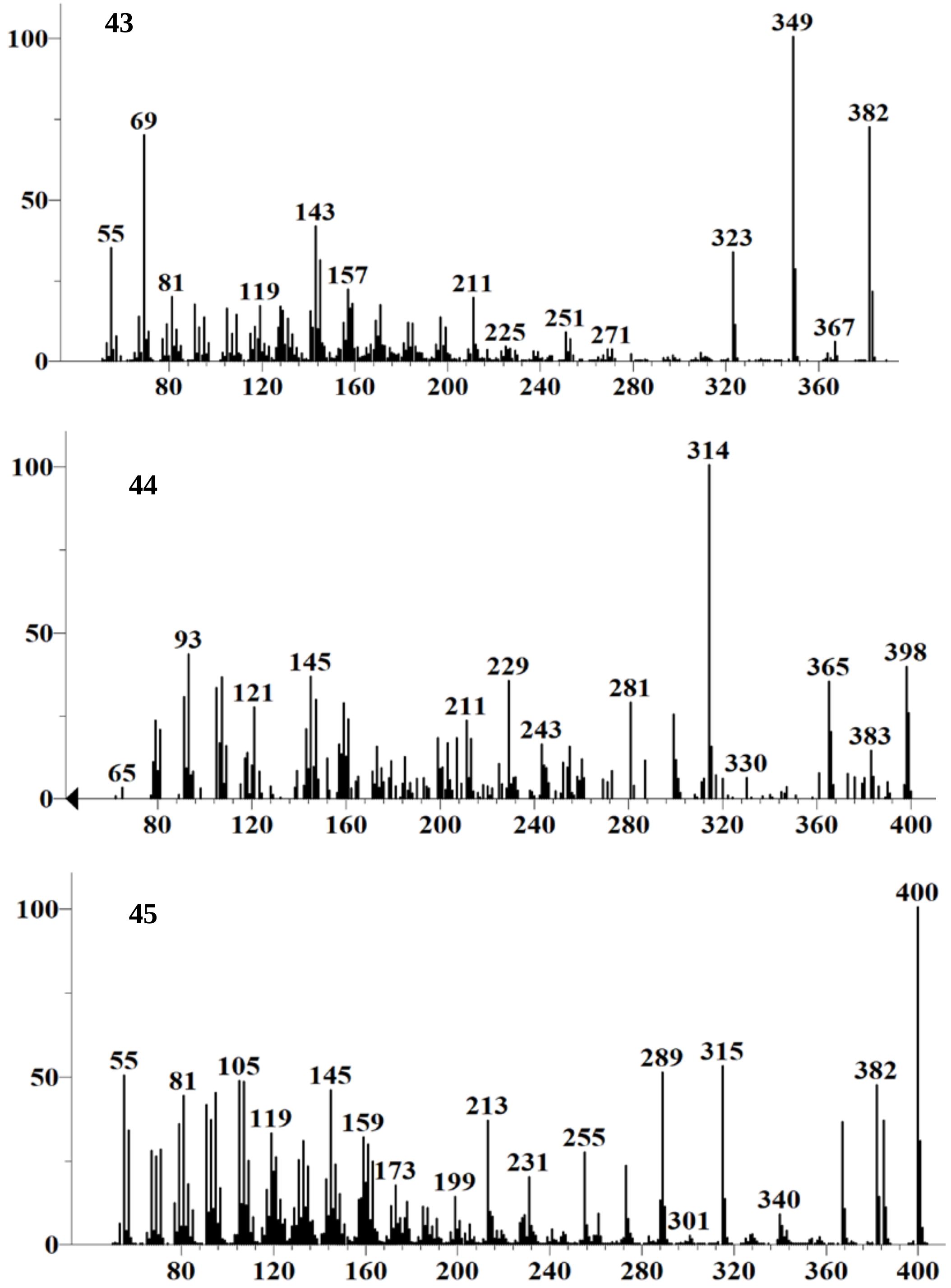

Supplementary Figure 14 (A)

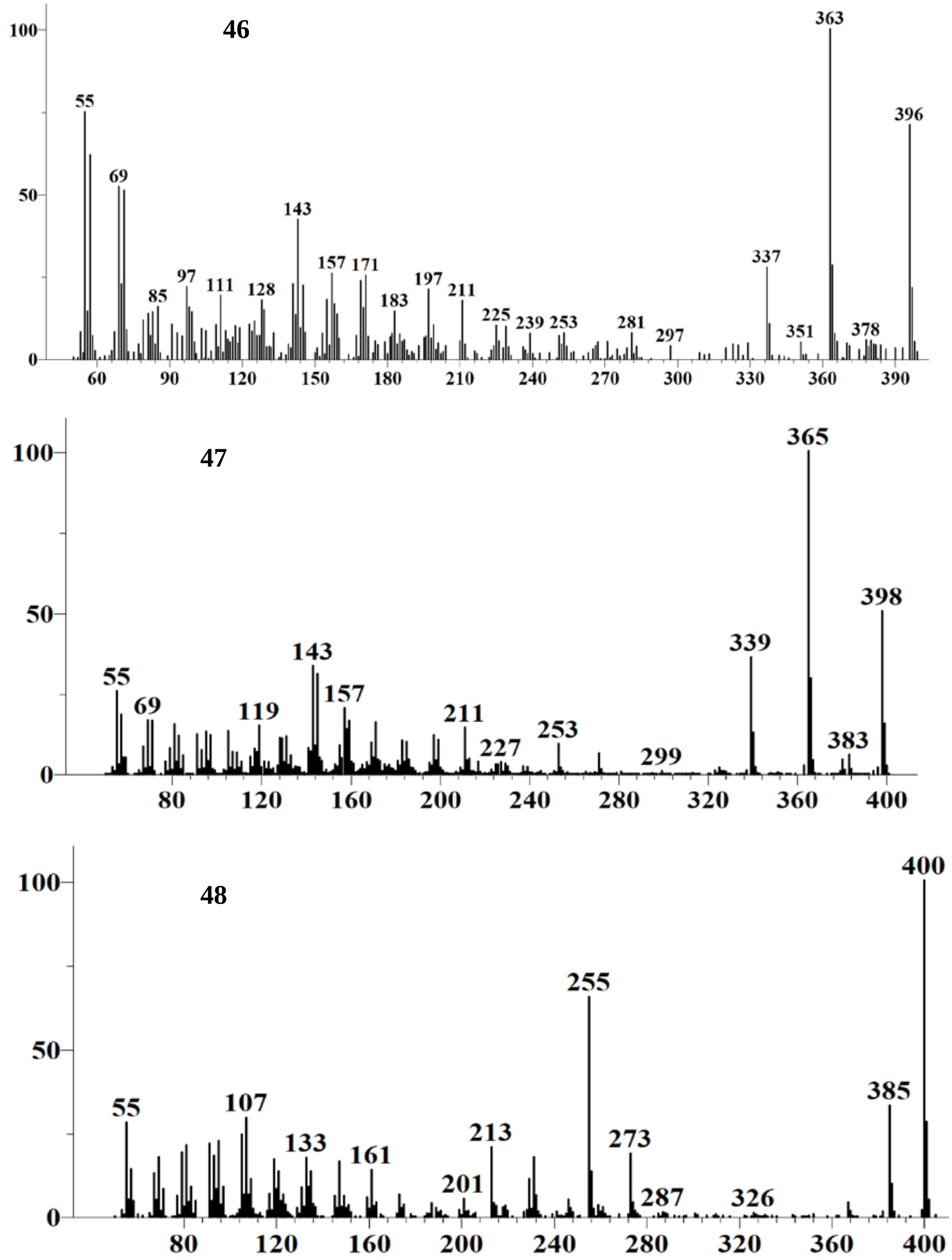

Supplementary Figure 14 (B)

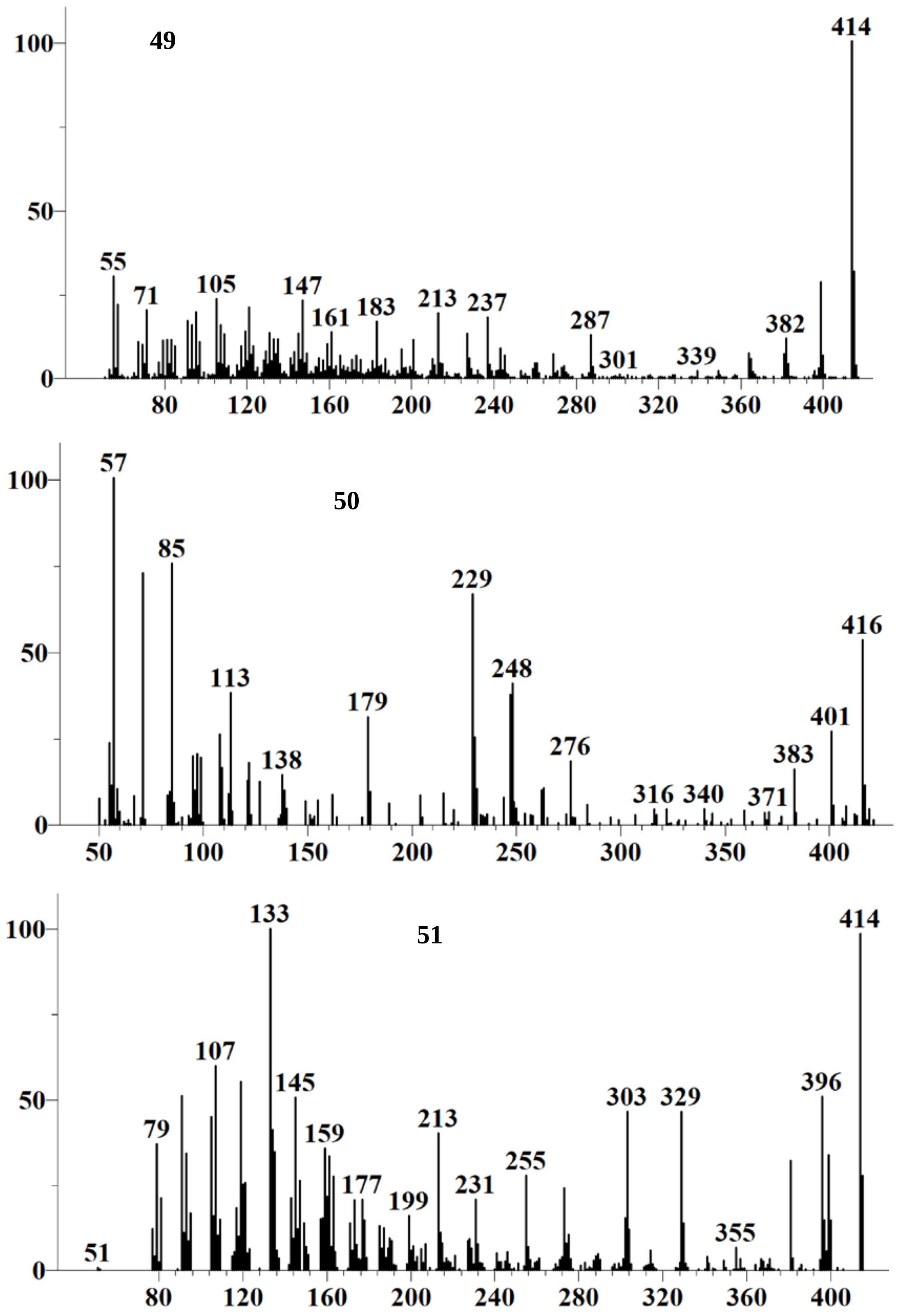

Supplementary Figure 14 (C)

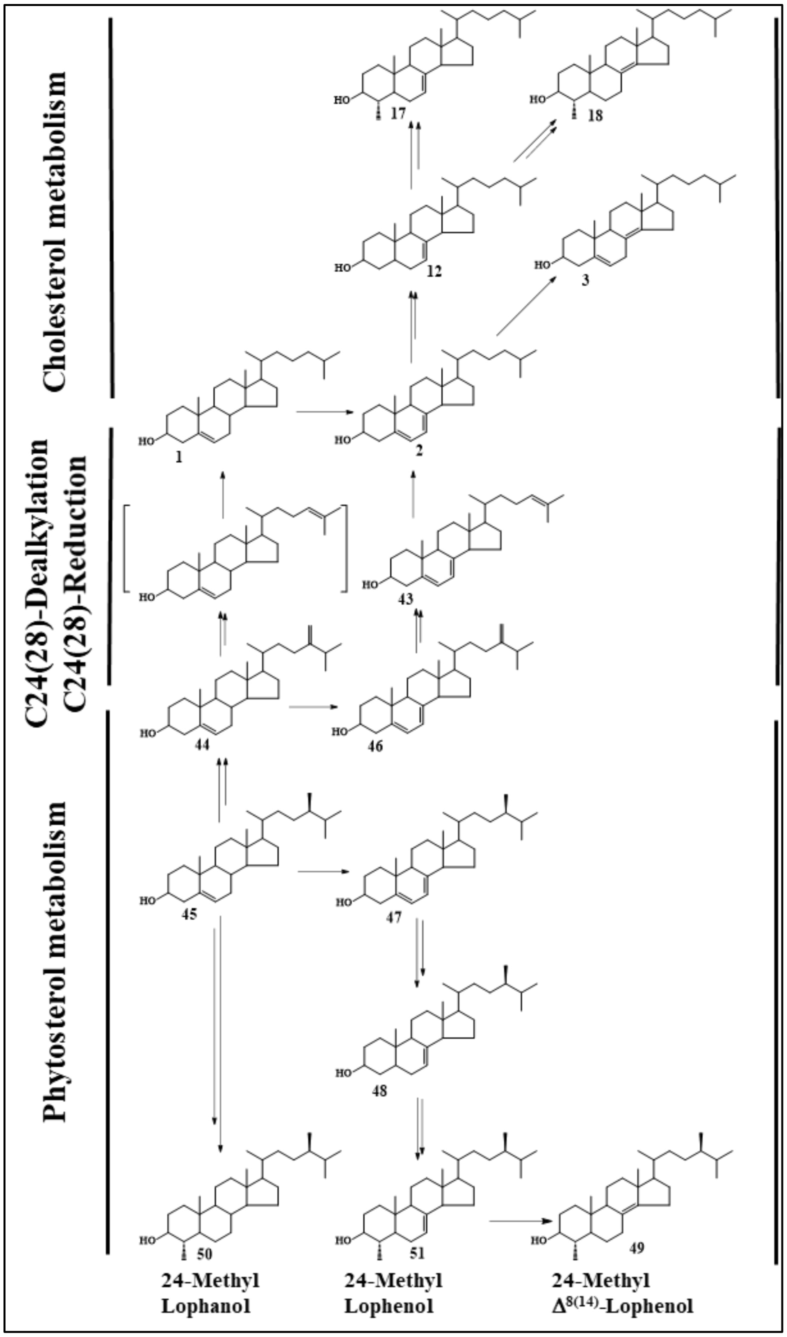

Supplementary Figure 15

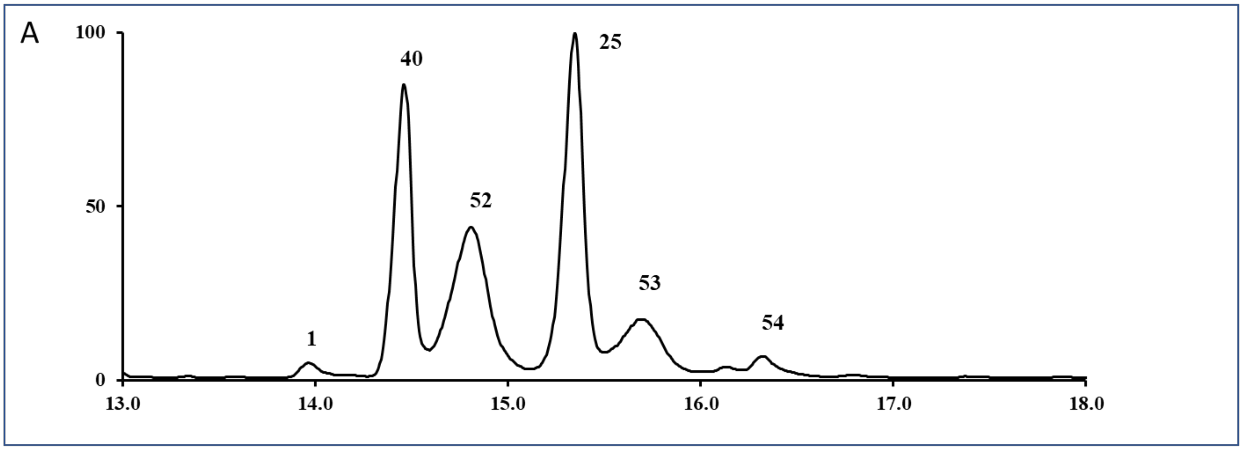

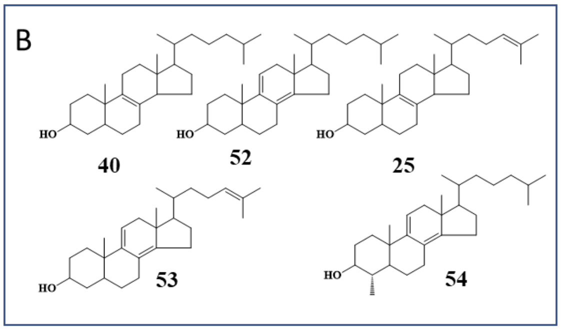

C. STable6. The sterol composition of *C. elegans* fed with zymosterol

**Sterols**	**Structure***	**RRTC**	**M+**	**% of total**
Cholesterol	1	1.00	386	1.7
Cholest-8-enol	40	1.05	386	27.0
Cholesta-9(11), 8(14)-dienol	52	1.07	384	27.7
Zymosterol	25	1.11	384	33.7
Cholesta-9(11),8(14),24-trienol	53	1.14	382	8.3
4α-Methylcholesta-9(11), 8(14)-dienol	54	1.17	398	1.6

*See panel B for structure.

Supplementary Figure 16

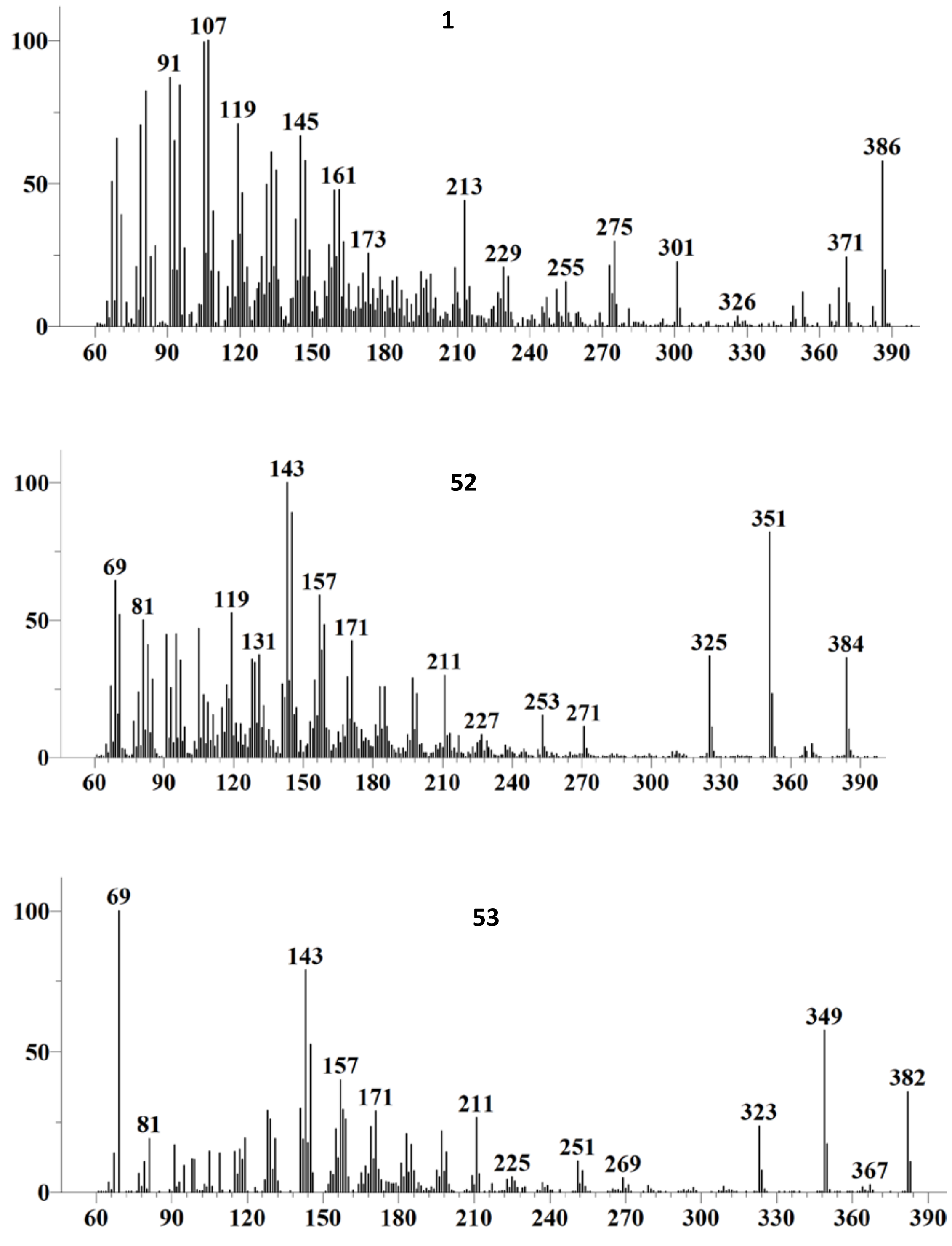

Supplementary Figure 17 (A)

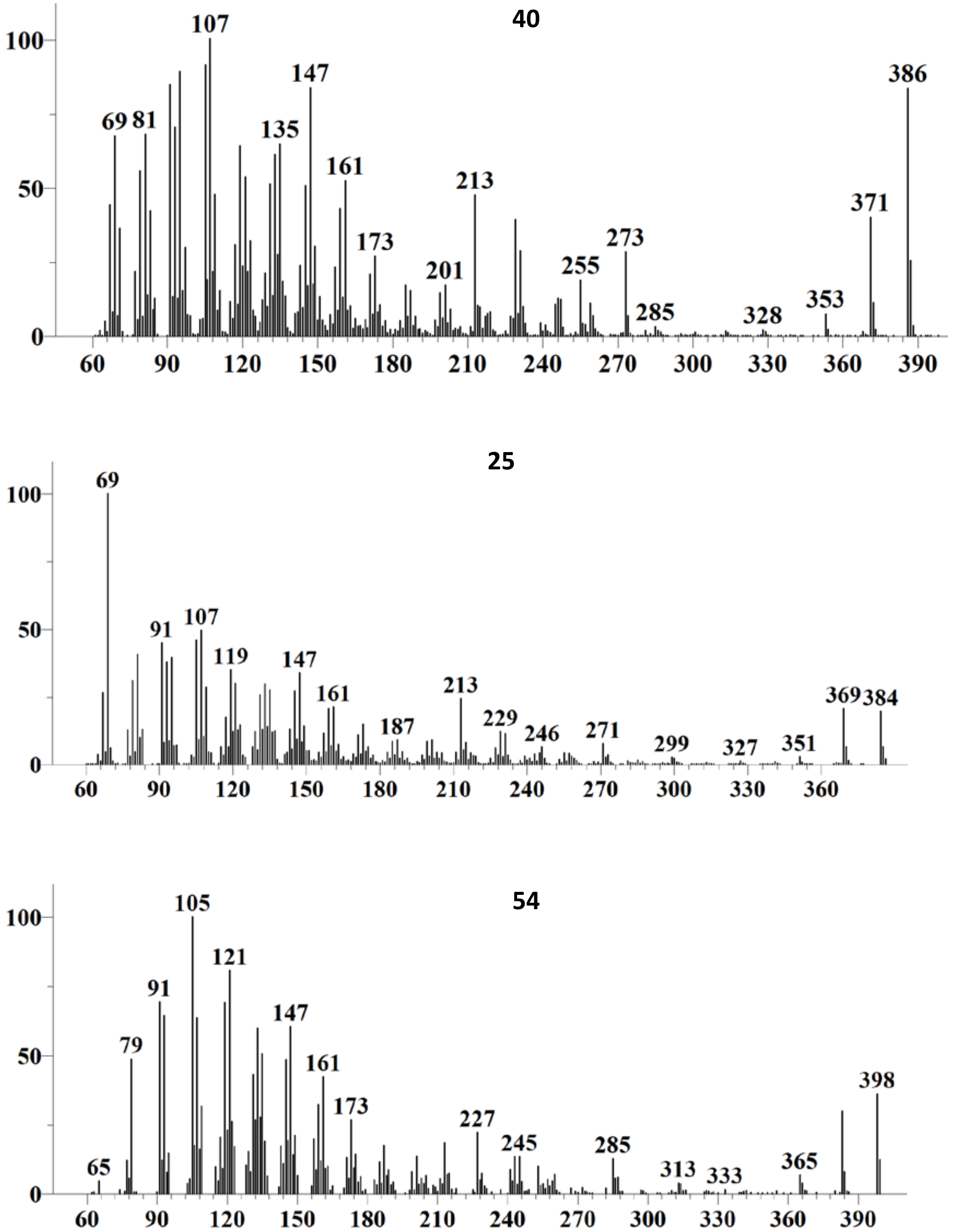

Supplementary Figure 17 (B)

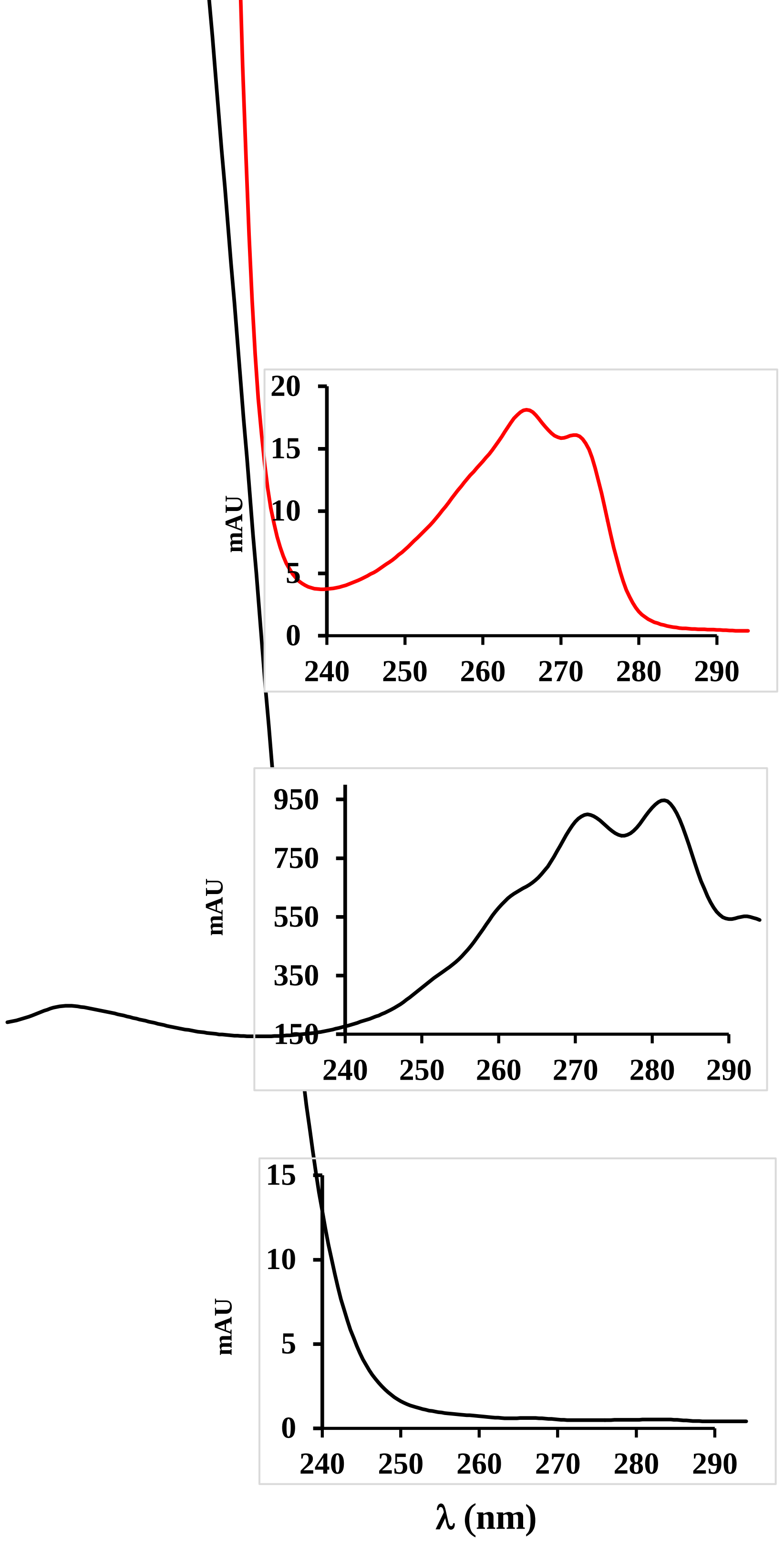

Supplementary Figure 17 (C).

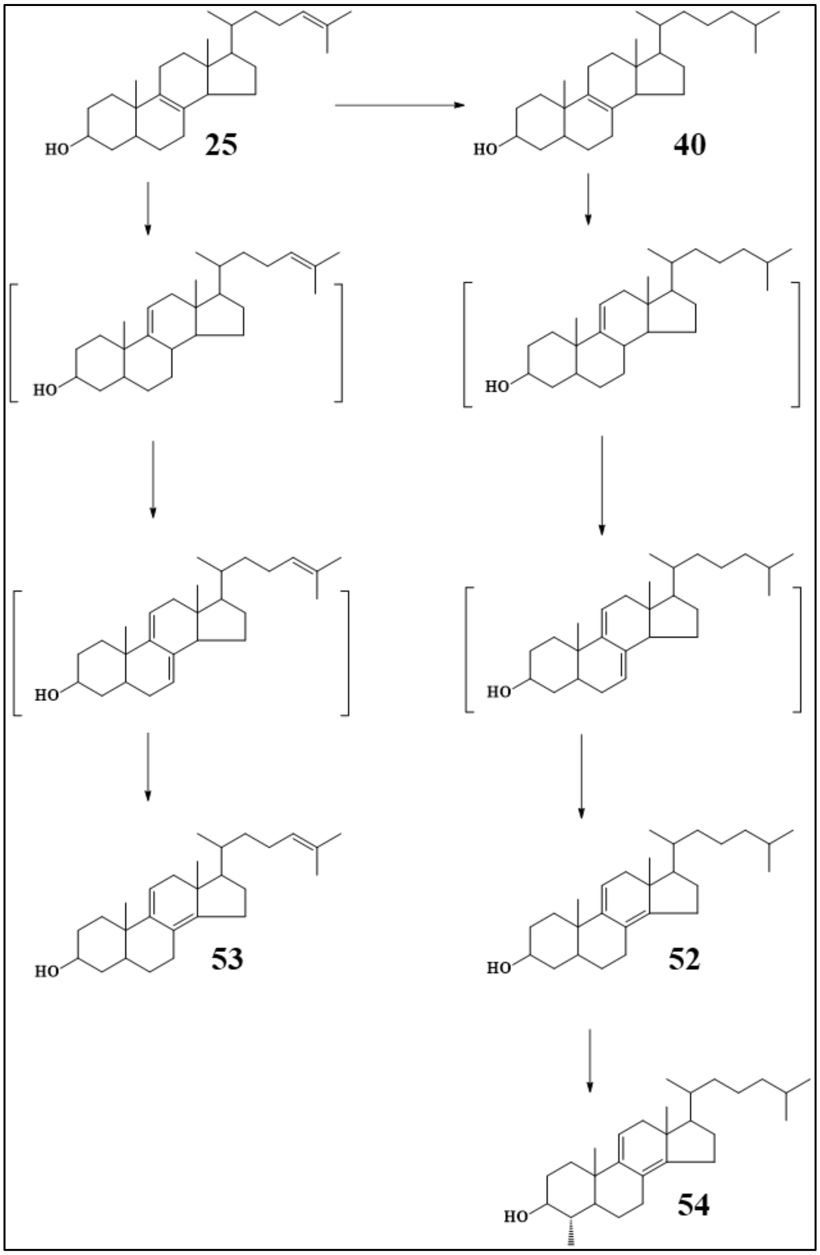

Supplementary Figure 18

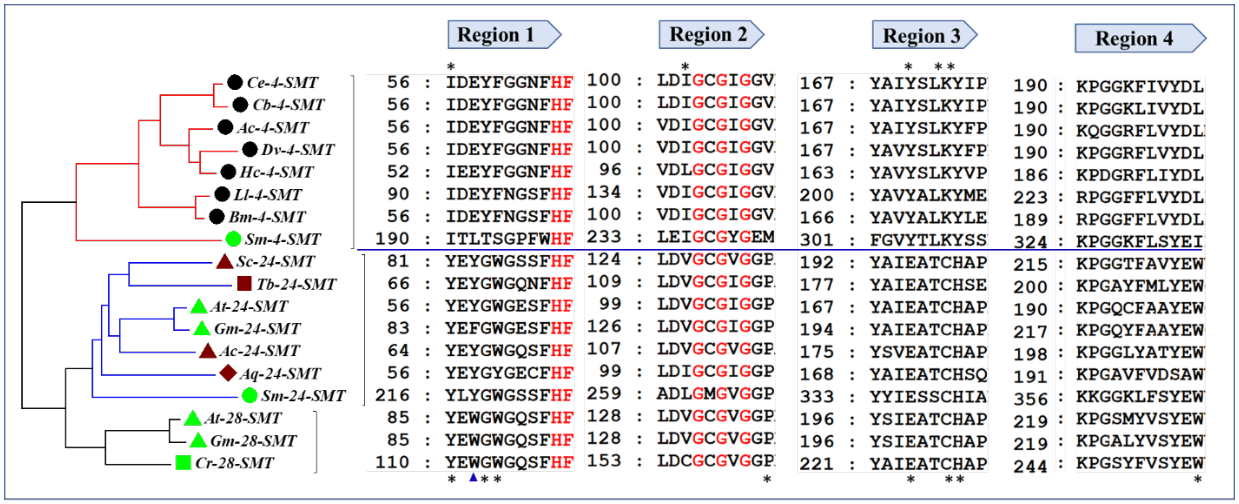

Supplementary Figure 19

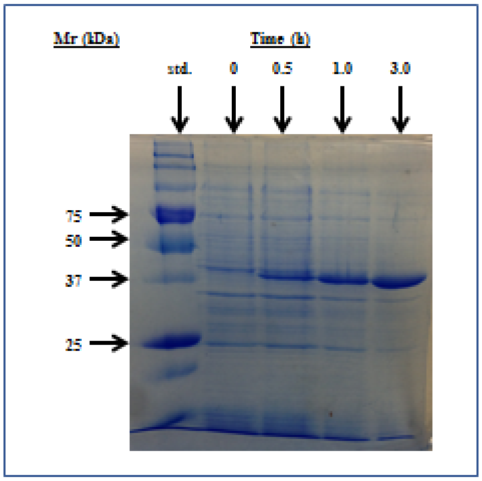

Supplementary Figure 20

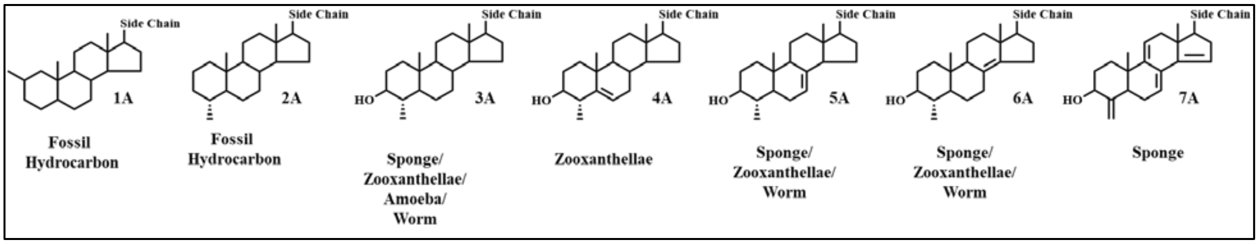

Supplementary Figure 21

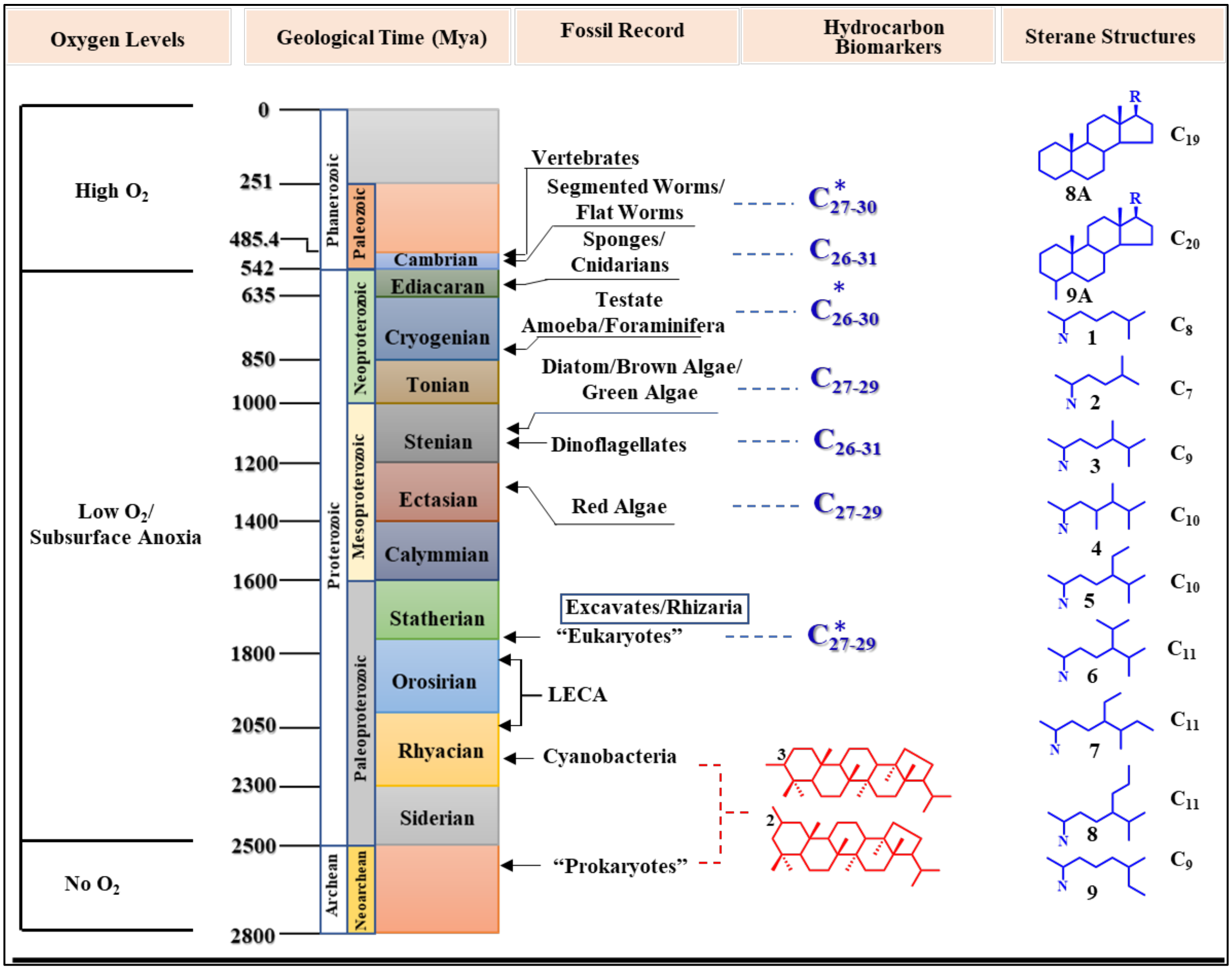

Supplementary Figure 22

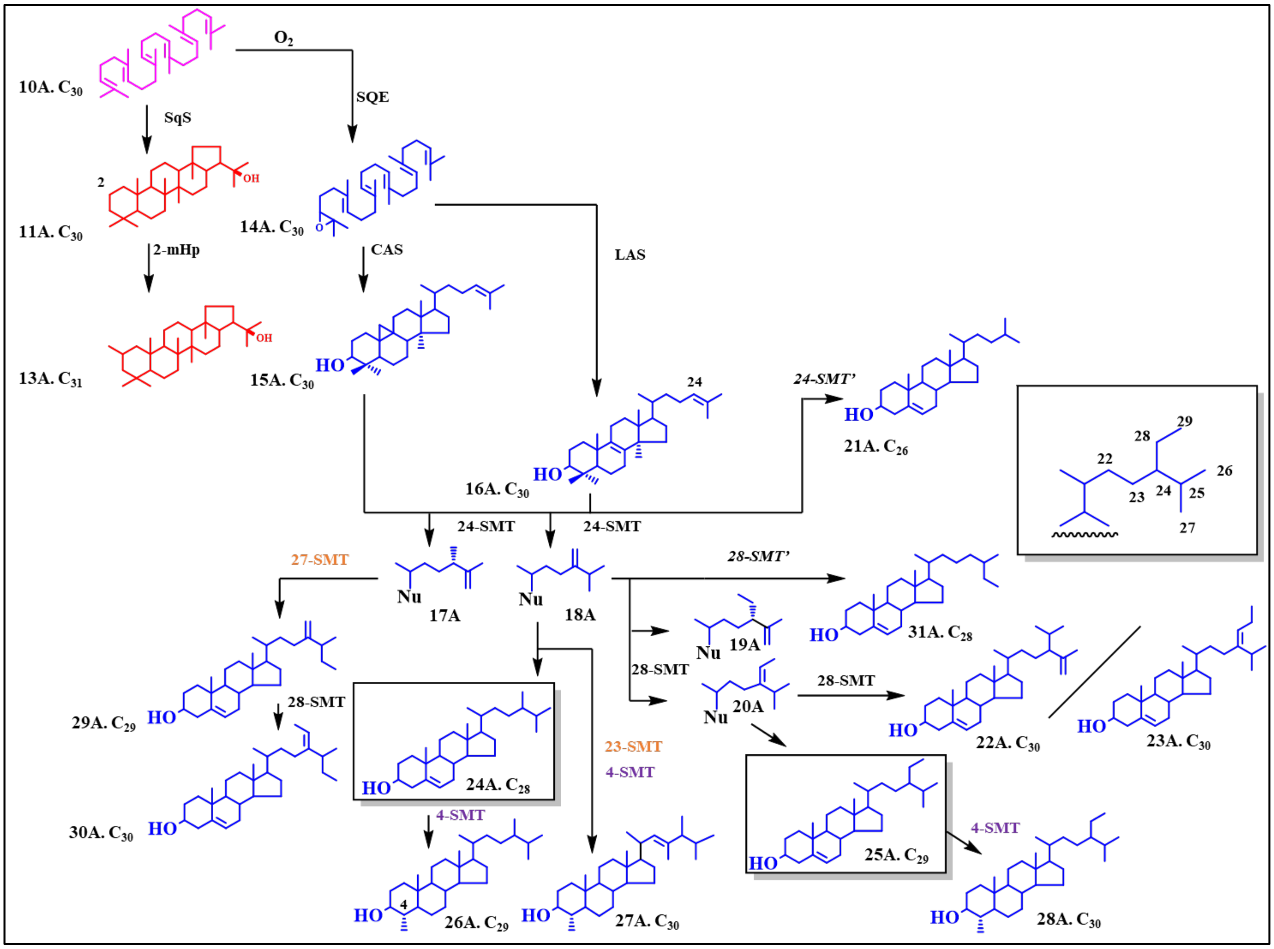

Supplementary Figure 23
